# Revisiting Fragmentation Reactions of Protonated α-Amino Acids by High-Resolution Electrospray Ionization Tandem Mass Spectrometry with Collision-Induced Dissociation

**DOI:** 10.1038/s41598-019-42777-8

**Published:** 2019-04-23

**Authors:** Pengwei Zhang, Wan Chan, Irene L. Ang, Rui Wei, Melody M. T. Lam, Kate M. K. Lei, Terence C. W. Poon

**Affiliations:** 1 Pilot Laboratory, Institute of Translational Medicine, Faculty of Health Sciences, University of Macau, Macau, China; 2 Department of Chemistry, Hong Kong University of Science and Technology, Hong Kong, China; 3 Proteomics Core, Faculty of Health Sciences, University of Macau, Macau, China

**Keywords:** Mass spectrometry, Mass spectrometry

## Abstract

Fragmentation reactions of protonated α-amino acids (AAs) were studied previously using tandem mass spectrometry (MS/MS) of unit mass resolution. Isobaric fragmentation products and minor fragmentation products could have been overlooked or misannotated. In the present study, we examined the fragmentation patterns of 19 AAs using high-resolution electrospray ionization MS/MS (HR-ESI-MS/MS) with collision-induced dissociation (CID). Isobaric fragmentation products from protonated Met and Trp were resolved and identified for the first time. Previously unreported fragmentation products from protonated Met, Cys, Gln, Arg, and Lys were observed. Additionally, the chemical identity of a fragmentation product from protonated Trp that was incorrectly annotated in previous investigations was corrected. All previously unreported fragmentation products and reactions were verified by pseudo MS^3^ experiments and/or MS/MS analyses of deuterated AAs. Clearer pictures of the fragmentation reactions for Met, Cys, Trp, Gln, Arg and Lys were obtained in the present study.

## Introduction

Tandem MS (MS/MS) based fragmentation reaction is an important tool for quantification, identity determination, structural elucidation and characterization of synthetic and natural products^1–3^. On one hand, accurate quantification of a molecule using selected reaction monitoring or multiple reaction monitoring requires a selection of an unambiguous representative fragmentation products for signal intensity measurement^4^. On the other hand, structural elucidation and characterization using MS/MS relies on understanding of fragmentation reaction of a charged molecule^3^. Without knowing correct chemical identities of fragmentation products, it is not possible to elucidate the fragmentation pathway. However, conventional MS/MS with unit mass resolution does not always allow clear assignment of chemical identities to the fragmentation products according to observed masses, particularly when differentiating isobaric fragments, e.g., fragmentation products due to neutral loss of CO (27.99491 Da), N_2_ (28.00615 Da), CH_2_N (28.01872 Da) or C_2_H_4_ (28.03130 Da).

In recent years, MS platforms that can perform high-resolution electrospray ionization MS/MS (HR-ESI-MS/MS) have been becoming popular. HR-ESI-MS/MS allows clear differentiation among isobaric fragments. Using HR-ESI-MS/MS, it was shown that MS/MS fragmentation pathway of rhodamine B that was previously constructed using unit mass resolution MS/MS data was not reliable because of the wrong annotations of the isobaric fragmentation products^5^. Therefore, it is necessary to use HR-ESI-MS/MS to resolve isobaric fragments and obtain their accurate masses for correct annotations of the fragmentation products.

Amino acid (AA) is a class of compounds present in every biological system. AA analysis is of particular importance in health and food science. Having a good knowledge of the fragmentation reactions of AAs is useful for their identifications and quantifications^6,7^. For example, through MS/MS study of AAs, characteristic and abundant fragments for AA quantification were recommended by Piraud *et al*.^7^. Moreover, investigation on fragmentation reactions of AAs also helps to interpret fragmentation patterns of AA derivatives and peptides^8–11^. However, in previous studies, fragmentation patterns of AAs were only examined using conventional unit resolution MS^7,9,12–15^. Isobaric fragmentation products could have been overlooked or misannotated.

In the present study, fragmentation patterns of protonated AAs were examined using HR-ESI-MS/MS with collision-induced dissociation (CID). With respect to the mass range (i.e., *m/z* 50 to 6000) of our HR-ESI-MS/MS system, we studied the fragmentation patterns of 18 proteinogenic AAs, but not those of glycine (75 Da) and alanine (89 Da). In addition, one non-proteinogenic AA, ornithine, was also studied. Ornithine is a homolog of Lys, which plays vital role in urea cycle and arginine metabolism. There is a demand for qualitative and quantitative analyses of ornithine in clinical applications using LC-MS/MS^16–18^. Using the HR-ESI-MS/MS with CID, previously incorrectly annotated fragment products and previously unreported fragment products from protonated AAs were identified in the present study. Together pseudo MS^3^ experiments^19^ and analyses of deuterated forms, fragmentation reactions of the protonated AAs were clearly elucidated.

## Results and Discussion

### Amino acids with sulfur-containing side chains

#### Methionine (Met)

**Figure 1:**
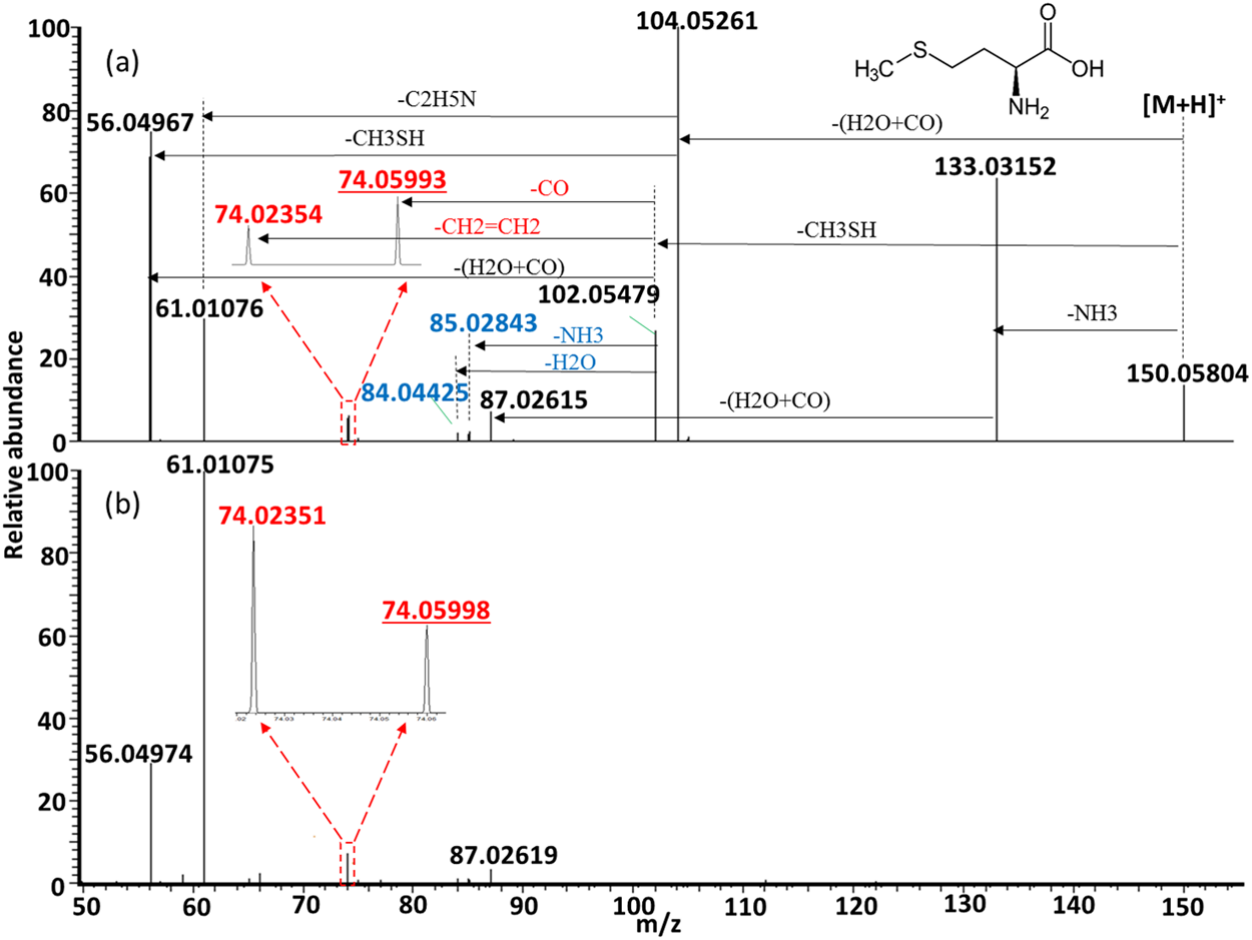
Representative MS/MS spectra of protonated Met acquired using collision energy NCE 30% (**a**) and 70% (**b**). The previously unreported fragment ions are shown in blue. Isobaric fragment ions are shown in red, and the underlined one was previously unreported.

**Figure 2:**
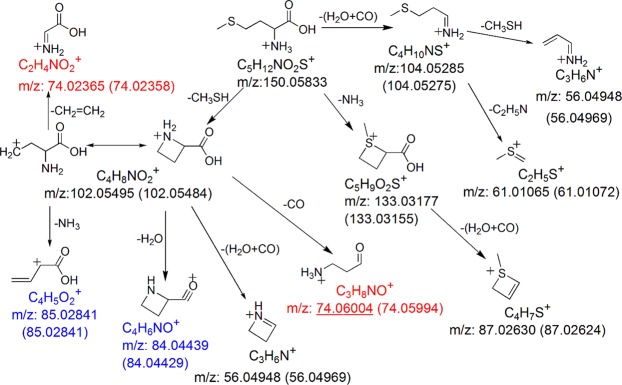
Postulated fragmentation pathways for protonated Met. The previously unreported fragment ions are shown in blue. Isobaric fragment ions are shown in red, and the underlined one was previously unreported. The theoretical *m/z* value of each fragment ion is provided under the chemical formula. The observed *m/z* values (mean calculated from 3 replicates) of the fragment ions are provided in the parentheses.

The observed fragment ions of protonated Met and their chemical identities are summarized in Supplementary Table S1. Energy-resolved fragmentation graph of protonated Met is provided as Supplementary Fig. S1. Fragmentation of protonated Met followed three pathways (Fig. 1). First, fragmentation started from the loss of H_2_O + CO. Subsequently, two fragments at *m/z* 61.01072 and 56.04969 were produced by further loss of C_2_H_5_N and CH_3_SH, respectively. Pseudo MS^3^ of the fragment ion at *m/z* 104.05275 from Met confirmed that [M + H−H_2_O−CO]^+^ was the origin of these 2 fragment ions (Supplementary Fig. S2). Second, fragmentation started from the loss of CH_3_SH, resulting in formation of [M + H−CH_3_SH]^+^ (*m/z* 102.05484). Subsequently, two previously unreported minor fragment ions at *m/z* 85.02841 and *m/z* 84.04429 were formed by losses of NH_3_ and H_2_O from [M + H−CH_3_SH]^+^, respectively, whereas two isobaric fragment ions at *m/z* 74.05994 and *m/z* 74.02358 were assigned as losses of CO and CH_2_ = CH_2_ from [M + H−CH_3_SH]^+^, respectively (Fig. 1a). Pseudo MS^3^ of the fragment ion at *m/z* 102.05484 confirmed that [M + H−CH_3_SH]^+^ was the origin of these 4 fragment ions and the fragment ion at *m/z* 56.04969 (Supplementary Fig. S3). This also revealed that the fragment ion at *m/z* 56.04969 could also be formed by loss of H_2_O + CO from [M + H−CH_3_SH]^+^. The presence of two different fragmentation pathways for the formation of *m/z* 56.04969 (i.e. from further dissociations of [M + H−CH_3_SH]^+^ and [M + H−H_2_O−CO]^+^) was confirmed by the MS/MS analysis of [Met-d_3_ + D]^+^, in which different mass shifts were observed for the fragment ion originally at *m/z* 56.04969 (Supplementary Fig. S4). Furthermore, the MS/MS analysis of [Met-d_3_ + D]^+^ also confirmed the fragmentation pathways for the formation of the fragment ions at *m/z* 85.02841, *m/z* 84.04429, *m/z* 74.05994 and *m/z* 74.02358 (Supplementary Fig. S4). The fragment ion originally at *m/z* 85.02841 did not shift its mass after the loss of CH_3_SD and ND_3_, and the fragment ion originally at *m/z* 84.04429 shifted to *m/z* 85.05064 after the loss of CH_3_SD and D_2_O. The fragment ion originally at *m/z* 74.05994 shifted to *m/z* 77.07883 corresponding to the loss of CH_3_SD and CO, and the fragment ion originally at *m/z* 74.02358 shifted to 75.02994 corresponding to the loss of CH_3_SD and CH_2_ = CD_2_. In a previous study, these two isobaric fragment ions at *m/z* 74.05994 (C_3_H_8_NO^+^) and *m/z* 74.02358 (C_2_H_4_NO_2_^+^) were only reported as a single fragment ion of the latter one^12^, i.e., C_2_H_4_NO_2_^+^. For the third fragmentation pathway, fragmentation of protonated Met started from the loss of NH_3_. The subsequent loss of H_2_O + CO resulted in the formation of a fragment ion at *m/z* 87.02624 (Fig. 1b). The postulated fragmentation pathways of protonated Met are summarized in Fig. 2.

#### Cysteine (Cys)

The observed fragment ions of protonated Cys and their chemical identities are summarized in Supplementary Table S1. Energy-resolved fragmentation graph of protonated Cys is provided as Supplementary Fig. S5. Fragmentation of protonated Cys followed two major and one minor pathways (Supplementary Fig. S6). First, fragmentation started from the loss of H_2_O + CO. Subsequently, one fragment at *m/z* 58.99525 was produced by further loss of NH_3_. Second, fragmentation started from the loss of NH_3_, followed by loss of H_2_O. Third, a minor fragment ion at *m/z* 88.03923, which was previously unreported, was assigned as [M + H−H_2_S]^+^, indicating the presence of a minor fragmentation pathway. The MS/MS analysis of [Cys-d_4_ + D]^+^ showed that the fragment ion originally at *m/z* 88.03923 shifted to *m/z* 91.05787 after the loss of D_2_S (Supplementary Fig. S7). This confirmed that the loss of H_2_S occurred in the fragmentation reaction of protonated Cys. The loss of H_2_S was previously observed in MS/MS fragmentation of cysteine radical cation and deprotonated Cys-containing peptides^8,10^, but not in the fragmentation studies of protonated Cys^7,12–15^. The postulated fragmentation pathways of protonated Cys are summarized in Supplementary Fig. S8.

### MS/MS fragmentation of branched-chain amino acids

#### Leucine (Leu), Isoleucine (Ile) and Valine (Val)

In consistent with previous studies^9,12^, three branched AAs (Leu, Ile and Val) produced only two fragments by sequential losses of H_2_O + CO and NH_3_ (Supplementary Fig. S9 to S15). The fragmentation patterns of protonated Ile and Leu were almost identical. However, under low collision energy (e.g., NCE 30%), the fragment ion at *m/z* 69 was only observed for protonated Ile (Supplementary Fig. S12). It was proposed that the fragment ion at *m/z* 69 could be used to differentiate between the isomeric Ile and Leu^9^. Our results showed that in the presence of sufficient collision energy, a fragment ion at *m/z* 69 could also be observed for Leu (Supplementary Fig. S10b). Therefore, when using *m/z* 69 as a diagnostic fragment ion for Ile, the employed analytical method should be carefully examined whether fragmentation of Leu could generate any fragment ion at *m/z* 69.

### MS/MS fragmentation of protonated aromatic amino acids

#### Tryptophan (Trp)

**Figure 3:**
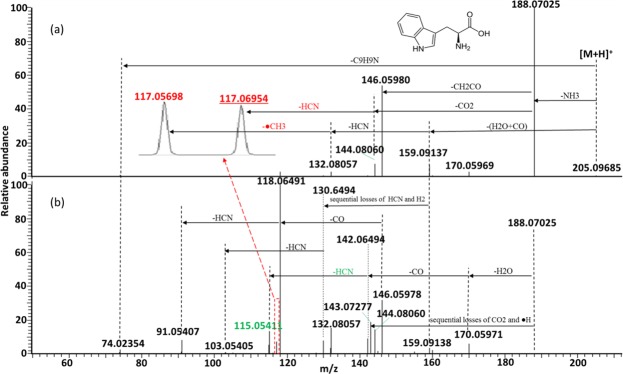
Representative MS/MS spectra of protonated Trp acquired using collision energy NCE 30% (**a**) and 70% (**b**). Isobaric fragment ions are shown in red, and the underlined one was previously unreported. The fragment ion that was incorrectly annotated in the previous investigation is shown in green.

**Figure 4:**
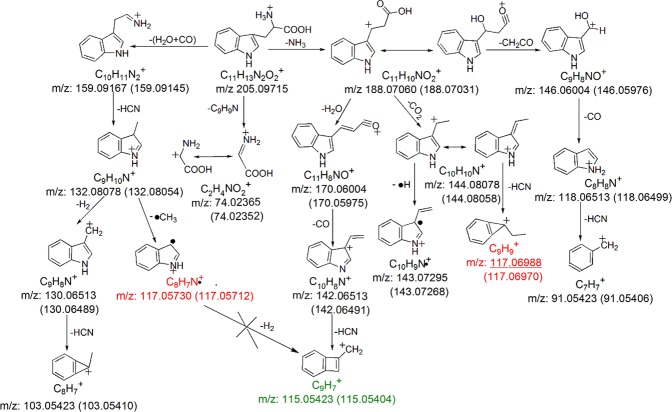
Postulated fragmentation pathways for protonated Trp. Isobaric fragment ions are shown in red, and the underlined one was previously unreported. The fragment ion that was incorrectly annotated in the previous investigations is shown in green. The theoretical *m/z* value of each fragment ion is provided under the chemical formula. The observed *m/z* values (mean calculated from 3 replicates) of the fragment ions are provided in the parentheses.

The observed fragment ions of protonated Trp and their chemical identities are summarized in Supplementary Table S1. Energy-resolved fragmentation graph of protonated Trp is provided as Supplementary Fig. S16. Fragmentation of protonated Trp followed one major pathway and two minor pathways (Fig. 3). Protonated Trp was predominantly dissociated to form a fragment ion at *m/z* 188.07031 upon the loss of NH_3_. Under low collision energy, one major fragmentation product at *m/z* 146.05976 and one minor fragmentation product at *m/z* 144.08058 were formed after the further loss of CH_2_CO and loss of CO_2_, respectively (Fig. 3a). The loss of CH_2_CO involved an intramolecular hydroxyl migration from the carboxylic group to the *β*-carbon^14^. The major fragmentation product at *m/z* 146.05976 further dissociated to form a fragment ion at *m/z* 118.06499 upon the loss of CO. Finally, an additional loss of HCN resulted in the formation of a fragment ion at *m/z* 91.05406. For the minor fragmentation product at *m/z* 144.08058, it further dissociated to form a fragment ion at *m/z* 117.06970 upon the loss of HCN. Under high collision energy, most of the fragmentation products at *m/z* 146.05976 dissociated to form the fragment ion at *m/z* 118.06499, and that became the predominant fragmentation product (Fig. 3b). At the same time, a minor portion of the fragment ions at *m/z* 188.07031 dissociated to form the fragment ions at *m/z* 170.05975 and *m/z* 143.07268 upon the loss of H_2_O and sequential losses of CO_2_ and •H, respectively. A fragment ion at *m/z* 115.05404 was formed after the sequential losses of CO and HCN from the fragment ions at *m/z* 170.05975. Previously, this fragment ion was incorrectly assigned as loss of •CH_3_ and H_2_ from [M + H−H_2_O−CO−HCN]^+^ in a study using low resolution MS/MS^14^. Its chemical identity was confirmed through MS/MS analysis of [Trp-d_4_ + D]^+^, in which its *m/z* value shifted to 117.06685, corresponding to the loss of DCN from the [M + H−ND_2_H−DHO−CO]^+^ (Supplementary Fig. S17). For the two minor fragmentation pathways, dissociation of protonated Trp started from generation of fragment ions at *m/z* 159.09145 and *m/z* 74.02352 through the loss of H_2_O + CO and loss of C_9_H_9_N, respectively. Sequential losses of HCN and •CH_3_ from the fragment ion at *m/z* 159.09145 resulted in the formation of a fragment ion at *m/z* 117.05712. In a previous report, the two isobaric fragment ions at *m/z* 117.06970 and *m/z* 117.05712 were not resolved, and only the chemical identity of [M + H−H_2_O−CO−HCN−•CH_3_]^+^ was assigned^14^. According to our analysis, fragment at *m/z* 117.06970 was produced by the elimination of HCN from [M + H−NH_3_−CO_2_]^+^ (as mentioned above). This finding was confirmed by the MS/MS analysis of [Trp-d_4_ + D]^+^. The isobaric fragments originally at *m/z* 117.06970 and *m/z* 117.05712 shifted to *m/z* 119.08252 and *m/z* 119.06995, corresponding to the losses of ND_2_H, CO_2_ and DCN and the losses of DHO + CO, DCN and •CDH_2_, respectively (Supplementary Fig. S17). Taken together with findings from the previous report^14^, the postulated fragmentation pathways of protonated Trp are summarized in Fig. 4.

#### Phenylalanine (Phe)

The observed fragment ions of protonated Phe and their chemical identities are summarized in Supplementary Table S1. Energy-resolved fragmentation graph of protonated Phe is provided as Supplementary Fig. S18. Fragmentation of protonated Phe followed one major and one minor pathways (Supplementary Fig. S19). The major fragmentation pathway of protonated Phe started from the loss of H_2_O + CO to form a fragment ion at *m/*z 120.08061 (Supplementary Fig. S19a). A fragment ion at *m/z* 103.05408 was formed by the further loss of NH_3_. Under high collision energy, the fragment ion at *m/z* 120.08061 dissociated to form a minor fragment ion at *m/z* 118.06496 upon the loss of H_2_, whereas fragment ions at *m/z* 93.06978 and *m/z* 91.05406 were formed by the sequential losses of HCN and H_2_ from the fragment ion at *m/z* 120.08061 (Supplementary Fig. S19b). The minor fragmentation pathway started from the loss of NH_3_ to form a fragment ion at *m/z* 149.05952 (Supplementary Fig. S19a). Sequential losses of H_2_O and CO from the fragment ion at *m/z* 149.05952 resulted in the formation of two fragment ions at *m/z* 131.04895 and *m/z* 103.05408. Under high collision energy, the fragment ion at *m/z* 149.05952 dissociated to form two fragment ions at *m/z* 107.04899 and *m/z* 79.05411 upon the sequential losses of CH_2_CO and CO (Supplementary Fig. S19b). The two fragmentation pathways for the formation of the fragment ion at *m/z* 103.05408 were confirmed using pseudo MS^3^ analyses of its precursors at *m/*z 120.08061 and *m/z* 131.04895 (Supplementary Fig. S20). However, in previous studies, only one of these pathways was proposed^7,12,14^. Taken together with the previous investigations^12,14^, the postulated fragmentation pathways of protonated Phe are summarized in Supplementary Fig. S21.

#### Tyrosine (Tyr)

The observed fragment ions of protonated Tyr and their chemical identities are summarized in Supplementary Table S1. Energy-resolved fragmentation graph of protonated Tyr is provided as Supplementary Fig. S22. Fragmentation of protonated Tyr followed two pathways (Supplementary Fig. S23). First, a fragment ion at *m/z* 165.05434 was formed by the loss of NH_3_. Subsequently, two fragment ions at *m/z* 147.04392 and *m/z* 123.04391 were formed by the loss of H_2_O and the loss of CH_2_CO, respectively (Supplementary Fig. S23a). Sequential losses of two neutral fragments of CO from the fragment ion at *m/z* 147.04392 resulted in the formation of two fragment ions at *m/z* 119.04909 and *m/z* 91.05411. Under high collision energy, the fragment ion at *m/z* 123.04391 dissociated to form a fragment ion at *m/z* 95.04904 with the loss of CO, followed by formation of a fragment ion at *m/z* 65.03868 upon the loss of CH_2_O (Supplementary Fig. S23b). Second, the loss of H_2_O + CO from protonated Tyr led to the formation of a fragment ion at *m/z* 136.07542 (Supplementary Fig. S23a). Further loss of NH_3_ resulted in the formation of the fragment ion at *m/z* 119.04909. Under high dissociation energy, the fragment ion at *m/z* 136.07542 was also dissociated to form a fragment ion at *m/z* 107.04904 with the concomitant loss of HCN and H_2_ (Supplementary Fig. S23b). The observed fragmentations of protonated Tyr were consistent with those reported previously^14^. However, the presence of two fragmentation pathways for the formation of the fragment ion at *m/z* 119.04909 was not shown in previous studies^7,12,14^. Moreover, they were further confirmed using pseudo MS^3^ analysis of its precursors at *m/z* 136.07542 and *m/z* 147.04392 (Supplementary Fig. S24). Together with the previous investigations^7,12,14^, the postulated fragmentation pathways of protonated Tyr are summarized in Supplementary Fig. S25.

### Amino acids with polar uncharged side chains

#### Glutamine (Gln)

The observed fragment ions of protonated Gln and their chemical identities are summarized in Supplementary Table S1. Energy-resolved fragmentation graph of protonated Gln is provided as Supplementary Fig. S26. Fragmentation of protonated Gln followed one major pathway and one minor pathway (Supplementary Fig. S27). The major fragmentation pathway of protonated Gln started from the loss of NH_3_ to form a fragment ion at *m/z* 130.04968 (Supplementary Fig. S27a). Subsequent loss of H_2_O + CO resulted in the formation of a predominant fragment ion at *m/z* 84.04428. Under high collision energy, it further dissociated to form a fragment ion at *m/z* 56.04972 with the loss of CO (Supplementary Fig. S27b). In addition, a previously unreported minor fragment at *m/z* 102.05480 was formed by a concomitant loss of NH_3_ + CO (Supplementary Fig. S27). Pseudo MS^3^ analysis of [M + H−NH_3_]^+^ of Gln showed that there was no sign of elimination of CO from [M + H−NH_3_]^+^ (Supplementary Fig. S28). Thus, the concomitant loss of NH_3_ and CO is involved in this process, which is different from the two-step characteristic losses of NH_3_ and CO from the C-terminal Gln residue of sodiated peptides^20^. Additionally, the chemical identity of this fragment was confirmed using MS/MS analysis of [Gln-d_5_ + D]^+^, in which it shifted to *m/z* 105.07370 by a concomitant loss of ND_3_ and CO (Supplementary Fig. S29). For the minor fragmentation pathway, the loss of H_2_O + CO from protonated Gln resulted in the formation of a fragment ion at *m/z* 101.07084. Taken together with previous investigations^7,12^, the postulated fragmentation pathways of protonated Gln are summarized in Supplementary Fig. S30.

#### Asparagine (Asn)

The observed fragment ions of protonated Asn and their chemical identities are summarized in Supplementary Table S1. Energy-resolved fragmentation graph of protonated Asn is provided as Supplementary Fig. S31. Fragmentation of protonated Asn followed two pathways (Supplementary Fig. S32). First, fragmentation started from the loss of H_2_O + CO, resulting in formation a fragment ion at *m/z* 87.05521 (Supplementary Fig. S32a). Under high collision energy, a fragment ion at *m/z* 70.02874 was produced after further loss of NH_3_ (Supplementary Fig. S32b). Second, fragmentation started from the loss of NH_3_, followed by the loss of CO and the loss of CH_2_CO, resulting in formation of two fragment ions at *m/z* 88.03928 and 74.02359, respectively (Supplementary Fig. S32a). Taken together with the previous reports^7,12^, the postulated fragmentation pathways of protonated Asn are summarized in Supplementary Fig. S33.

#### Threonine (Thr) and Serine (Ser)

In consistent with previous studies^7,13,21^, fragmentation reactions of protonated Thr and Ser were similar (Supplementary Fig. S34 to S39). Fragmentation products were formed by the losses of H_2_O, 2H_2_O and H_2_O + CO. The major difference was that fragmentation of protonated Thr could produce two more fragmentation products due to the loss of H_2_O from [M + H−H_2_O−CO]^+^ and loss of NH_3 + _CO from [M + H]^+^.

### Amino acids with negatively charged side chains

#### Glutamic acid (Glu)

The observed fragment ions of protonated Glu and their chemical identities are summarized in Supplementary Table S1. Energy-resolved fragmentation graph of protonated Glu is provided as Supplementary Fig. S40. Fragmentation of protonated Glu followed two pathways (Supplementary Fig. S41). First, fragmentation of protonated Glu mainly started from the loss of H_2_O to form a fragment ion at *m/z* 130.04966 (Supplementary Fig. S41a). A fragment ion at *m/z* 84.04427 was produced by a subsequent elimination of H_2_O + CO. This dissociated step was confirmed by pseudo MS^3^ analysis (Supplementary Fig. S42a). Second, the sequential losses of H_2_O + CO and H_2_O from protonated Glu led to the formation of two fragment ions at *m/z* 102.05478 and *m/z* 84.04427 (Supplementary Fig. S41a). The elimination of H_2_O from the fragment ions at *m/z* 102.05478 was confirmed by pseudo MS^3^ analysis (Supplementary Fig. S42b). It has been proposed that the fragment ion at *m/z* 84 is originated from either the fragment ion at *m/z* 130 or the fragment ion at *m/z* 102^7,12^. Herein, we provide the first piece of evidence revealing that both fragment ions at *m/z* 130 and at *m/z* 102 are the precursors of the fragment ion at *m/z* 84. Under high collision energy, the fragment ion at *m/z* 84.04427 dissociated to form a fragment ion at *m/z* 56.04970 with the loss of CO (Supplementary Fig. S41b). Taken together with previous reports^7,12,13^, the postulated fragmentation pathways of protonated Glu are shown in Supplementary Fig. S43.

#### Aspartic acid (Asp)

The observed fragment ions of protonated Asp and their chemical identities are summarized in Supplementary Table S1. Energy-resolved fragmentation graph of protonated Asp is provided as Supplementary Fig. S44. MS/MS of protonated Asp generated a fragmentation pattern which was highly similar to that of Asn (Supplementary Figs S32 and S45). Fragmentation of protonated Asp followed two pathways. First, the elimination of H_2_O + CO led to the formation of a fragment ion at *m/z* 88.03914. Second, sequential losses of H_2_O and CH_2_CO from protonated Asp led to the formation of two fragment ions at *m/z* 116.03401 and *m/z* 74.02362. The postulated fragmentation pathways of protonated Asp are shown in Supplementary Fig. S46. This is consistent with previous investigations^12,13^.

### Amino acids with positively charged side chains

#### Histidine (His)

The observed fragment ions of protonated His and their chemical identities are summarized in Supplementary Table S1. Energy-resolved fragmentation graph of protonated His is provided as Supplementary Fig. S47. Fragmentation of protonated His followed one major and one minor pathways (Supplementary Fig. S48). Under low collision energy, the major MS/MS fragmentation pathway of protonated His started from loss of H_2_O + CO, resulting in a fragment ion at *m/z* 110.07114 (Supplementary Fig. S48a). Under high collision energy, fragment ions at *m/z* 93.04460, 83.06021, 82.05242 and 81.04459 were formed by the further losses of NH_3_, HCN, HCN + •H and HCN + H_2_, respectively. Under high collision energy, the fragment ion at *m/z* 83.06021 further dissociated to generate a fragment at *m/z* 56.04971 upon the elimination of HCN (Supplementary Fig. S48b). For the minor fragmentation pathway, a concomitant loss of NH_3_ and CO_2_ from protonated His resulted in the formation of a fragment ion at *m/z* 95.06013, which further dissociated to generate a fragment ion at *m/z* 68.04949 upon the loss of HCN (Supplementary Fig. S48b). Taken together with a previous report^14^, the postulated fragmentation pathways of protonated His are summarized in Supplementary Fig. S49.

#### Arginine (Arg)

**Figure 5:**
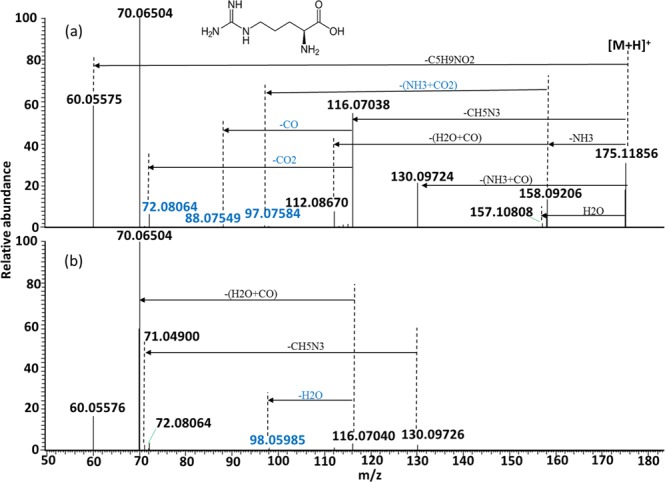
Representative MS/MS spectra of protonated Arg acquired using collision energy NCE 30% (**a**) and 70% (**b**). The previously unreported fragment ions are shown in blue.

**Figure 6:**
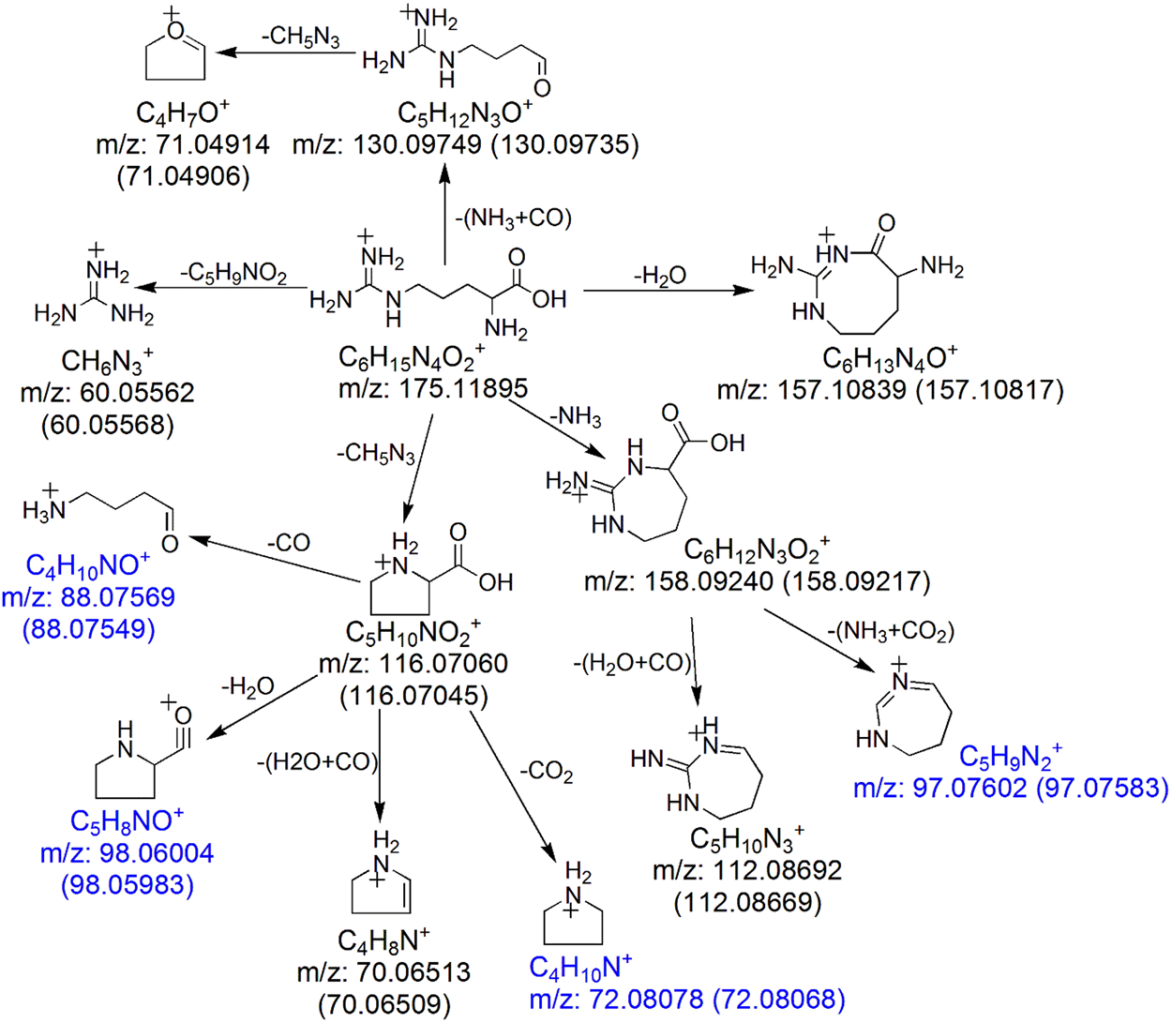
Postulated fragmentation pathways for protonated Arg. The previously unreported fragment ions are shown in blue. The theoretical *m/z* value of each fragment ion is provided under the chemical formula. The observed *m/z* values (mean calculated from 3 replicates) of the fragment ions are provided in the parentheses.

The observed fragment ions of protonated Arg and their chemical identities are summarized in Supplementary Table S1. Energy-resolved fragmentation graph of protonated Arg is provided as Supplementary Fig. S50. Fragmentation of protonated Arg followed two major and three minor pathways (Fig. 5). For the two major pathways, protonated Arg dissociated to form two fragment ions at *m/z* 116.07045 and *m/z* 60.05568 upon the eliminations of HN = C(NH_2_)_2_ (guanidine group, CH_5_N_3_) and C_5_H_9_NO_2_, respectively (Fig. 5a). On one hand, the further loss of H_2_O + CO from the fragment ion at *m/z* 116.07045 resulted in the formation of the predominant fragment ion at *m/z* 70.06509 (Fig. 5b). On the other hand, the losses of CO_2_, CO and H_2_O from the fragment ion at *m/z* 116.07045 led to the formations of three previously unreported fragment ions at *m/z* 72.08068, *m/z* 88.07549 and *m/z* 98.05983, respectively. Their formations were confirmed by observations of the corresponding fragment ions at *m/z* 75.09960 (sequential losses of CD_5_N_3_ and CO_2_), *m/z* 91.09443 (sequential losses of CD_5_N_3_ and CO) and *m/z* 99.06628 (sequential losses of CD_5_N_3_ and D_2_O) when analyzing the fragmentation pattern of [Arg-d_7_ + D]^+^ (Supplementary Fig. S51). For the three minor fragmentation pathways, eliminations of NH_3_, H_2_O and NH_3_ + CO resulted in the formation of three fragment ions at *m/z* 158.09217, *m/z* 157.10817 and *m/z* 130.09735, respectively. A concomitant loss of NH_3_ + CO_2_ from the fragment ion at m/z 158.09217 led to the formation of a previously unreported fragment ion at *m/z* 97.07583. Its formation was confirmed by identifying the corresponding fragment ion at *m/z* 99.08848 (sequential losses of ND_3_ and ND_3_ + CO_2_) in the MS/MS spectra of [Arg-d_7_ + D]^+^ (Supplementary Fig. S51a). A concomitant loss of H_2_O + CO from the fragment ion at *m/z* 158.09217 resulted in the formation of a fragment ion at *m/z* 112.08669. Under high collision energy, the fragment ion at *m/z* 130.09735 dissociated to generate a minor fragment ion at *m/z* 71.04906 upon the loss of the guanidine group (Fig. 5b). Through MS/MS analysis of protonated ^15^Nα-labeled arginine, Shek *et al*. concluded that the loss of NH_3_ from the guanidinium group was involved in the formation of the fragment ions at *m/z* 158 and *m/z* 112, whereas the loss of NH_3_ from the *α-*amino group was involved in the formation of fragment ion at *m/z* 130^22^. Although a fragment ion at *m/z* 97 was observed in their study, it was not annotated. The present study provides the first piece of evidence revealing that this fragment ion was a dissociation product from protonated Arg after the concomitant loss of NH_3_ + CO_2_. Taken together with previous reports^12,22,23^, the postulated fragmentation pathways of protonated Arg are summarized in Fig. 6.

#### Lysine (Lys)

The observed fragment ions of protonated Lys and their chemical identities are summarized in Supplementary Table S1. Energy-resolved fragmentation graph of protonated Lys is provided as Supplementary Fig. S52. MS/MS fragmentation of protonated Lys started from the formation of a fragment ion at *m/z* 130.08608 by the loss of NH_3_, which further dissociated to produce a fragment ion at *m/z* 84.08064 upon the elimination of H_2_O + CO (Supplementary Fig. S53a). A previous investigation of protonated ^15^Nα-labeled Lys revealed that the loss of NH_3_ was exclusively from the amine group at the side chain^12^. Under high collision energy, subsequent losses of C_2_H_4_ and NH_3_ resulted in formation of a fragment ion at *m/z* 56.04967 and a previously unreported fragment ion at *m/z* 67.05425, respectively (Supplementary Fig. S53b). Pseudo MS^3^ analysis of the fragment ion at *m/z* 84.08064 confirmed that it was the precursor of this unreported fragment ion (Supplementary Fig. S54). The annotations of the fragment ions of protonated Lys were further confirmed by MS/MS fragmentation analysis of [Lys-d_5_ + D]^+^ (Supplementary Fig. S55). Taken together with previous investigations^12,13,22^, the postulated fragmentation pathways of protonated Lys are summarized in Supplementary Fig. S56.

#### Ornithine (Orn)

The observed fragment ions of protonated ornithine and their chemical identities are summarized in Supplementary Table S1. Energy-resolved fragmentation graph of protonated ornithine is provided as Supplementary Fig. S57. MS/MS fragmentation of protonated Orn produced three fragment ions at *m/z* 116.07048, *m/z* 115.08651 and *m/z* 70.06511, which were assigned as [M + H−NH_3_]^+^, [M + H−H_2_O]^+^ and [M + H−NH_3_−H_2_O−CO]^+^, respectively (Supplementary Fig. S58). Fragmentation pathways of protonated Orn has not been proposed^7^. Here the fragmentation pathways of protonated Lys are postulated by us and summarized in Supplementary Fig. S59.

#### Cyclic Amino Acid: Proline (Pro)

The fragmentation reaction of protonated Pro was simple. Only one fragment at *m/z* 70.06512 was observed, which was assigned as [M + H−H_2_O−CO]^+^ (Supplementary Fig. S60, Supplementary Fig. S61). The fragmentation pathway of protonated Pro is shown in Supplementary Fig. S62, which is consistent with previous investigations^12,13^.

## Conclusion

To the best of our knowledge, this is the first study in which the fragmentation reactions of protonated AAs were investigated using HR-ESI-MS/MS with CID. Chemical identities were carefully assigned to all the observed fragmentation products. Previously unreported fragment ions were observed for Met, Cys, Gln, Arg and Lys. Isobaric fragment ions of Met and Trp were resolved and assigned with unambiguous chemical identities for the first time. Moreover, the chemical identity of a fragmentation product from protonated Trp that was incorrectly annotated in previous investigations was corrected. All previously unreported fragmentation products and reactions were verified by pseudo MS^3^ experiments and/or MS/MS analyses of deuterated AAs. Clearer pictures of the fragmentation reactions for Met, Cys, Trp, Gln, Arg and Lys were obtained in the present study.

## Methods

### Materials

All 19 AAs were obtained from commercial companies in high purity (Supplementary Table S2). Deuterium oxide ( ≥ 99.96 atom % in D) was obtained from Cambridge Isotope Laboratories (Cambridge, MA). LC-MS grade formic acid (FA), water and acetonitrile (ACN) were obtained from Thermo Fisher Scientific (Waltham, MA). Except L-Tyrosine was prepared in water containing 0.1 M HCl, all the other stock solutions were prepared in MS grade water. Before analysis, individual stock solution was kept frozen at −80 °C. Each AA was diluted to 5 μM working solution using ACN/water (1:1).

### Mass spectrometric analyses−HR-ESI-MS/MS and pseudo MS^3^

HR-ESI-MS/MS fragmentations by HCD, which is a CID technique specific to the orbitrap mass spectrometer, were conducted on a Q Exactive hybrid quadrupole-orbitrap mass spectrometer (Thermo Fisher Scientific) equipped with a heated electrospray source. The MS/MS parameters were set as follow: MS/MS resolution 70,000, AGC 5 × 10^5^, injection time 250 ms, isolation window 0.4 Da.

Using a UHPLC system (Ultimate 3000 RSLC), 10 μL working solution was carried by 50% ACN solution containing 0.1% FA, and infused into the ion source at a flow rate of 0.1 mL/min. The ion source was set as spray voltage, 3.0 kV; sheath gas, 25 (arbitrary unit); Aux gas, off; Aux gas heat, off. In a MS/MS fragmentation experiment, the collision gas was nitrogen, and the collision energy was fixed. Each AA was subjected to six independent fragmentation experiments using different levels of collision energy (NCE 10%, 30%, 40%, 50%, 70% and 90%). For pseudo MS^3^, an in-source fragmentation product from a protonated AA was isolated by the quadrupole (size of isolation window = 0.4 Da). For pseudo MS^3^ analyses of Met, Phe, Tyr, Gln, Glu, an isolated in-source fragmentation product was fragmentated in the collision cell at NCE 30%, whereas NCE 50% was used for pseudo MS^3^ analysis of Lys. All fragmentation experiments were performed in triplicates on different days. Data obtained from the triplicate experiments were used to calculate the mean value of the relative intensity (relative to the total fragment intensity) and *m/z* value of each fragment ion. Chemical identities were assigned to the fragment ions with a maximum mass error tolerance of 5 ppm.

### Production of deuterated AAs

H/D exchange experiment was performed to produce deuterated forms of selected AAs. Briefly, each selected AA was dissolved in MS grade water at a concentration of 1 mg/mL. After vortexing and equilibration, each AA solution was centrifuged at 14,000 g for 10 min to remove any insoluble particles. Before subjected to MS/MS analysis, each AA solution was mixed with D_2_O in a ratio of 1:99. The mixture was equilibrated for 24 hours. Desirable deuterated forms were isolated by quadrupole (size of isolation window = 0.4 Da).

### Infusion MS/MS analysis of deuterated AAs

Two measures were carried out to minimize the back-exchange of H for D in the process of infusion MS/MS analysis of deuterated AAs. First, before using the MS system for infusion MS/MS of a deuterated AA, a blank run of 100% D_2_O was performed in order to replace the residual H^+^ in the MS system with D^+^. Briefly, 100% D_2_O was infused into the electrospray source at a flow rate of 10 μL/min. The analysis time for the blank run was at least 10 minutes. Second, after a 24-hour equilibration, the AA-D_2_O mixture was directly subjected to infusion MS/MS without any additional processing step. This was to minimize the disturbance of the H/D exchange equilibrium. The ion source was set as spray voltage, 3.0 kV; sheath gas, 10 (arbitrary unit); Aux gas, off; Aux gas heat, off. Desirable deuterated forms were isolated by quadrupole (size of isolation window = 0.4 Da).

## Supplementary information

Supplementary Tables and Figures

## Data Availability

All data generated or analyzed during this study are included in this published article and its Supplementary figures and table.
