# Supplementary material for: Revisiting Fragmentation Reactions of Protonated α-Amino Acids by High-Resolution Electrospray Ionization Tandem Mass Spectrometry with Collision-Induced Dissociation
Source: Sci Rep. 2019 Apr 23;9:6453. doi: 10.1038/s41598-019-42777-8 (PMC6478932; doi:10.1038/s41598-019-42777-8)
Supplement: Supplementary file 1 — Supplementary Tables and Figures [file 41598_2019_42777_MOESM1_ESM.pdf]

Revisiting Fragmentation Reactions of Protonated α-Amino Acids by High-Resolution Electrospray Ionization Tandem Mass Spectrometry with Collision-Induced Dissociation

Pengwei ZHANG[1], Wan CHAN[2], Irene L. ANG[1], Rui WEI[1], Melody M.T. LAM[3], Kate M.K. LEI[1], Terence C.W. POON[1]*

[1]Pilot Laboratory, Institute of Translational Medicine, Faculty of Health Sciences, University of Macau, Macau, China;

[2]Department of Chemistry, Hong Kong University of Science and Technology, Hong Kong, China

[3]Proteomics Core, Faculty of Health Sciences, University of Macau, Macau, China

SUPPLEMENTARY INFORMATION – Supplementary Tables and Figures

List of Supplementary Tables and Figures

Supplementary Table S1. Summary of *m/z* values and assigned chemical identities of observed fragment ions from 19 α-amino acids, including 18 proteinogenic amino acids and ornithine.

Supplementary Table S2. Information of the amino acids used in this work.

Supplementary Figure S1. Fragmentation graph of protonated Met under different collision energies.

Supplementary Figure S2. Pseudo MS^3 spectrum of the fragment ion at *m/z* 104.05275 from protonated Met. Two fragment ions at *m/z* 56.04969 and *m/z* 61.01080 were observed.

Supplementary Figure S3. Pseudo MS^3 of the fragment ion at *m/z* 102.05484 from protonated Met. Five fragment ions at *m/z* 85.02835, *m/z* 84.04436, *m/z* 74.05999, *m/z* 74.02361and *m/z* 56.04974 were observed. The two isobaric fragment ions are shown in red, and the underlined one was previously unreported.

Supplementary Figure S4. Representative MS/MS spectra of $[\text{Met-d}_3 + \text{D}]^+$ acquired using collision energy NCE 30% (a) and 70% (b). The two previously unreported fragment ions are shown in blue. The fragment ions corresponding to the two isobaric fragment ions of protonated Met are shown in red, and the underlined one was previously unreported.

Supplementary Figure S5. Fragmentation graph of protonated Cys under different collision energies.

Supplementary Figure S6. Representative MS/MS spectra of protonated Cys acquired using collision energy NCE 30% (a) and 70% (b). The previously unreported fragment ion is shown in blue.

Supplementary Figure S7. Representative MS/MS spectra of $[\text{Cys-d}_4 + \text{D}]^+$ acquired using collision energy NCE 30%. The loss of H_2S from protonated Cys was confirmed by the observation of the loss of D_2S from deuteronated Cys-d_4. The previously unreported fragment ion is shown in blue.

Supplementary Figure S8. Postulated fragmentation pathways of protonated Cys. The previously unreported fragment ion is shown in blue. The theoretical *m/z* value of each fragment ion is provided under the chemical formula.

Supplementary Figure S9. Fragmentation graph of protonated Leu under different collision energies.

Supplementary Figure S10. Representative MS/MS spectra of protonated Leu acquired using collision energy NCE 30% (a) and 70% (b).

Supplementary Figure S11. Fragmentation graph of protonated Ile under different collision energies.

Supplementary Figure S12. Representative MS/MS spectra of protonated Ile acquired using collision energy NCE 30% (a) and 70% (b).

Supplementary Figure S13. Fragmentation graph of protonated Val under different collision energies.

Supplementary Figure S14. Representative MS/MS spectra of protonated Val acquired using collision energy NCE 30% (a) and 70% (b).

Supplementary Figure S15. Postulated fragmentation pathways of protonated Leu (a), protonated Ile (b) and protonated Val (c). The theoretical *m/z* value of each fragment ion is provided under the chemical formula.

Supplementary Figure S16. Fragmentation graph of protonated Trp under different collision energies.

Supplementary Figure S17. Representative MS/MS spectra of $[Trp\text{-}d_4 + D]^+$ acquired using collision energy NCE 30% (a) and 70% (b). The fragment ions corresponding to the two isobaric fragment ions of protonated Trp are shown in red, and the underlined one was previously unreported. The fragment ion corresponding to the fragment ion that was incorrectly annotated in a previous investigation is shown in green

Supplementary Figure S18. Fragmentation graph of protonated Phe under different collision energies.

Supplementary Figure S19. Representative MS/MS spectra of protonated Phe acquired using collision energy NCE 30% (a) and 70% (b).

Supplementary Figure S20. Pseudo MS^3 spectra of fragment ions at *m/z* 120.08061 (a) and *m/z* 131.04895 (b) from protonated Phe. The fragment ion at *m/z* 103.05408 was observed in both spectra.

Supplementary Figure S21. Postulated fragmentation pathways of protonated Phe. The theoretical *m/z* value of each fragment ion is provided under the chemical formula.

Supplementary Figure S22. Fragmentation graph of protonated Tyr under different collision energies

Supplementary Figure S23. Representative MS/MS spectra of protonated Tyr acquired using collision energy NCE 30% (a) and 70% (b).

Supplementary Figure S24. Pseudo MS^3 spectrum of fragment ions at *m/z* 136.07542 (a) and *m/z* 147.04392 (b) from protonated Tyr observed the same fragment at *m/z* 119.04909.

Supplementary Figure S25. Postulated fragmentation pathways of protonated Tyr. The theoretical *m/z* value of each fragment ion is provided under the chemical formula.

Supplementary Figure S26. Fragmentation graph of protonated Gln under different collision energies.

Supplementary Figure S27. Representative MS/MS spectra of protonated Gln acquired using collision energy NCE 30% (a) and 70% (b). The previously unreported fragment ion is shown in blue.

Supplementary Figure S28. Pseudo MS^3 spectrum of fragment ions at *m/z* 130.04968 from protonated Gln. There was no sign of elimination of CO from *m/z* 130.04968.

Supplementary Figure S29. Representative MS/MS spectra of $[Gln\text{-}d_5{+}D]^+$ under collision energy NCE 30%. The previously unreported fragment ion is shown in blue.

Supplementary Figure S30. Postulated fragmentation pathways of protonated Gln. The previously unreported fragment ion is shown in blue. The theoretical *m/z* value of each fragment ion is provided under the chemical formula.

Supplementary Figure S31. Fragmentation graph of protonated Asn under different collision energies.

Supplementary Figure S32 Representative MS/MS spectra of protonated Asn acquired using collision energy NCE 30% (a) and 70% (b).

Supplementary Figure S33. Postulated fragmentation pathways of protonated Asn. The theoretical *m/z* value of each fragment ion is provided under the chemical formula. The theoretical *m/z* value of each fragment ion is provided under the chemical formula.

Supplementary Figure S34. Fragmentation graph of protonated Thr under different collision energies.

Supplementary Figure S35. Representative MS/MS spectra of protonated Thr acquired using collision energy NCE 30% (a) and 70% (b).

Supplementary Figure S36. Postulated fragmentation pathways of protonated Thr. The theoretical *m/z* value of each fragment ion is provided under the chemical formula.

Supplementary Figure S37. Fragmentation graph of protonated Ser under different collision energies.

Supplementary Figure S38. Representative MS/MS spectra of protonated Ser acquired using collision energy NCE 30% (a) and 70% (b).

Supplementary Figure S39. Postulated fragmentation pathways of protonated Ser. The theoretical *m/z* value of each fragment ion is provided under the chemical formula.

Supplementary Figure S40. Fragmentation graph of protonated Glu under different collision energies.

Supplementary Figure S41 Representative MS/MS spectra of protonated Glu acquired using collision energy NCE 30% (a) and 70% (b).

Supplementary Figure S42. Pseudo MS^3 spectra of fragment ions at *m/z* 130.04966 (a) and *m/z* 102.05478 (b) from protonated Glu.

Supplementary Figure S43. Postulated fragmentation pathways of protonated Glu. The theoretical *m/z* value of each fragment ion is provided under the chemical formula.

Supplementary Figure S44. Fragmentation graph of protonated Asp under different collision energies.

Supplementary Figure S45 Representative MS/MS spectra of protonated Asp acquired using collision energy NCE 30% (a) and 70% (b).

Supplementary Figure S46. Postulated fragmentation pathways of protonated Asp. The theoretical *m/z* value of each fragment ion is provided under the chemical formula.

Supplementary Figure S47. Fragmentation graph of protonated His under different collision energies.

Supplementary Figure S48. Representative MS/MS spectra of protonated His acquired using collision energy NCE 30% (a) and 70% (b).

Supplementary Figure S49. Postulated fragmentation pathways of protonated His. The theoretical *m/z* value of each fragment ion is provided under the chemical formula.

Supplementary Figure S50. Fragmentation graph of protonated Arg under different collision energies.

Supplementary Figure S51. Representative MS/MS spectra of $[Arg\text{-}d_7 + D]^+$ acquired using collision energy NCE 30% (a) and 70% (b). The four previously unreported fragments are shown in blue.

Supplementary Figure S52. Fragmentation graph of protonated Lys under different collision energies.

Supplementary Figure S53. Representative MS/MS spectra of protonated Lys acquired using collision energy NCE 30% (a) and 70% (b). A previously unreported fragment ion is shown in blue.

Supplementary Figure S54. Pseudo MS^3 of the fragment ion at *m/z* 84.08064 from protonated Lys. The fragment ion at *m/z* 56.04967 and the previously unreported fragment ion at *m/z* 67.05425 were observed.

Supplementary Figure S55. Representative MS/MS spectra of $[Lys\text{-}d_5 + D]^+$ under collision energy NCE 70%. The previously unreported fragment ion is shown in blue.

Supplementary Figure S56. Postulated fragmentation pathway of protonated Lys. The previously unreported fragment ion is shown in blue. The theoretical *m/z* value of each fragment ion is provided under the chemical formula.

Supplementary Figure S57. Fragmentation graph of protonated ornithine under different collision energies.

Supplementary Figure S58. Representative MS/MS spectra of protonated ornithine acquired using collision energy NCE 30% (a) and 70% (b).

Supplementary Figure S59. Postulated fragmentation pathways of protonated ornithine. The theoretical *m/z* value of each fragment ion is provided under the chemical formula.

Supplementary Figure S60. Fragmentation graph of protonated Pro under different collision energies.

Supplementary Figure S61. Representative MS/MS spectra of protonated Pro acquired using collision energy NCE 30% (a) and 70% (b).

Supplementary Figure S62. Postulated fragmentation pathway of protonated Pro. The theoretical *m/z* value of each fragment ion is provided under the chemical formula.

Supplementary Table S1. Summary of *m/z* values and assigned chemical identities of observed fragment ions from 19 α-amino acids, including 18 proteinogenic amino acids and ornithine.

Protonated amino acid (Theoretical *m/z*)	Observed *m/z* values of fragmentation products (n = 3) [a]		Proposed chemical identity	Number of possible chemical formulae [e]	Chemical formula	Theoretical *m/z*	Mass error (ppm)
	Mean [b, c, d]	Standard Error of the Mean, SEM					
Methionine (150.05833)	133.03155	0.00002	$[M+H-NH_3]^+$	1	$[C_5H_9O_2S]^+$	133.03177	-1.7
	104.05275	0.00005	$[M+H-H_2O-CO]^+$	1	$[C_4H_{10}NS]^+$	104.05285	-0.9
	102.05484	0.00004	$[M+H-CH_3SH]^+$	1	$[C_4H_8NO_2]^+$	102.05495	-1.1
	87.02624	0.00005	$[M+H-NH_3-H_2O-CO]^+$	1	$[C_4H_7S]^+$	87.02630	-0.7
	85.02841	0.00002	$[M+H-CH_3SH-NH_3]^+$	1	$[C_4H_5O_2]^+$	85.02841	0.0
	84.04429	0.00002	$[M+H-CH_3SH-H2O]^+$	1	$[C_4H_6NO]^+$	84.04439	-1.2
	**74.05994**	0.00002	$[M+H-CH_3SH-CO]^+$	1	$[C_3H_8NO]^+$	74.06004	-1.3
	**74.02358**	0.00002	$[M+H-CH_3SH-C_2H_4]^+$	1	$[C_2H_4NO_2]^+$	74.02365	-1.0
	61.01072	0.00002	$[M+H-H_2O-CO-C_2H_5N]^+$	1	$[C_2H_5S]^+$	61.01065	1.2
	56.04969	0.00001	$[M+H-H_2O-CO-CH_3SH]^+$ or $[M+H-CH_3SH-H_2O-CO]^+$	1	$[C_3H_6N]^+$	56.04948	3.8
Cysteine (122.02703)	105.00042	0.00003	$[M+H-NH_3]^+$	1	$[C_3H_5O_2S]^+$	105.00048	-0.6
	88.03923	0.00004	$[M+H-H_2S]^+$	1	$[C_3H_6NO_2]^+$	88.03930	-0.8
	86.98989	0.00003	$[M+H-NH_3-H_2O]^+$	1	$[C_3H_3OS]^+$	86.98991	0.2
	76.02151	0.00002	$[M+H-H_2O-CO]^+$	1	$[C_2H_6NS]^+$	76.02155	-0.5
	58.99525	0.00003	$[M+H-H_2O-CO-NH_3]^+$	1	$[C_2H_3S]^+$	58.99500	4.2
Leucine (132.10191)	86.09628	0.00002	$[M+H-H_2O-CO]^+$	1	$[C_5H_{12}N]^+$	86.09643	-1.7
	69.06988	0.00002	$[M+H-H_2O-CO-NH_3]^+$	1	$[C_5H_9]^+$	69.06988	0.0
Isoleucine (132.10191)	86.09627	0.00002	$[M+H-H_2O-CO]^+$	1	$[C_5H_{12}N]^+$	86.09643	-1.9
	69.06987	0.00002	$[M+H-H_2O-CO-NH_3]^+$	1	$[C_5H_9]^+$	69.06988	-0.1
Valine (118.08626)	72.08053	0.00004	$[M+H-H_2O-CO]^+$	1	$[C_4H_{10}N]^+$	72.08078	-3.5
	55.05446	0.00005	$[M+H-H_2O-CO-NH_3]^+$	1	$[C_4H_7]^+$	55.05423	4.2
Tryptophan (205.09715)	188.07031	0.00005	$[M+H-NH_3]^+$	1	$[C_{11}H_{10}NO_2]^+$	188.07060	-1.6
	170.05975	0.00006	$[M+H-NH_3-H_2O]^+$	1	$[C_{11}H_8NO]^+$	170.06004	-1.7
	159.09145	0.00006	$[M+H-H_2O-CO]^+$	1	$[C_{10}H_{11}N_2]^+$	159.09167	-1.4
	146.05976	0.00011	$[M+H-NH_3-CH_2CO]^+$	1	$[C_9H_8NO]^+$	146.06004	-1.9
	144.08058	0.00009	$[M+H-NH_3-CO_2]^+$	1	$[C_{10}H_{10}N]^+$	144.08078	-1.4

	143.07268	0.00006	$[M+H-NH3-CO_2-\bullet H]^+$	1	$[C_{10}H_9N\bullet]^+$	143.07295	-1.9
	142.06491	0.00008	$[M+H-NH_3-H2O-CO]^+$	1	$[C_{10}H_8N]^+$	142.06513	-1.6
	132.08054	0.00009	$[M+H-H_2O-CO-HCN]^+$	1	$[C_9H_{10}N]^+$	132.08078	-1.8
	130.06489	0.00008	$[M+H-H_2O-CO-HCN-H_2]^+$	1	$[C_9H_8N]^+$	130.06513	-1.8
	118.06499	0.00004	$[M+H-NH_3-CH_2CO-CO]^+$	1	$[C_8H_8N]^+$	118.06513	-1.2
	**117.06970**	0.00008	$[M+H-NH_3-CO_2-HCN]^+$	1	$[C_9H_9]^+$	117.06988	-1.6
	**117.05712**	0.00008	$[M+H-H_2O-CO-HCN-\bullet CH_3]^+$	1	$[C_8H_7N\bullet]^+$	117.05730	-1.5
	115.05404	0.00004	$[M+H-NH_3-H_2O-CO-HCN]^+$	1	$[C_9H_7]^+$	115.05423	-1.6
	103.05410	0.00007	$[M+H-H_2O-CO-HCN-H_2-HCN]^+$	1	$[C_8H_7]^+$	103.05423	-1.2
	91.05406	0.00003	$[M+H-NH_3-CH_2CO-CO-HCN]^+$	1	$[C_7H_7]^+$	91.05423	-1.8
	74.02352	0.00005	$[M+H-C_9H_9N]^+$	1	$[C_2H_4NO_2]^+$	74.02365	-1.8
Phenylalanine (166.08626)	149.05952	0.00006	$[M+H-NH_3]^+$	1	$[C_9H_9O_2]^+$	149.05971	-1.3
	131.04895	0.00005	$[M+H-NH_3-H_2O]^+$	1	$[C_9H_7O]^+$	131.04914	-1.4
	120.08061	0.00003	$[M+H-H_2O-CO]^+$	1	$[C_8H_{10}N]^+$	120.08078	-1.4
	118.06496	0.00005	$[M+H-H_2O-CO-H_2]^+$	1	$[C_8H_8N]^+$	118.06513	-1.4
	107.04899	0.00004	$[M+H-NH_3-CH_2CO]^+$	1	$[C_7H_7O]^+$	107.04914	-1.4
	103.05408	0.00003	$[M+H-NH_3-H_2O-CO]^+$ or $[M+H- H_2O-CO- NH_3]^+$	1	$[C_8H_7]^+$	103.05423	-1.5
	93.06978	0.00004	$[M+H-H_2O-CO-HCN]^+$	1	$[C_7H_9]^+$	93.06988	-1.1
	91.05406	0.00002	$[M+H-H_2O-CO-HCN-H_2]^+$	1	$[C_7H_7]^+$	91.05423	-1.9
	79.05411	0.00002	$[M+H-NH_3-CH_2CO-CO]^+$	1	$[C_6H_7]^+$	79.05423	-1.5
Tyrosine (182.08117)	165.05434	0.00013	$[M+H-NH_3]^+$	1	$[C_9H_9O_3]^+$	165.05462	-1.7
	147.04392	0.00002	$[M+H-NH_3-H_2O]^+$	1	$[C_9H_7O_2]^+$	147.04406	-1.0
	136.07542	0.00018	$[M+H-H_2O-CO]^+$	1	$[C_8H_{10}NO]^+$	136.07569	-2.0
	123.04391	0.00003	$[M+H-NH_3-CH_2CO]^+$	1	$[C_7H_7O_2]^+$	123.04406	-1.2
	119.04909	0.00002	$[M+H-NH_3-H_2O-CO]^+$ or $[M+H- H_2O-CO- NH_3]^+$	1	$[C_8H_7O]^+$	119.04914	-0.4
	107.04904	0.00002	$[M+H-H_2O-CO-HCN-H_2]^+$	1	$[C_7H_7O]^+$	107.04914	-0.9
	95.04904	0.00003	$[M+H-NH_3-CH_2CO-CO]^+$	1	$[C_6H_7O]^+$	95.04914	-1.1
	91.05411	0.00002	$[M+H-NH_3-H_2O-CO-CO]^+$	1	$[C_7H_7]^+$	91.05423	-1.3
	65.03868	0.00002	$[M+H-NH_3-CH_2CO-CO-CH_2O]^+$	1	$[C_5H_5]^+$	65.03858	1.5
Glutamine (147.07642)	130.04968	0.00007	$[M+H-NH_3]^+$	1	$[C_5H_8NO_3]^+$	130.04987	-1.4
	102.05480	0.00006	$[M+H-NH_3-CO]^+$	1	$[C_4H_8NO_2]^+$	102.05495	-1.5

	101.07084	0.00004	$[M+H-H_2O-CO]^+$	1	$[C_4H_9N_2O]^+$	101.07094	-1.0
	84.04428	0.00006	$[M+H-NH_3-H_2O-CO]^+$	1	$[C_4H_6NO]^+$	84.04439	-1.3
	56.04972	0.00004	$[M+H-NH_3-H_2O-CO-CO]^+$	1	$[C_3H_6N]^+$	56.04948	4.4
Asparagine (133.06034)	116.03411	0.00013	$[M+H-NH_3]^+$	1	$[C_4H_6NO_3]^+$	116.03422	-1.0
	88.03928	0.00012	$[M+H-NH_3-CO]^+$	1	$[C_3H_6NO_2]^+$	88.03930	-0.2
	87.05521	0.00009	$[M+H-H_2O-CO]^+$	1	$[C_3H_7N_2O]^+$	87.05529	-0.9
	74.02359	0.00009	$[M+H-NH_3-CH_2CO]^+$	1	$[C_2H_4NO_2]^+$	74.02365	-0.8
	70.02874	0.00007	$[M+H-H_2O-CO-NH_3]^+$	1	$[C_3H_4NO]^+$	70.02874	0.0
Threonine (120.06552)	102.05502	0.00013	$[M+H-H_2O]^+$	1	$[C_4H_8NO_2]^+$	102.05495	0.7
	84.04431	0.00007	$[M+H-2H_2O]^+$	1	$[C_4H_6NO]^+$	84.04439	-0.9
	75.04401	0.00009	$[M+H-NH_3-CO]^+$	1	$[C_3H_7O_2]^+$	75.04406	-0.7
	74.05993	0.00006	$[M+H-H_2O-CO]^+$	1	$[C_3H_8NO]^+$	74.06004	-1.5
	56.04971	0.00004	$[M+H-H_2O-CO-H_2O]^+$	1	$[C_3H_6N]^+$	56.04948	4.2
Serine (106.04987)	88.03917	0.00007	$[M+H-H_2O]^+$	1	$[C_3H_6NO_2]^+$	88.03930	-1.4
	70.02870	0.00006	$[M+H-2H_2O]^+$	1	$[C_3H_4NO]^+$	70.02874	-0.6
	60.04457	0.00004	$[M+H-H_2O-CO]^+$	1	$[C_2H_6NO]^+$	60.04439	2.9
Glutamic acid (148.06043)	130.04966	0.00009	$[M+H-H_2O]^+$	1	$[C_5H_8NO_3]^+$	130.04987	-1.6
	102.05478	0.00007	$[M+H-H_2O-CO]^+$	1	$[C_4H_8NO_2]^+$	102.05495	-1.7
	84.04427	0.00006	$[M+H-H_2O-H_2O-CO]^+$ or $[M+H-H_2O-CO-H_2O]^+$	1	$[C_4H_6NO]^+$	84.04439	-1.4
	56.04970	0.00003	$[M+H-H_2O-H_2O-CO-CO]^+$	1	$[C_3H_6N]^+$	56.04948	3.9
Aspartic acid (133.04478)	116.03401	0.00011	$[M+H-H_2O]^+$	1	$[C_4H_6NO_3]^+$	116.03422	-1.1
	88.03914	0.00008	$[M+H-H_2O-CO]^+$	1	$[C_3H_6NO_2]^+$	88.03930	-1.9
	74.02362	0.00005	$[M+H-H_2O-CH_2CO]^+$	1	$[C_2H_4NO_2]^+$	74.02365	-0.5
Histidine (156.07676)	110.07114	0.00006	$[M+H-H_2O-CO]^+$	1	$[C_5H_8N_3]^+$	110.07127	-1.2
	95.06013	0.00005	$[M+H-NH_3-CO_2]^+$	1	$[C_5H_7N_2]^+$	95.06037	-2.5
	93.04460	0.00005	$[M+H-H_2O-CO-NH_3]^+$	1	$[C_5H_5N_2]^+$	93.04472	-1.3
	83.06021	0.00005	$[M+H-H_2O-CO-HCN]^+$	1	$[C_4H_7N_2]^+$	83.06037	-1.9
	82.05242	0.00004	$[M+H-H_2O-CO-HCN-\bullet H]^+$	1	$[C_4H_6N_2\bullet]^+$	82.05255	-1.6
	81.04459	0.00006	$[M+H-H_2O-CO-HCN-H_2]^+$	1	$[C_4H_5N_2]^+$	81.04472	-1.6
	68.04949	0.00003	$[M+H-NH_3-CO_2-HCN]^+$	1	$[C_4H_6N]^+$	68.04948	0.1
	56.04971	0.00003	$[M+H-H_2O-CO-HCN-HCN]^+$	1	$[C_3H_6N]^+$	56.04948	4.0
Arginine	158.09217	0.00006	$[M+H-NH_3]^+$	1	$[C_6H_{12}N_3O_2]^+$	158.09240	-1.5

(175.11895)	157.10817	0.00005	$[M+H-H_2O]^+$	1	$[C_6H_{13}N_4O]^+$	157.10839	-1.4
	130.09735	0.00006	$[M+H-NH_3-CO]^+$	1	$[C_5H_{12}N_3O]^+$	130.09749	-1.1
	116.07045	0.00004	$[M+H-CH_5N_3]^+$	1	$[C_5H_{10}NO_2]^+$	116.07060	-1.3
	112.08669	0.00002	$[M+H-NH_3-H_2O-CO]^+$	1	$[C_5H_{10}N_3]^+$	112.08692	-2.0
	98.05983	0.00001	$[M+H-CH_5N_3-H_2O]^+$	1	$[C_5H_8NO]^+$	98.06004	-2.1
	97.07583	0.00001	$[M+H-NH_3-NH_3-CO_2]^+$	1	$[C_5H_9N_2]^+$	97.07602	-2.0
	88.07549	0.00001	$[M+H-CH_5N_3-CO]^+$	1	$[C_4H_{10}NO]^+$	88.07569	-2.3
	72.08068	0.00002	$[M+H-CH_5N_3-CO2]^+$	1	$[C_4H_{10}N]^+$	72.08078	-1.4
	71.04906	0.00006	$[M+H-NH_3-CO-CH_5N_3]^+$	1	$[C_4H_7O]^+$	71.04914	-1.2
	70.06509	0.00007	$[M+H-CH_5N_3-H_2O-CO]^+$	1	$[C_4H_8N]^+$	70.06513	-0.6
	60.05568	0.00005	$[M+H-C_5H_9NO_2]^+$	1	$[C_4H_6N_3]^+$	60.05562	1.1
Lysine (147.11280)	130.08608	0.00009	$[M+H-NH_3]^+$	1	$[C_6H_{12}NO_2]^+$	130.08626	-1.4
	84.08064	0.00005	$[M+H-NH_3-H_2O-CO]^+$	1	$[C_5H_{10}N]^+$	84.08078	-1.7
	67.05425	0.00005	$[M+H-NH_3-H_2O-CO-NH_3]^+$	1	$[C_5H_7]^+$	67.05423	0.3
	56.04967	0.00003	$[M+H-NH_3-H_2O-CO-C_2H_4]^+$	1	$[C_3H_6N]^+$	56.04948	3.3
Ornithine (133.09715)	116.07048	0.00003	$[M+H-NH_3]^+$	1	$[C_5H_{10}NO_2]^+$	116.07060	-1.0
	115.08651	0.00003	$[M+H-H_2O]^+$	1	$[C_5H_{11}N_2O]^+$	115.08659	-0.7
	70.06511	0.00002	$[M+H-NH_3-H_2O-CO]^+$	1	$[C_4H_8N]^+$	70.06513	-0.2
Proline (116.07060)	70.06512	0.00002	$[M+H-H_2O-CO]^+$	1	$[C_4H_8N]^+$	70.06513	-0.1

[a] The mean *m/z* value was calculated using the data from three independent experiments performed on different days.

[b] Previously unreported fragment ions are in italics.

[c] Isobaric fragment ions are shown in bold, and the underlined one was previously unreported.

[d] The Fragment ion that was incorrectly annotated in the previous investigations is shown in green.

[e] The chemical formula of each fragment was calculated using the observed mean *m/z* ± 5 ppm with the element constraints: N: 0-10, O: 0-15, C: 1-30, H: 0-60. For Met and cysteine, sulfur atom (S: 0-10) was also included for chemical formula calculation. The chemical formula calculation was performed using the tool embedded in Thermo Qual Browser (Thermo Scientific Xcalibur v 3.0).

Supplementary Table S2. Information of the amino acids used in this work.

Amino acid standard	Form	Commercial provider	Cat. No.	Purity	Stock concentration (mM)
L-Arginine	Neutral	Sigma-Aldrich, USA	PHR1106	99.6%	11
L-Asparagine	Neutral	Sigma-Aldrich, SWITZERLAND	51363	99.6%	4
L-Aspartic acid	Neutral	Sigma-Aldrich, USA	PHR1104	99.6%	2
L-Cysteine	Neutral	Sigma-Aldrich, USA	95437	98.4%	5
L-Glutamic acid	Neutral	Sigma-Aldrich, USA	PHR1107	99.7%	8.5
L-Glutamine	Neutral	Sigma-Aldrich, USA	PHR1125	100.1%	59
L-Histidine	Neutral	Sigma-Aldrich, USA	PHR1108	99.9%	8
L-Isoleucine	Neutral	Sigma-Aldrich, USA	PHR1099	98.9%	6
L-Leucine	Neutral	Sigma-Aldrich, USA	PHR1105	99.0%	13
L-Lysine	Neutral	Sigma-Aldrich, SWITZERLAND	23128	100.0%	18
L-Methionine	Neutral	Sigma-Aldrich, USA	M9625	99.0%	3
L-Ornithine	Monohydrochloride	Sigma-Aldrich, USA	57197	100.0%	5.5
L-Phenylalanine	Neutral	Sigma-Aldrich, USA	PHR1100	99.9%	6.5
L-Proline	Neutral	Sigma-Aldrich, USA	PHR1332	100.0%	24
L-Serine	Neutral	Sigma-Aldrich, USA	PHR1103	99.9%	14
L-Threonine	Neutral	Sigma-Aldrich, USA	PHR1242	100.0%	14
L-Tryptophan	Neutral	Sigma-Aldrich, USA	PHR1176	100.0%	5
L-Tyrosine	Neutral	Sigma-Aldrich, USA	PHR1097	99.6%	10
L-Valine	Neutral	Sigma-Aldrich, USA	PHR1172	98.9%	23

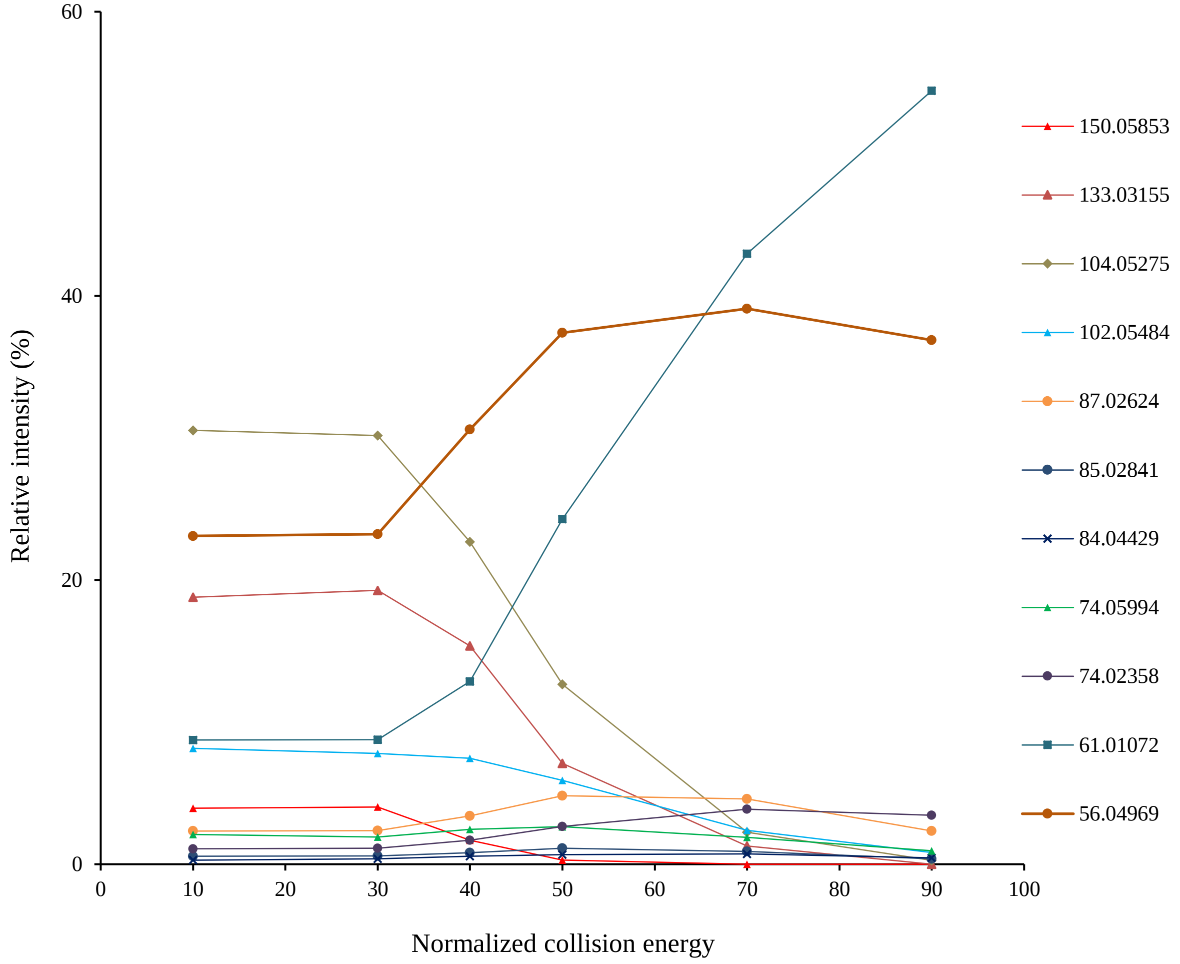

Supplementary Figure S1. Fragmentation graph of protonated Met under different collision energies.

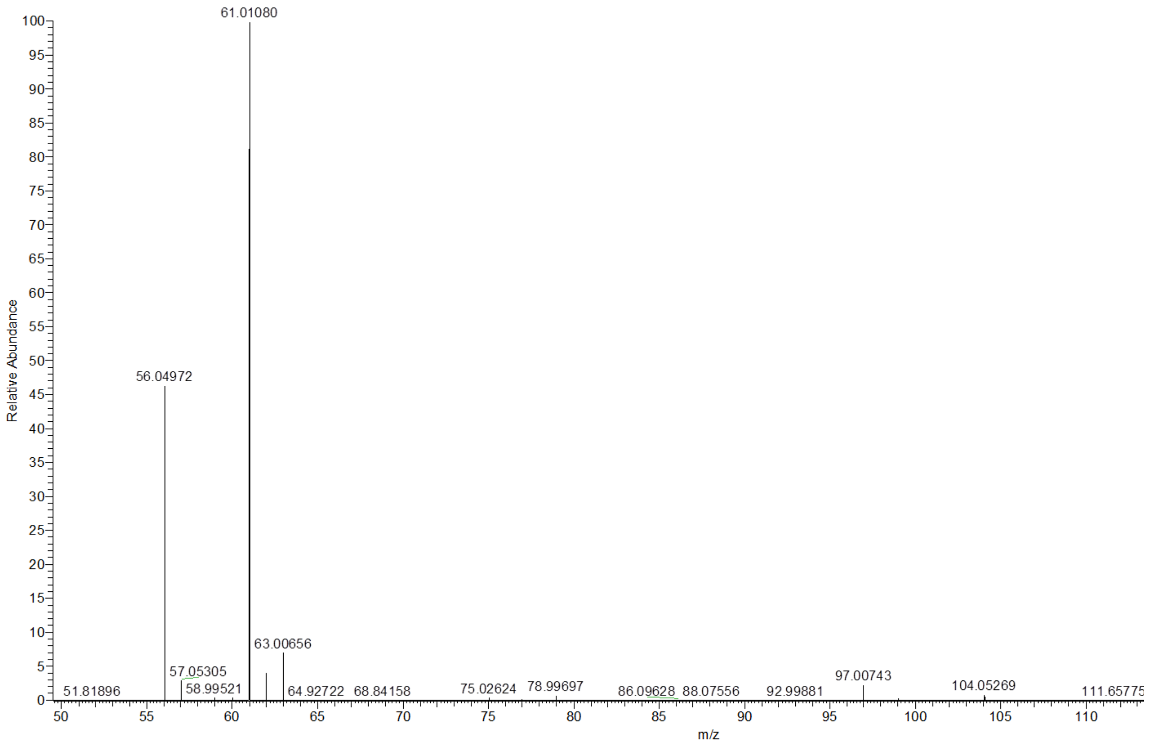

Supplementary Figure S2. Pseudo MS^3 spectrum of the fragment ion at *m/z* 104.05275 from protonated Met. Two fragment ions at *m/z* 56.04969 and *m/z* 61.01080 were observed.

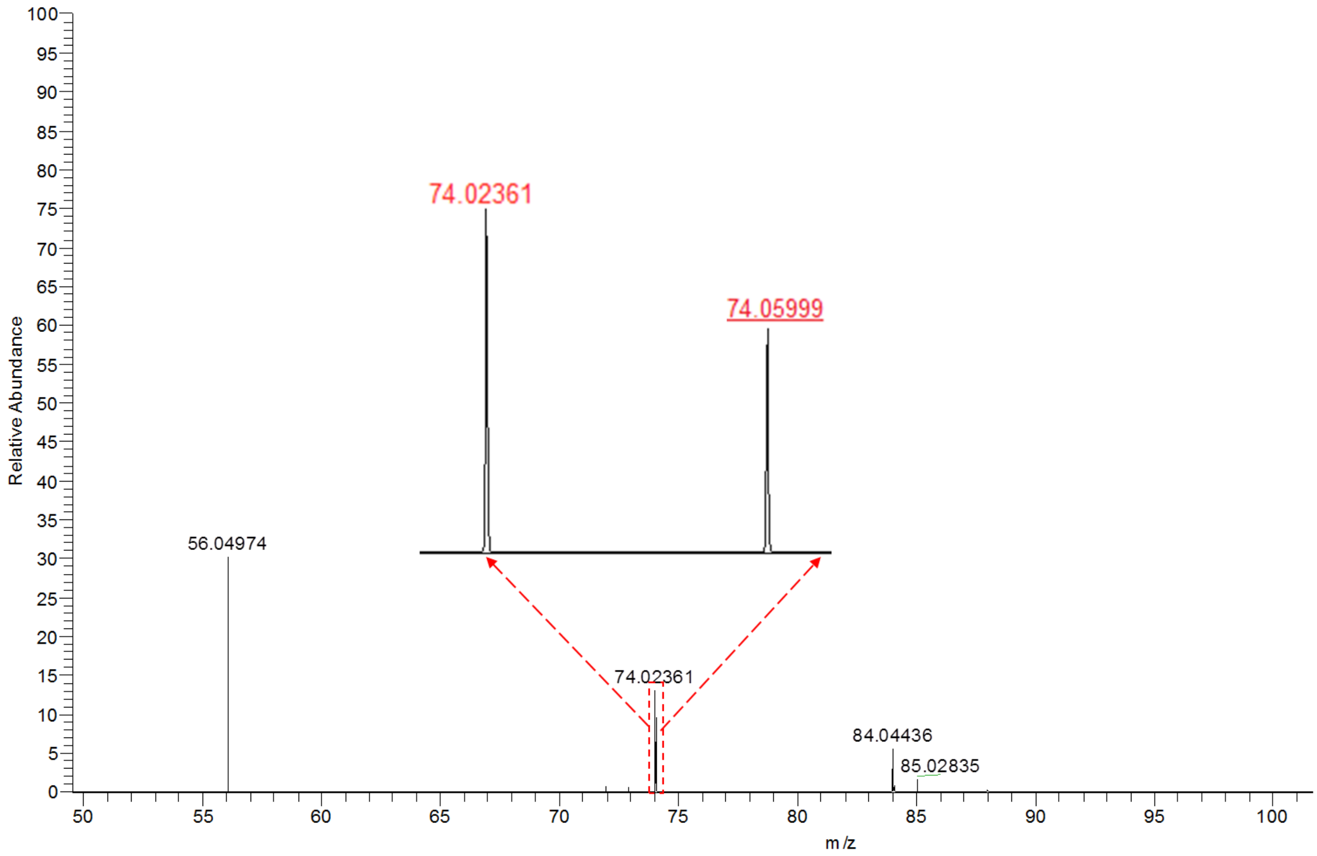

Supplementary Figure S3. Pseudo MS^3 of the fragment ion at *m/z* 102.05484 from protonated Met. Five fragment ions at *m/z* 85.02835, *m/z* 84.04436, *m/z* 74.05999, *m/z* 74.02361and *m/z* 56.04974 were observed. The two isobaric fragment ions are shown in red, and the underlined one was previously unreported.

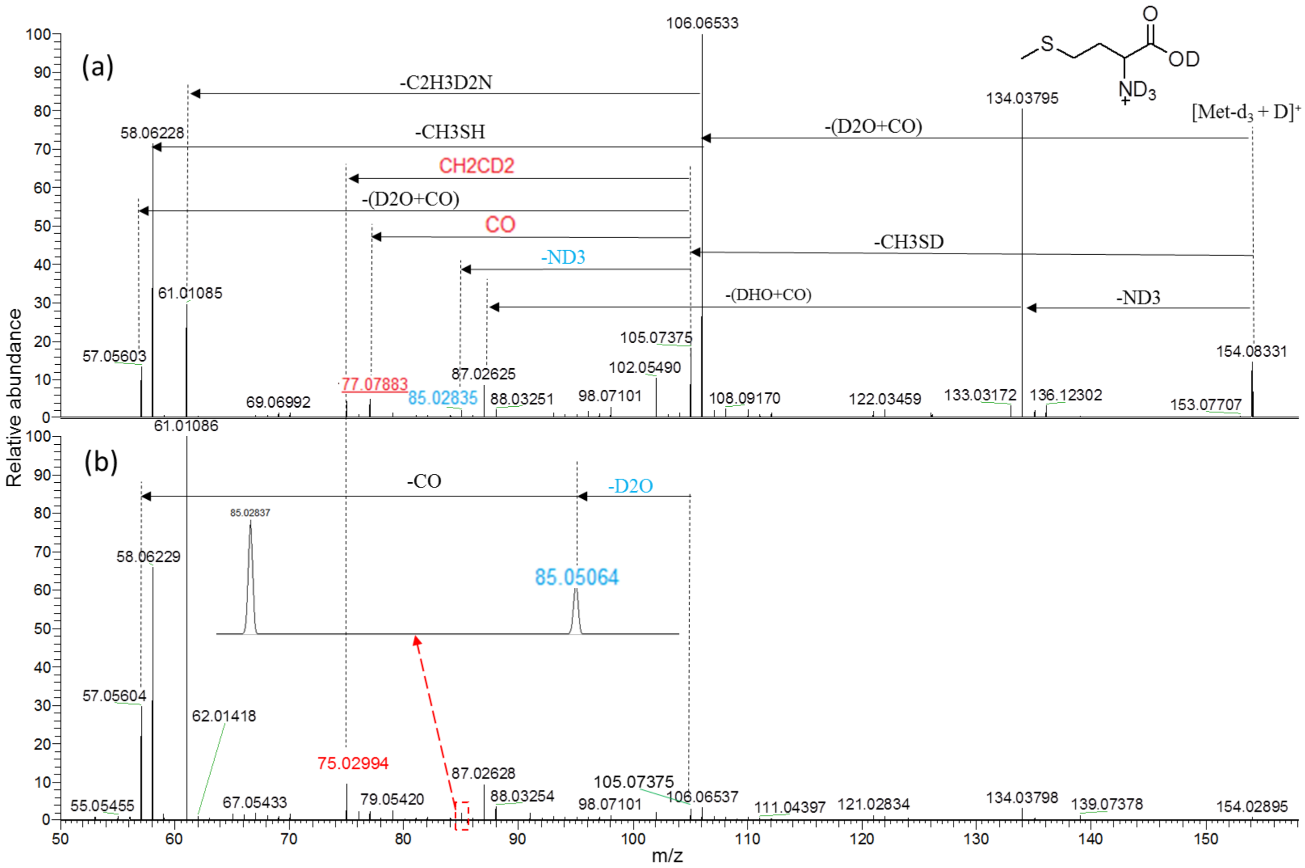

Supplementary Figure S4. Representative MS/MS spectra of [Met-d_3 + D]$^+$ acquired using collision energy NCE 30% (a) and 70% (b). The two previously unreported fragment ions are shown in blue. The fragment ions corresponding to the two isobaric fragment ions of protonated Met are shown in red, and the underlined one was previously unreported.

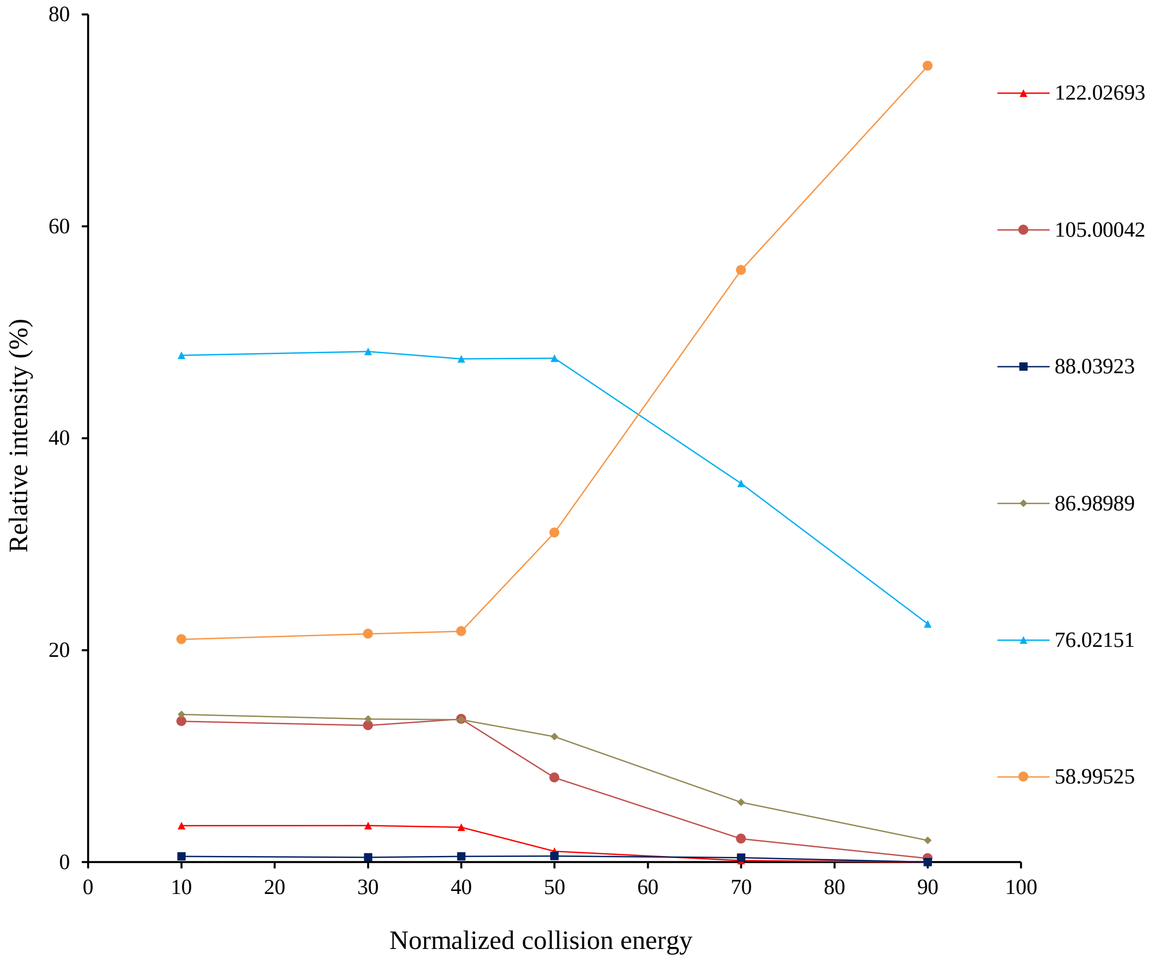

Supplementary Figure S5. Fragmentation graph of protonated Cys under different collision energies.

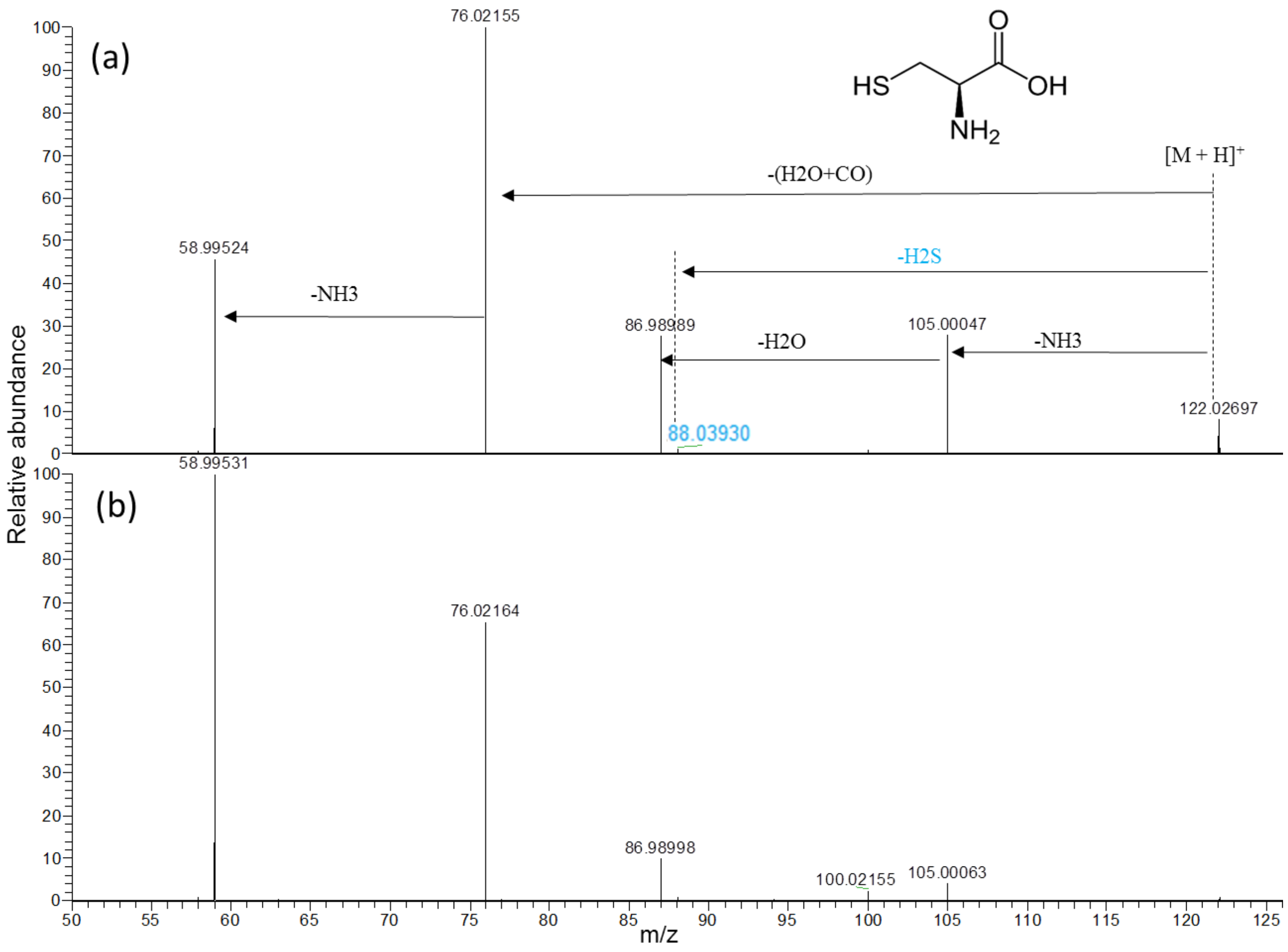

Supplementary Figure S6. Representative MS/MS spectra of protonated Cys acquired using collision energy NCE 30% (a) and 70% (b). The previously unreported fragment ion is shown in blue.

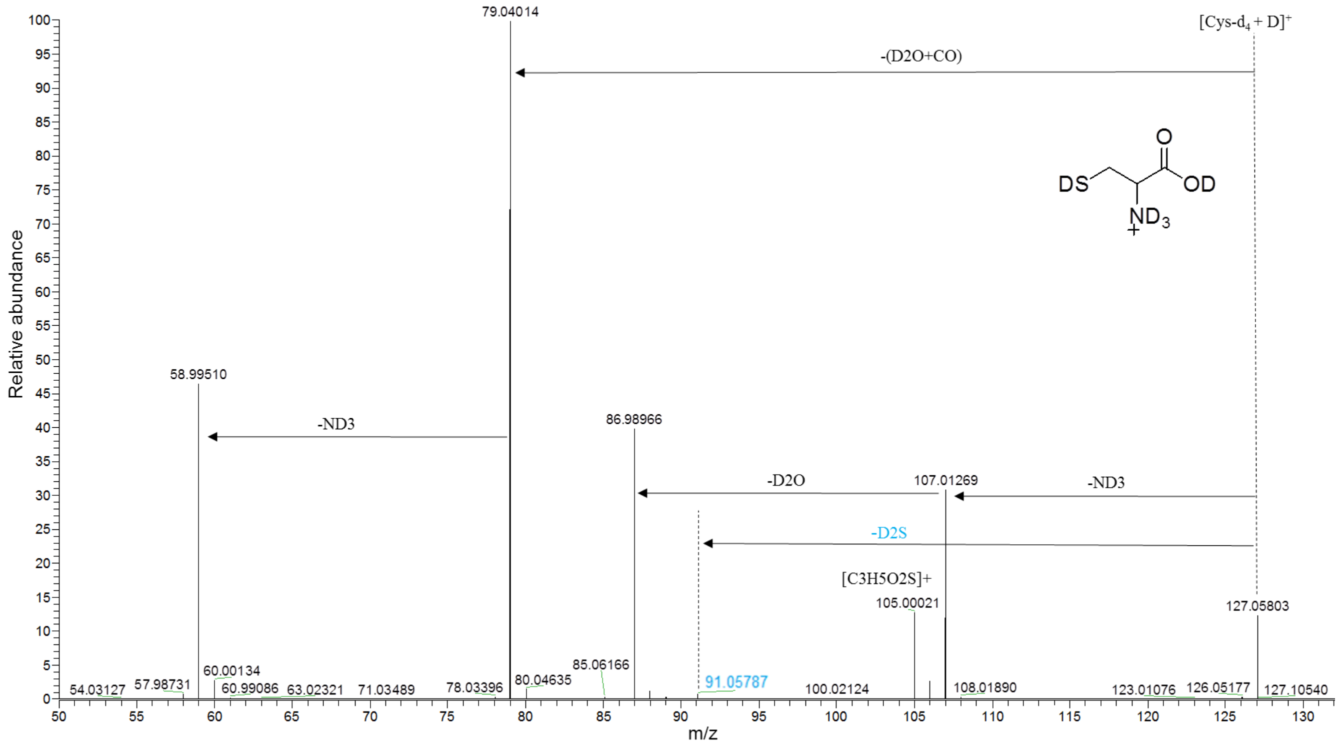

Supplementary Figure S7. Representative MS/MS spectra of [Cys-d_4 + D]$^+$ acquired using collision energy NCE 30%. The loss of H_2S from protonated Cys was confirmed by the observation of the loss of D_2S from deuteronated Cys-d_4. The previously unreported fragment ion is shown in blue.

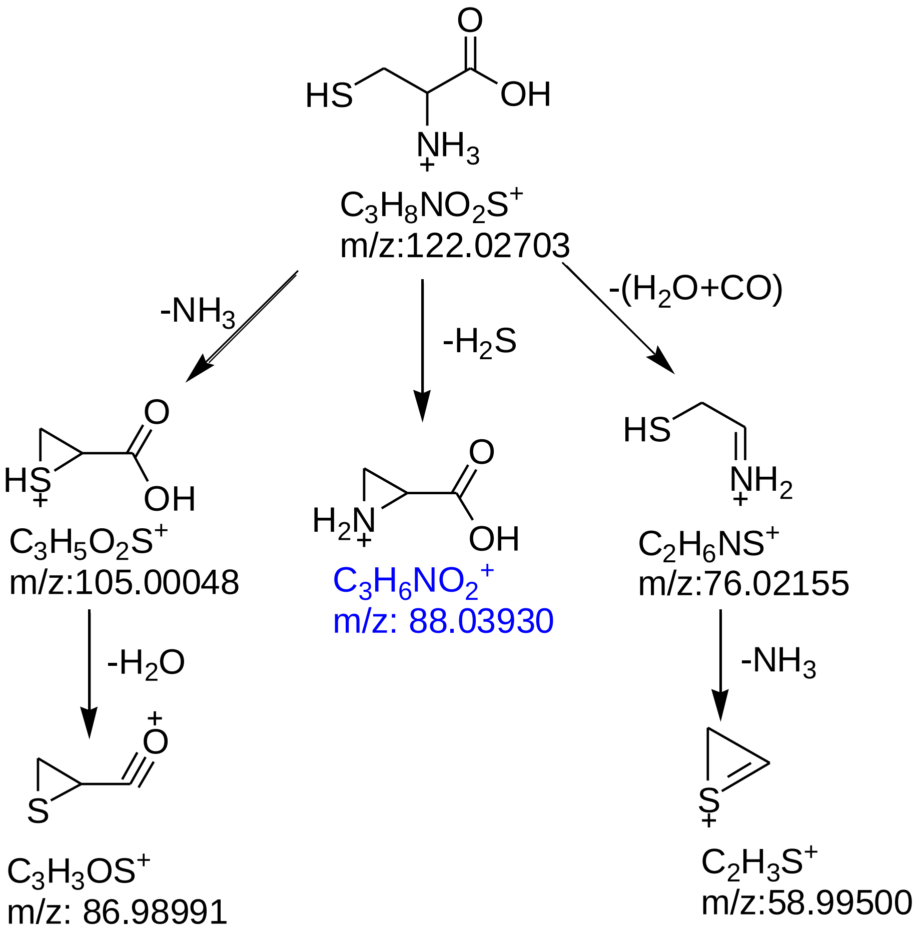

Supplementary Figure S8. Postulated fragmentation pathways of protonated Cys. The previously unreported fragment ion is shown in blue. The theoretical *m/z* value of each fragment ion is provided under the chemical formula.

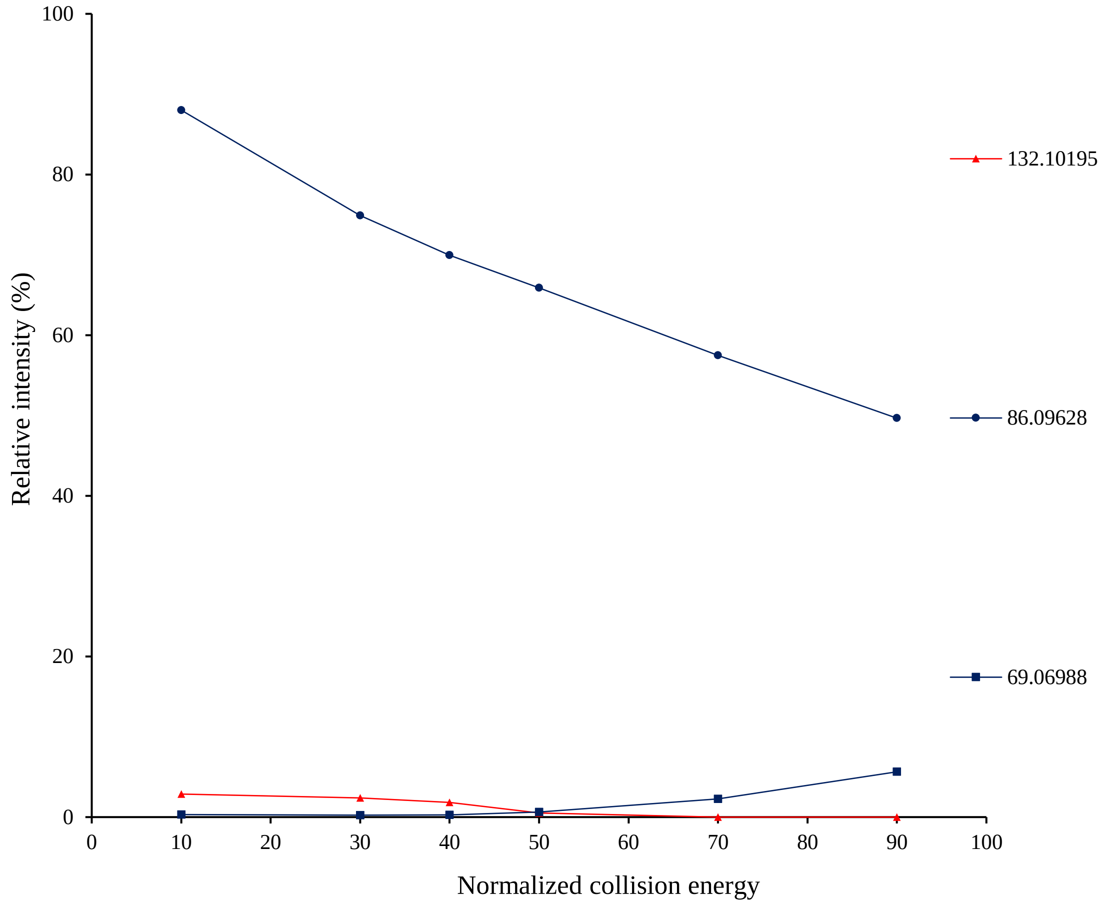

Supplementary Figure S9. Fragmentation graph of protonated Leu under different collision energies.

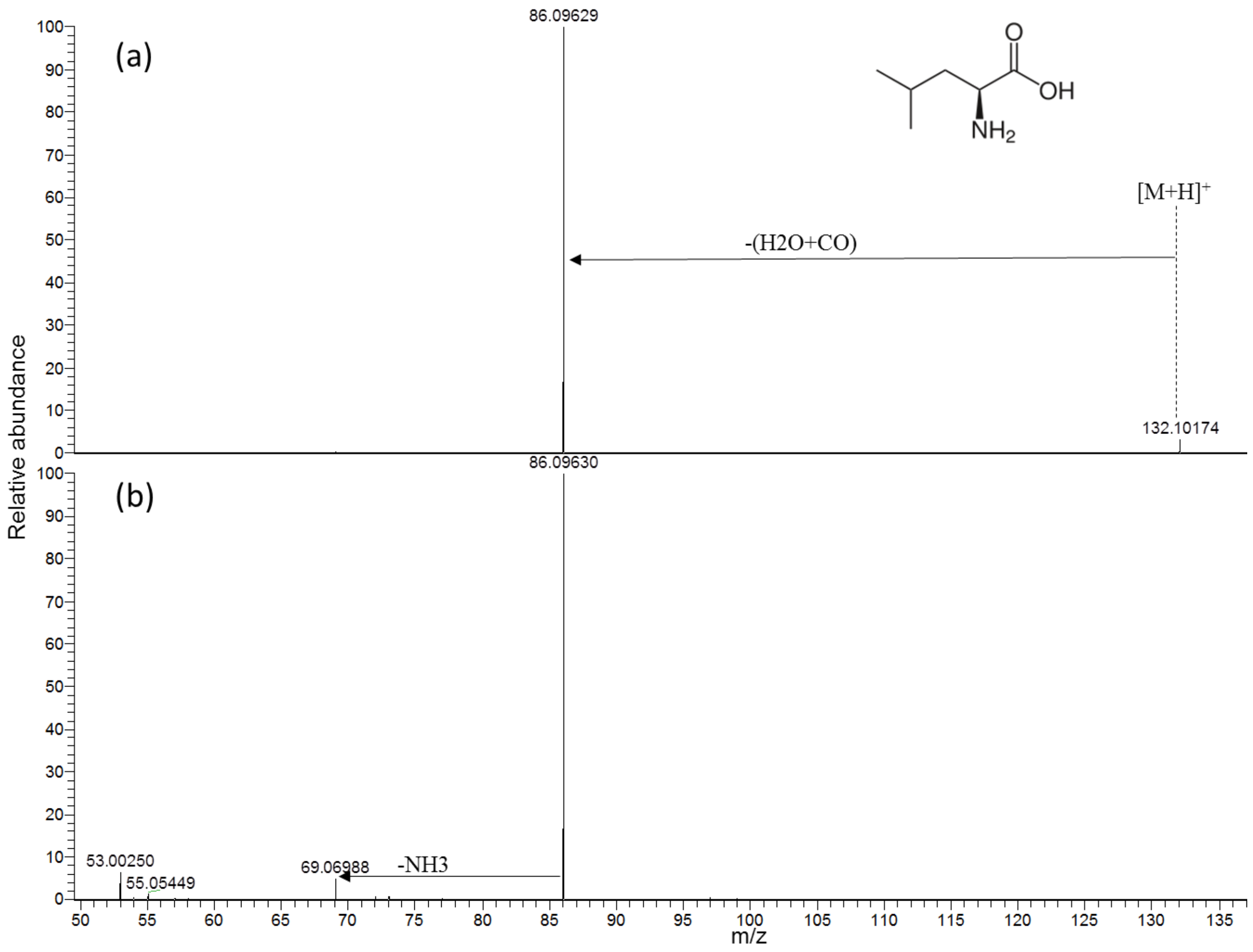

Supplementary Figure S10. Representative MS/MS spectra of protonated Leu acquired using collision energy NCE 30% (a) and 70% (b).

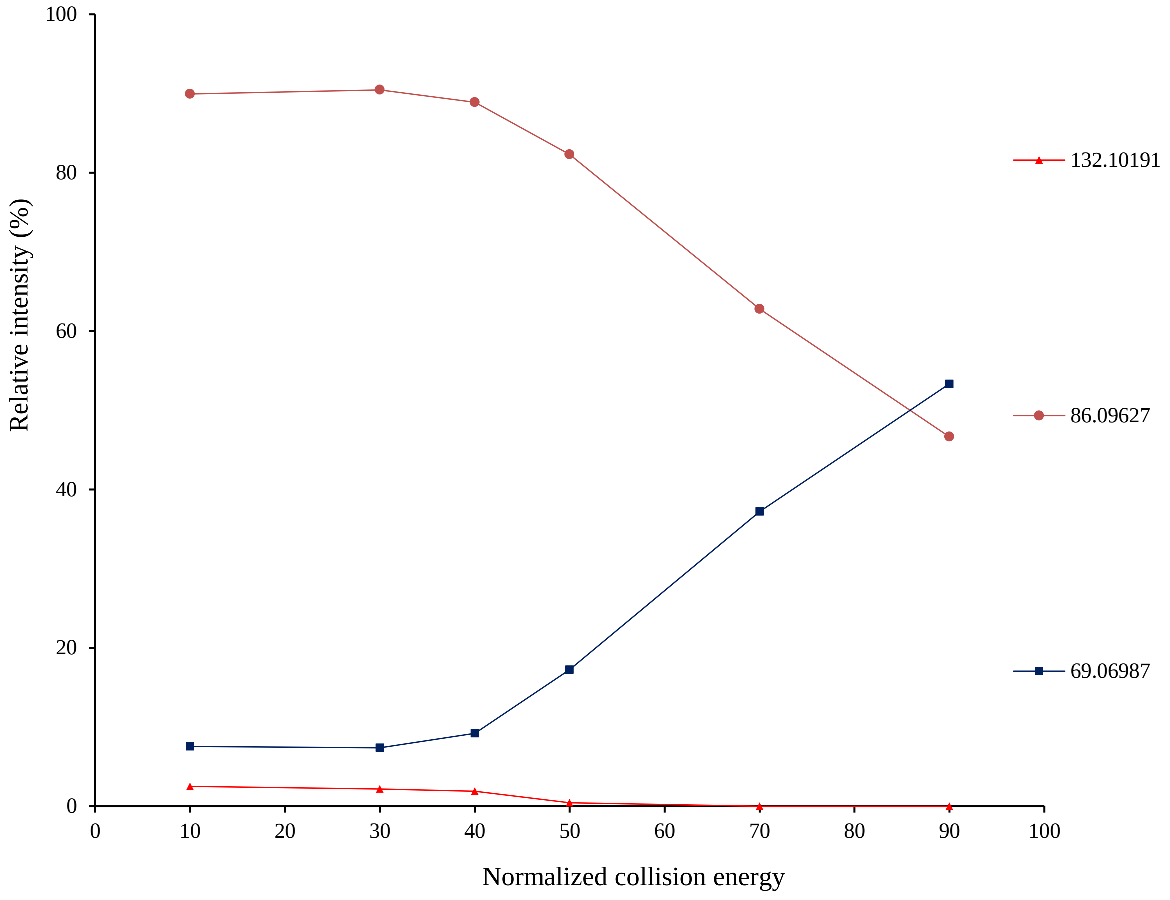

Supplementary Figure S11. Fragmentation graph of protonated Ile under different collision energies.

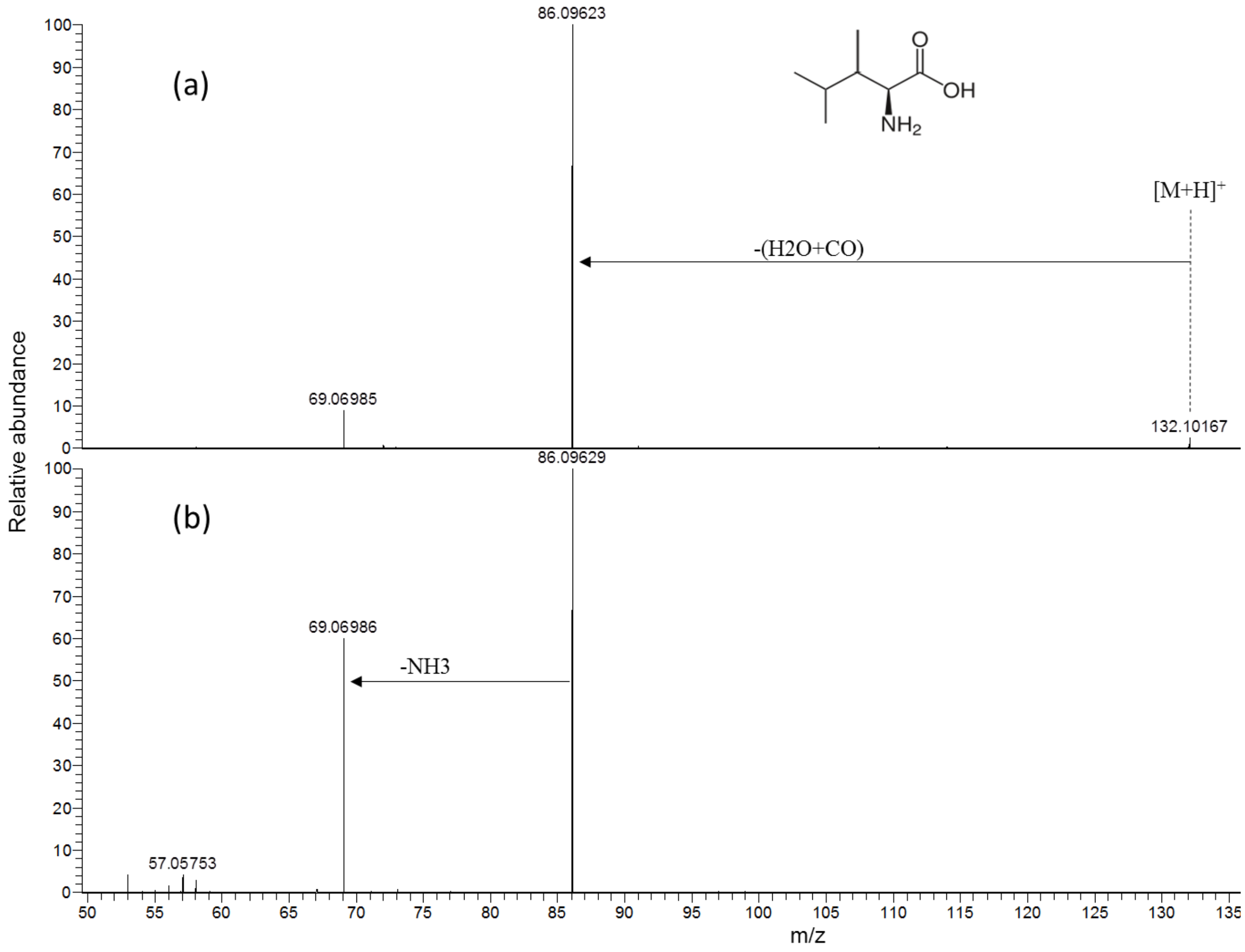

Supplementary Figure S12. Representative MS/MS spectra of protonated Ile acquired using collision energy NCE 30% (a) and 70% (b).

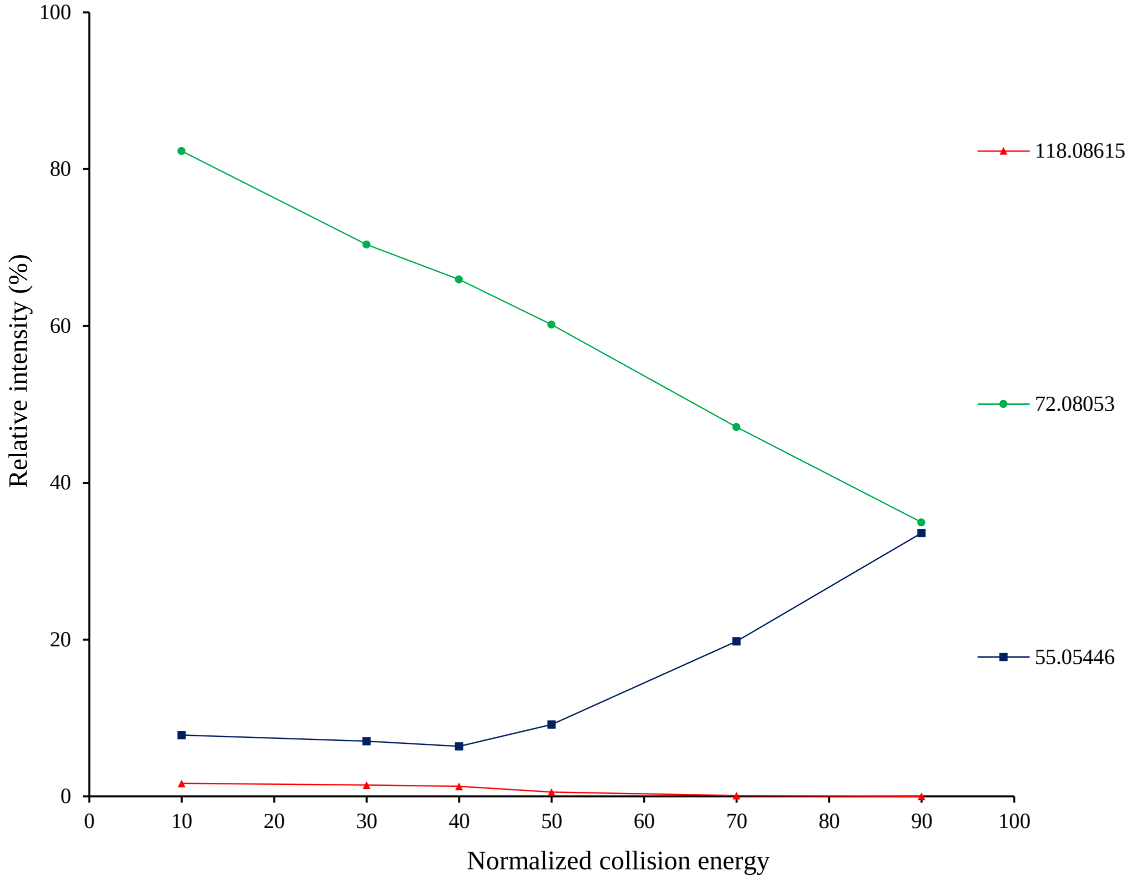

Supplementary Figure S13. Fragmentation graph of protonated Val under different collision energies.

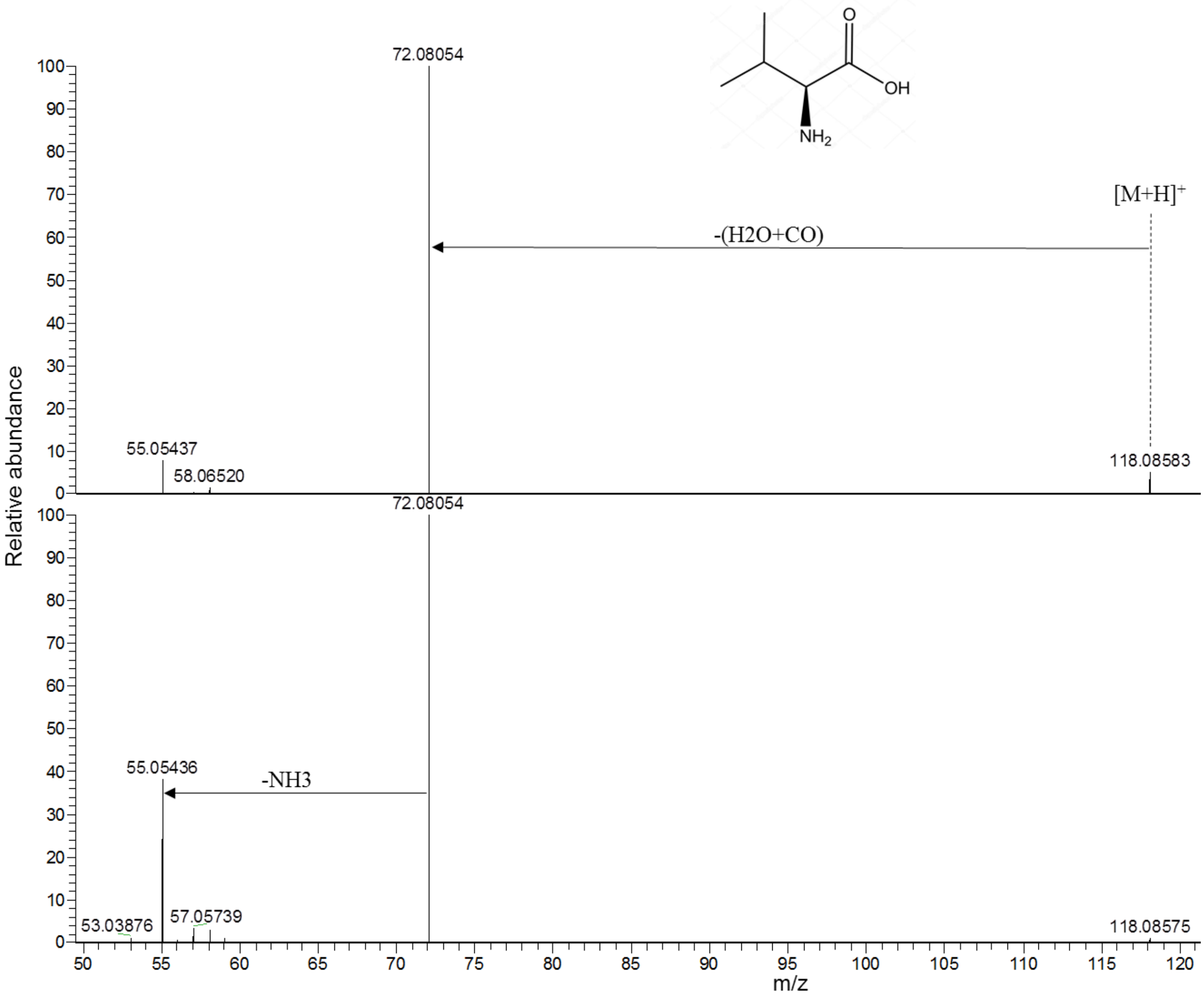

Supplementary Figure S14. Representative MS/MS spectra of protonated Val acquired using collision energy NCE 30% (a) and 70% (b).

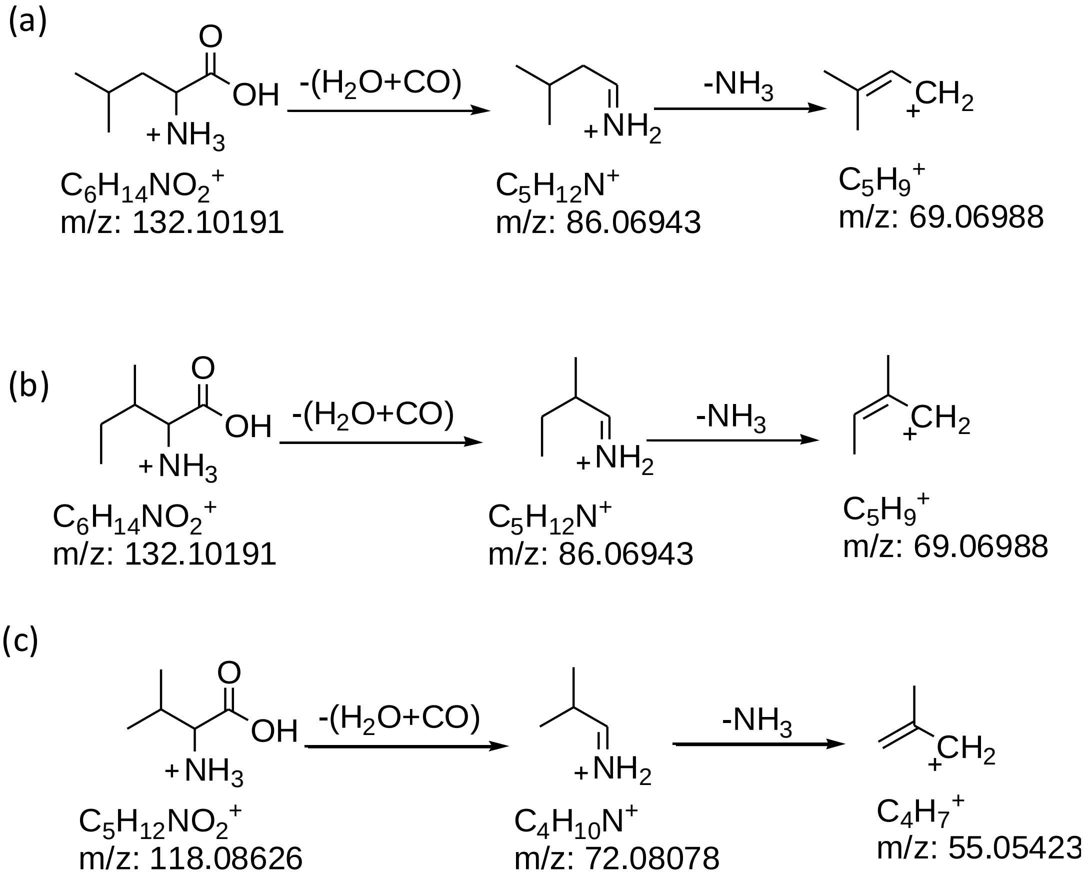

Supplementary Figure S15. Postulated fragmentation pathways of protonated Leu (a), protonated Ile (b) and protonated Val (c). The theoretical *m/z* value of each fragment ion is provided under the chemical formula.

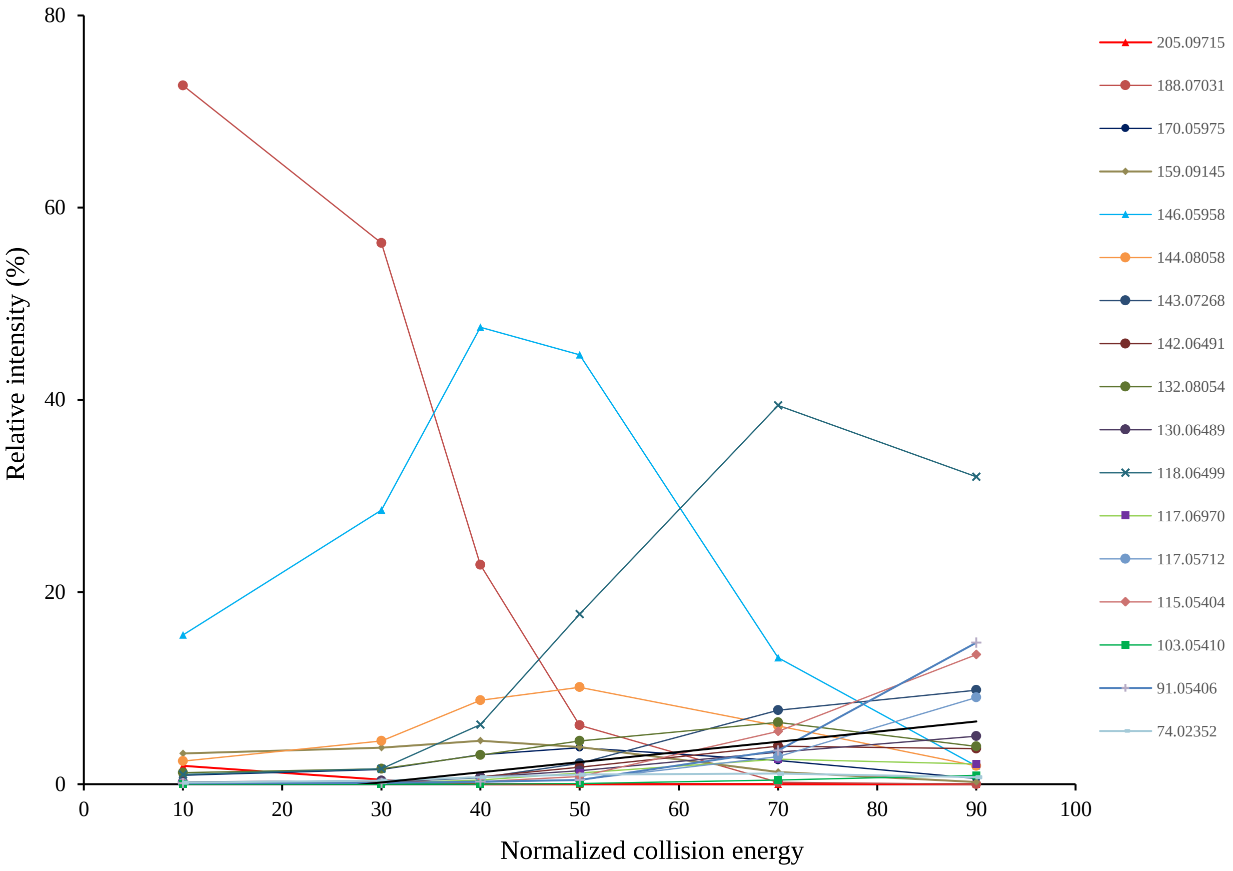

Supplementary Figure S16. Fragmentation graph of protonated Trp under different collision energies.

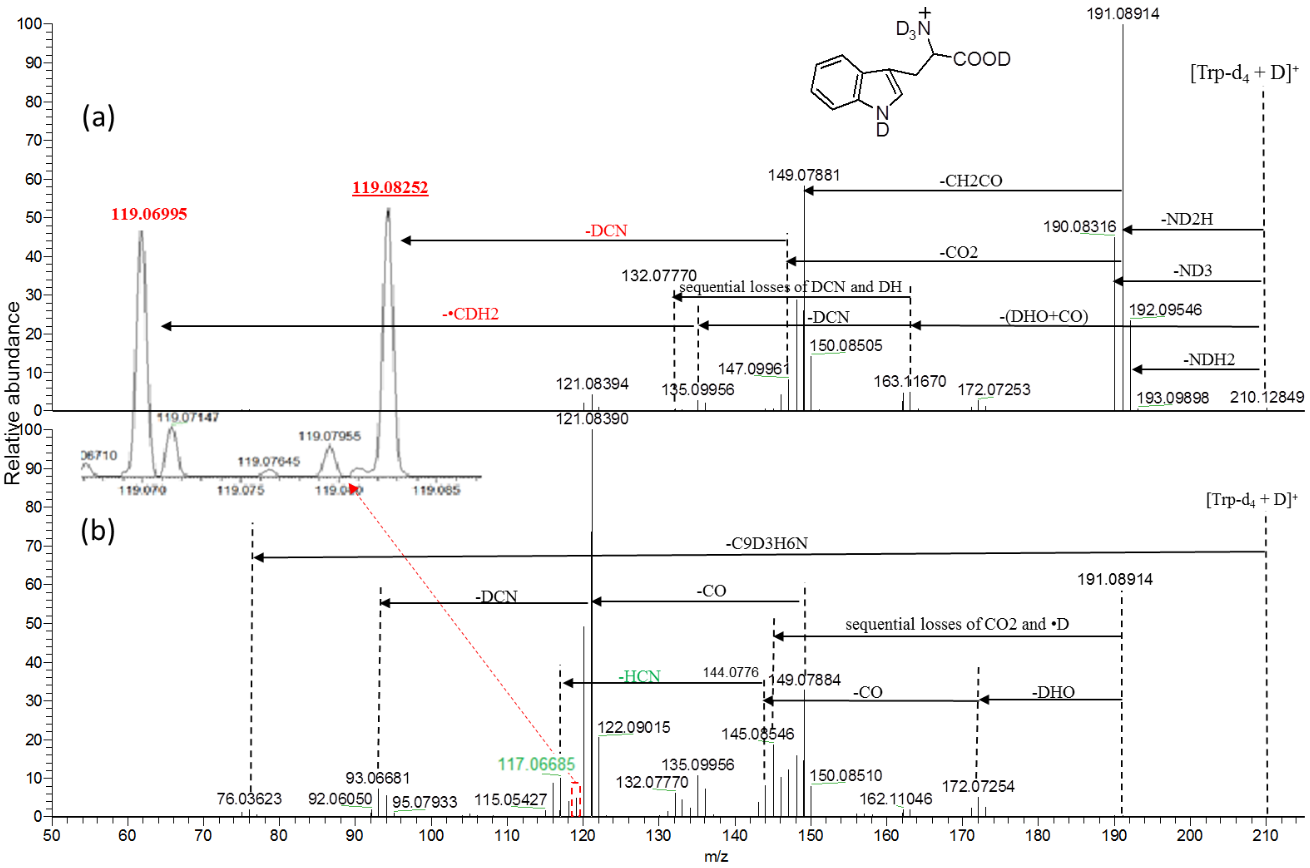

Supplementary Figure S17. Representative MS/MS spectra of [Trp-d$_4$ + D]$^+$ acquired using collision energy NCE 30% (a) and 70% (b). The fragment ions corresponding to the two isobaric fragment ions of protonated Trp are shown in red, and the underlined one was previously unreported. The fragment ion corresponding to the fragment ion that was incorrectly annotated in a previous investigation is shown in green.

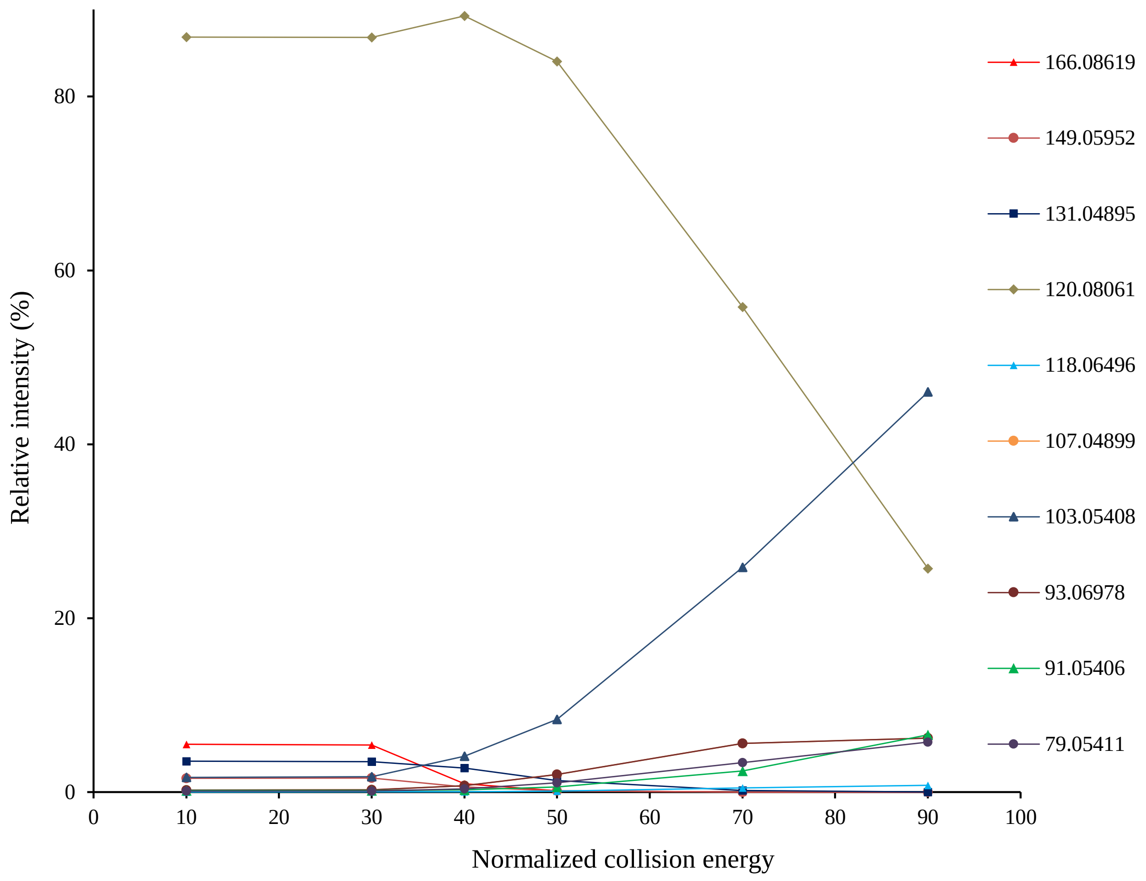

Supplementary Figure S18. Fragmentation graph of protonated Phe under different collision energies.

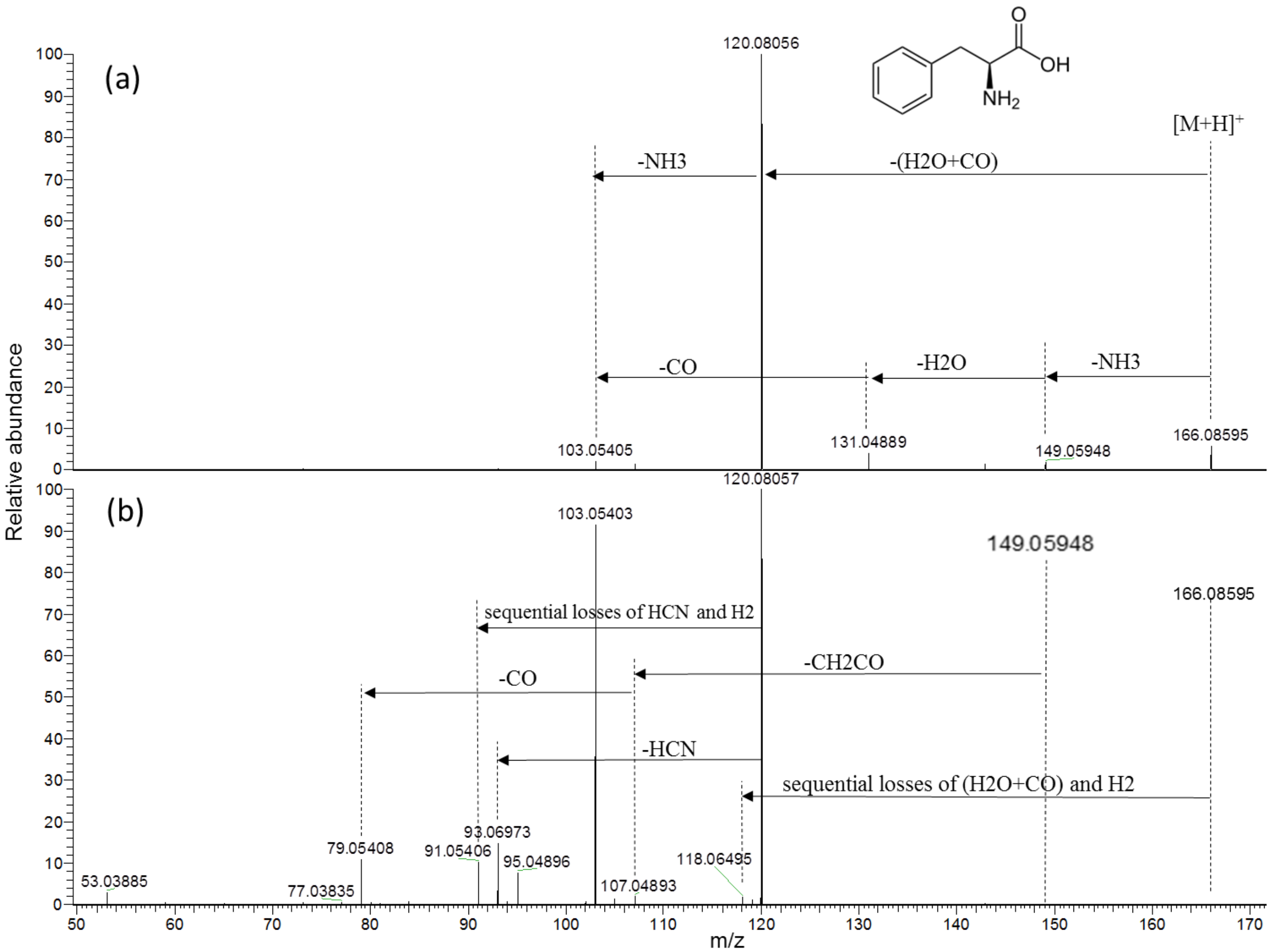

Supplementary Figure S19. Representative MS/MS spectra of protonated Phe acquired using collision energy NCE 30% (a) and 70% (b).

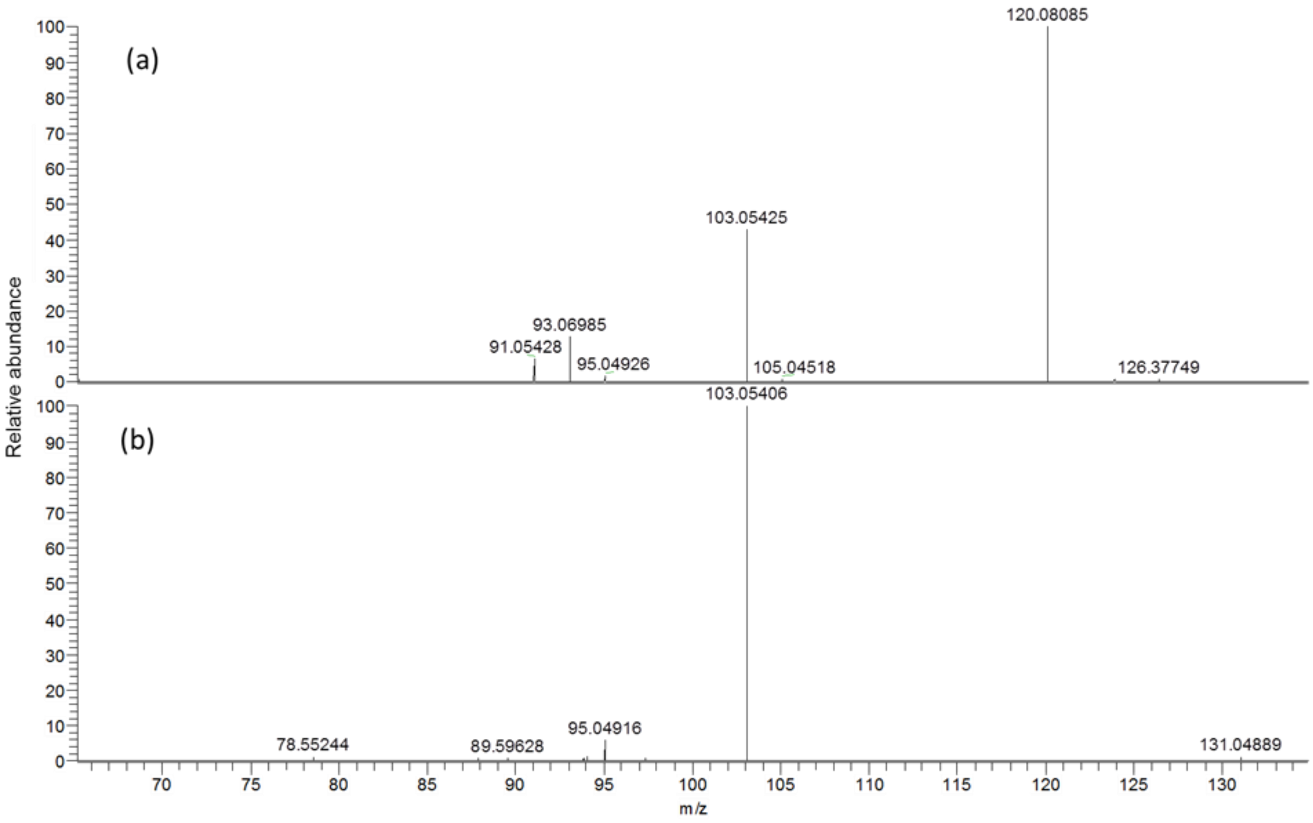

Supplementary Figure S20. Pseudo MS^3 spectra of fragment ions at *m/z* 120.08061 (a) and *m/z* 131.04895 (b) from protonated Phe. The fragment ion at *m/z* 103.05408 was observed in both spectra.

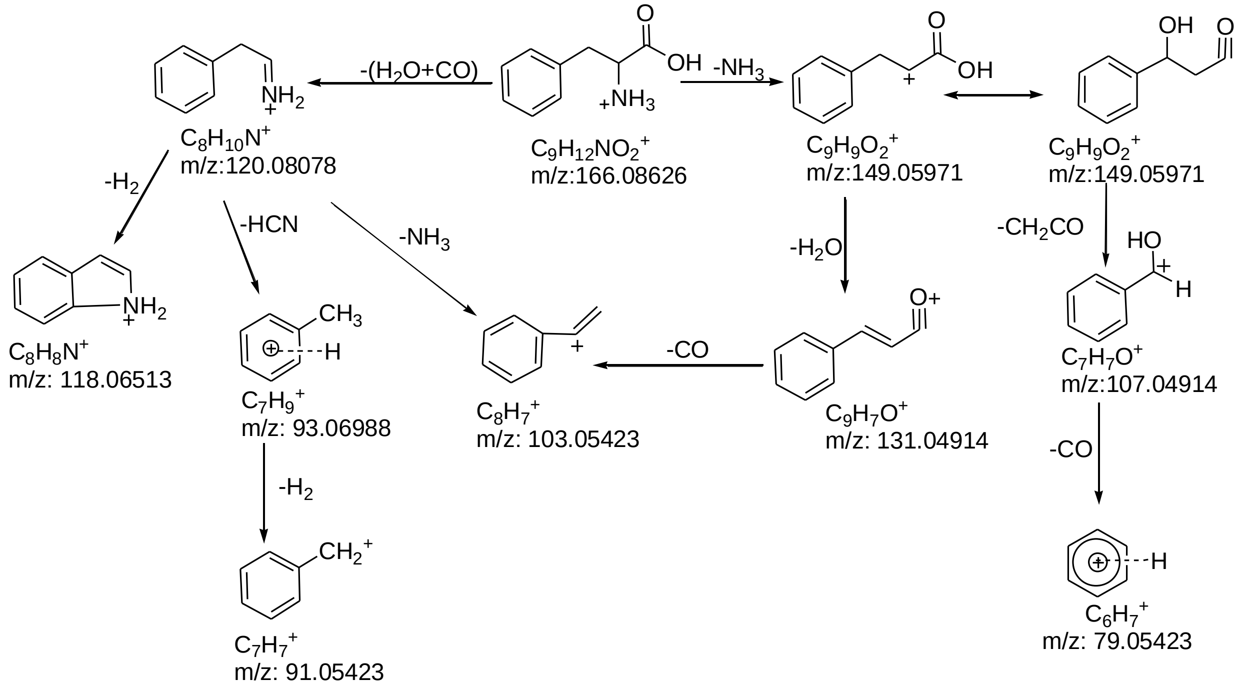

Supplementary Figure S21. Postulated fragmentation pathways of protonated Phe. The theoretical *m/z* value of each fragment ion is provided under the chemical formula.

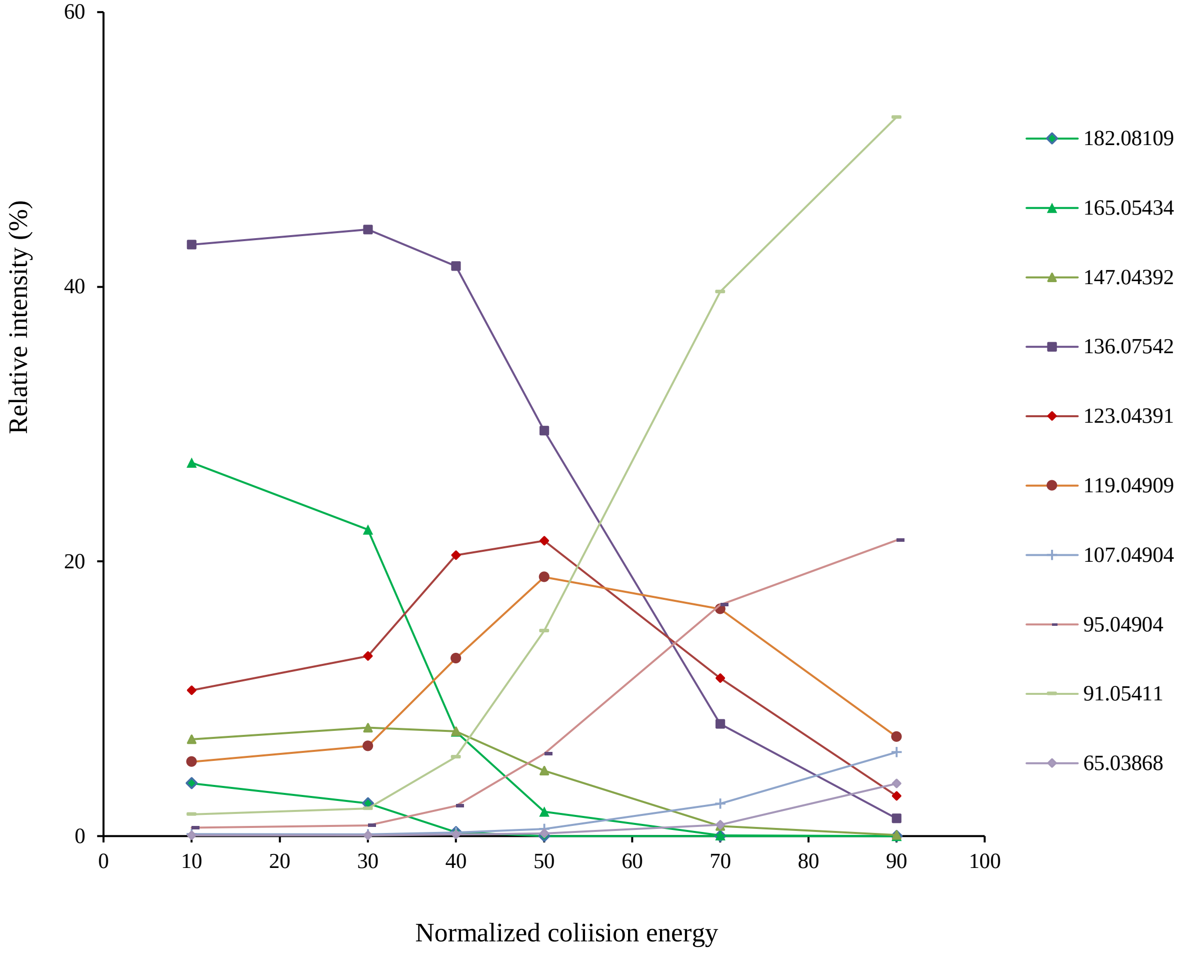

Supplementary Figure S22. Fragmentation graph of protonated Tyr under different collision energies

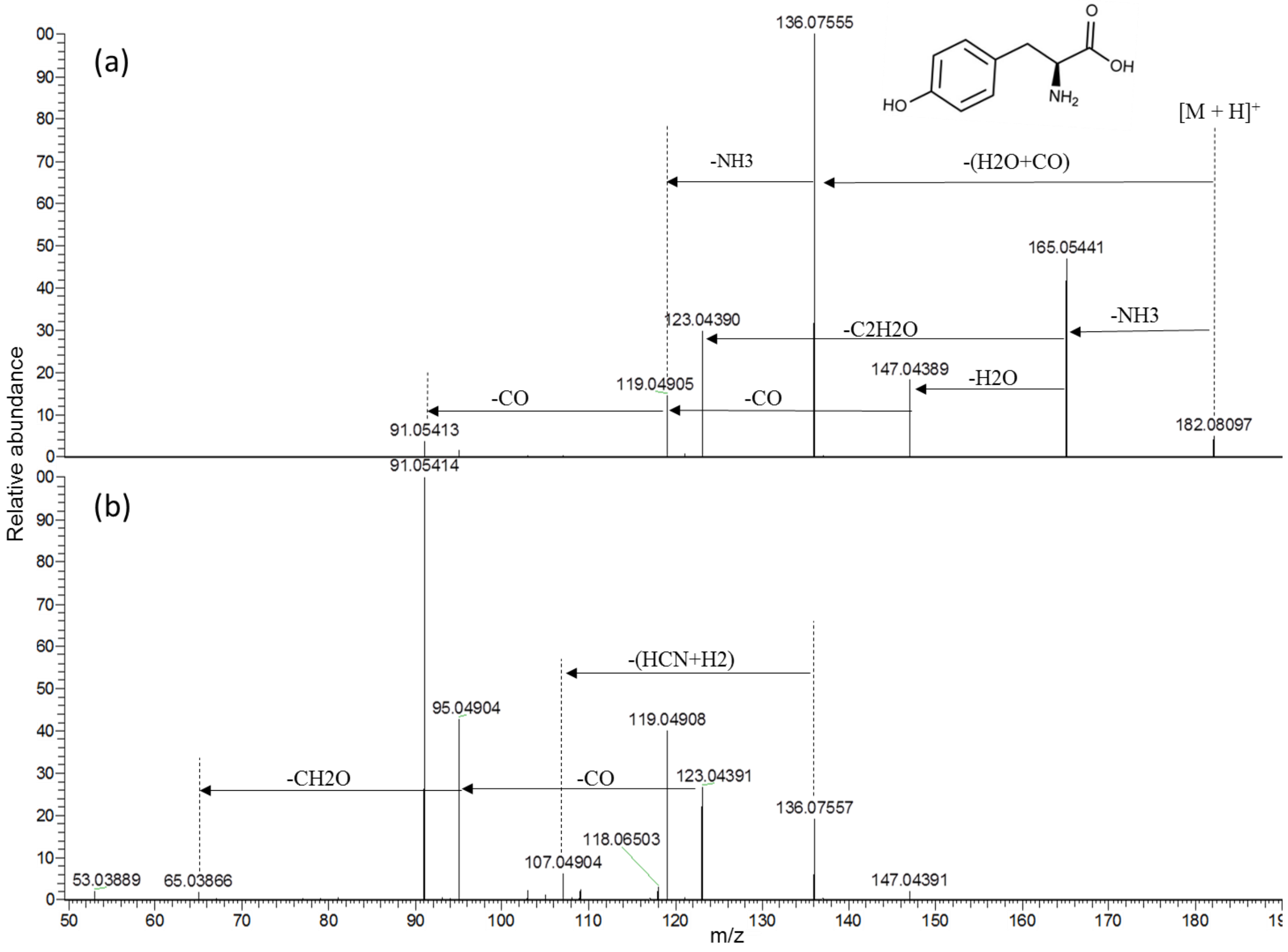

Supplementary Figure S23. Representative MS/MS spectra of protonated Tyr acquired using collision energy NCE 30% (a) and 70% (b).

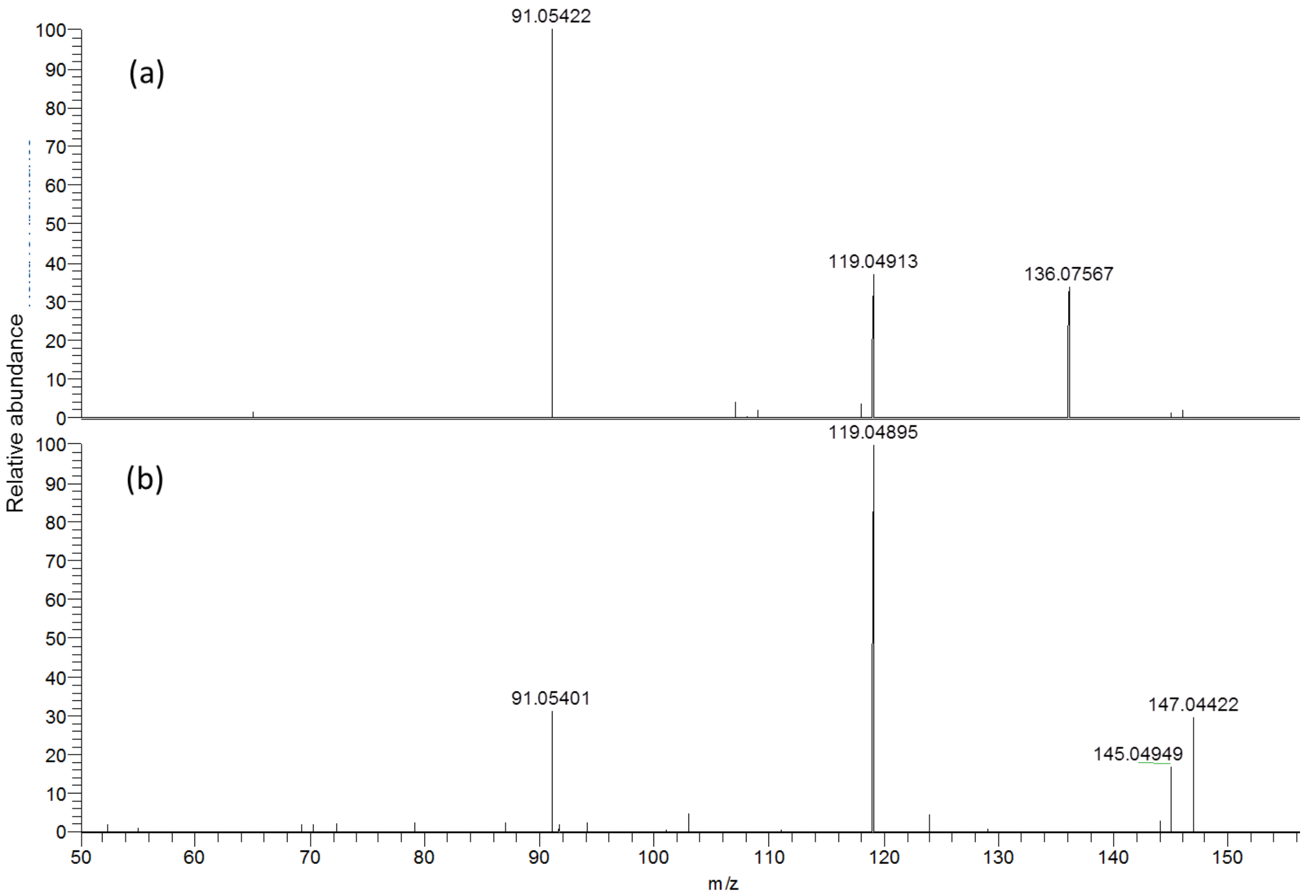

Supplementary Figure S24. Pseudo MS^3 spectrum of fragment ions at *m/z* 136.07542 (a) and *m/z* 147.04392 (b) from protonated Tyr observed the same fragment at *m/z* 119.04909.

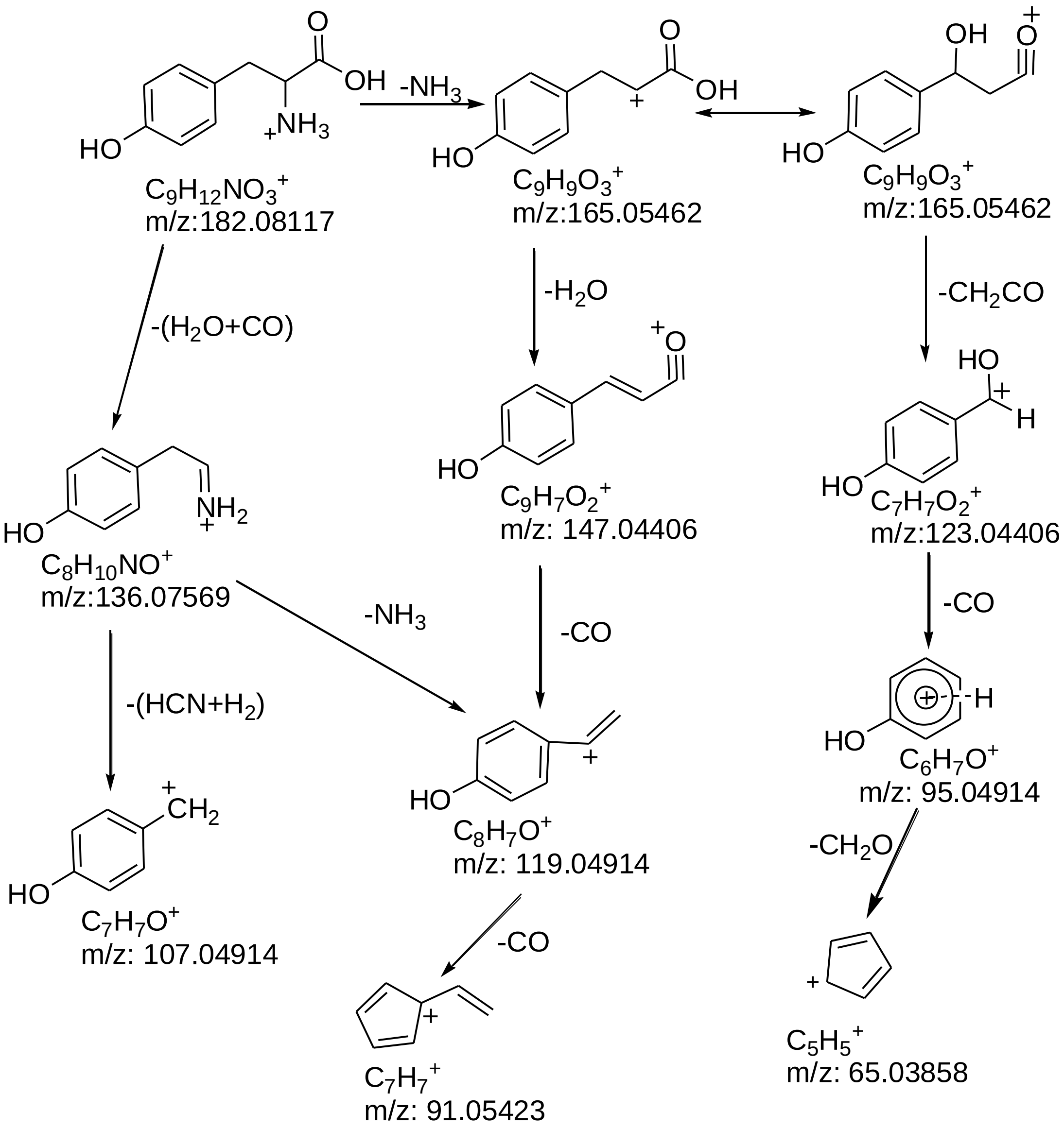

Supplementary Figure S25. Postulated fragmentation pathways of protonated Tyr. The theoretical *m/z* value of each fragment ion is provided under the chemical formula.

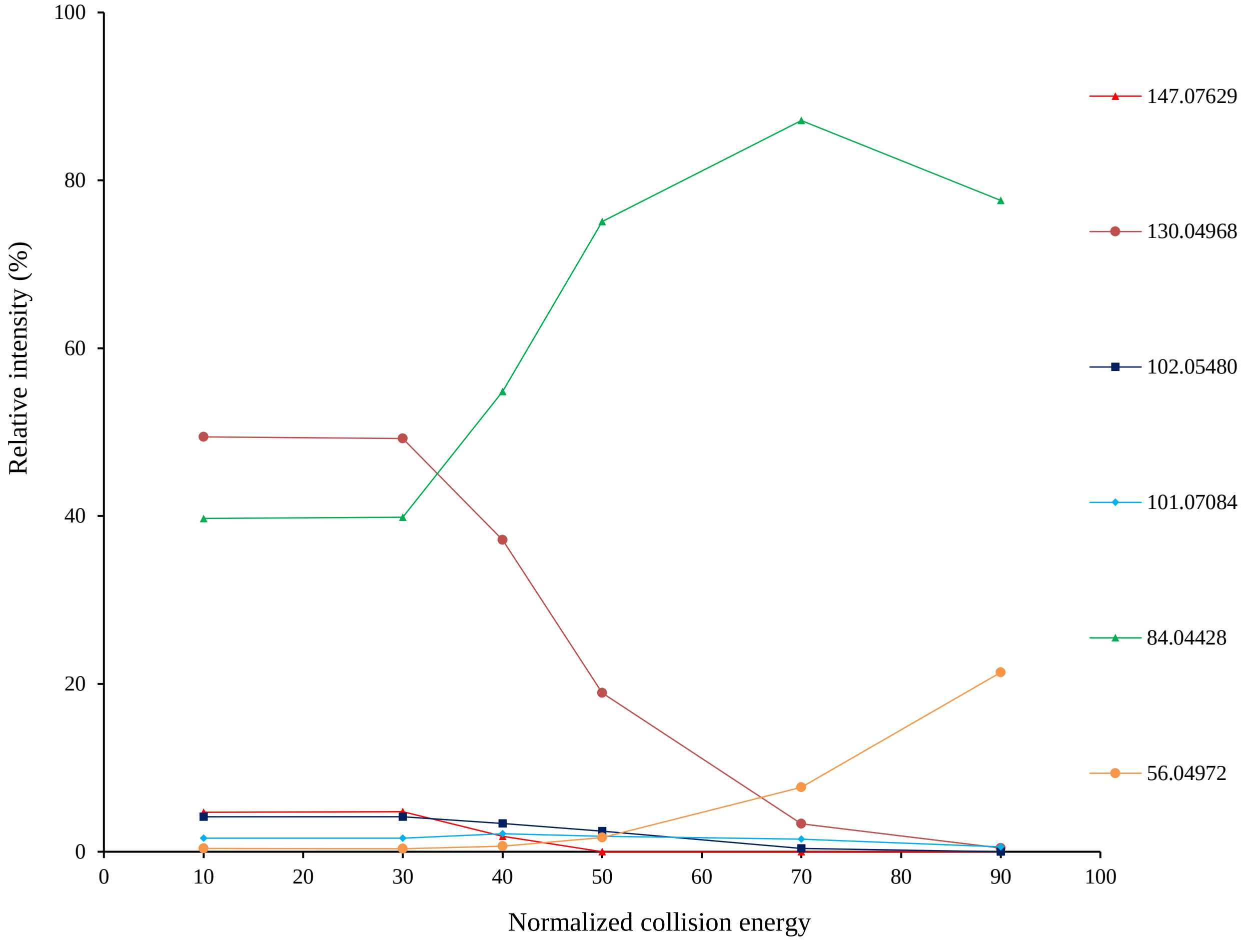

Supplementary Figure S26. Fragmentation graph of protonated Gln under different collision energies.

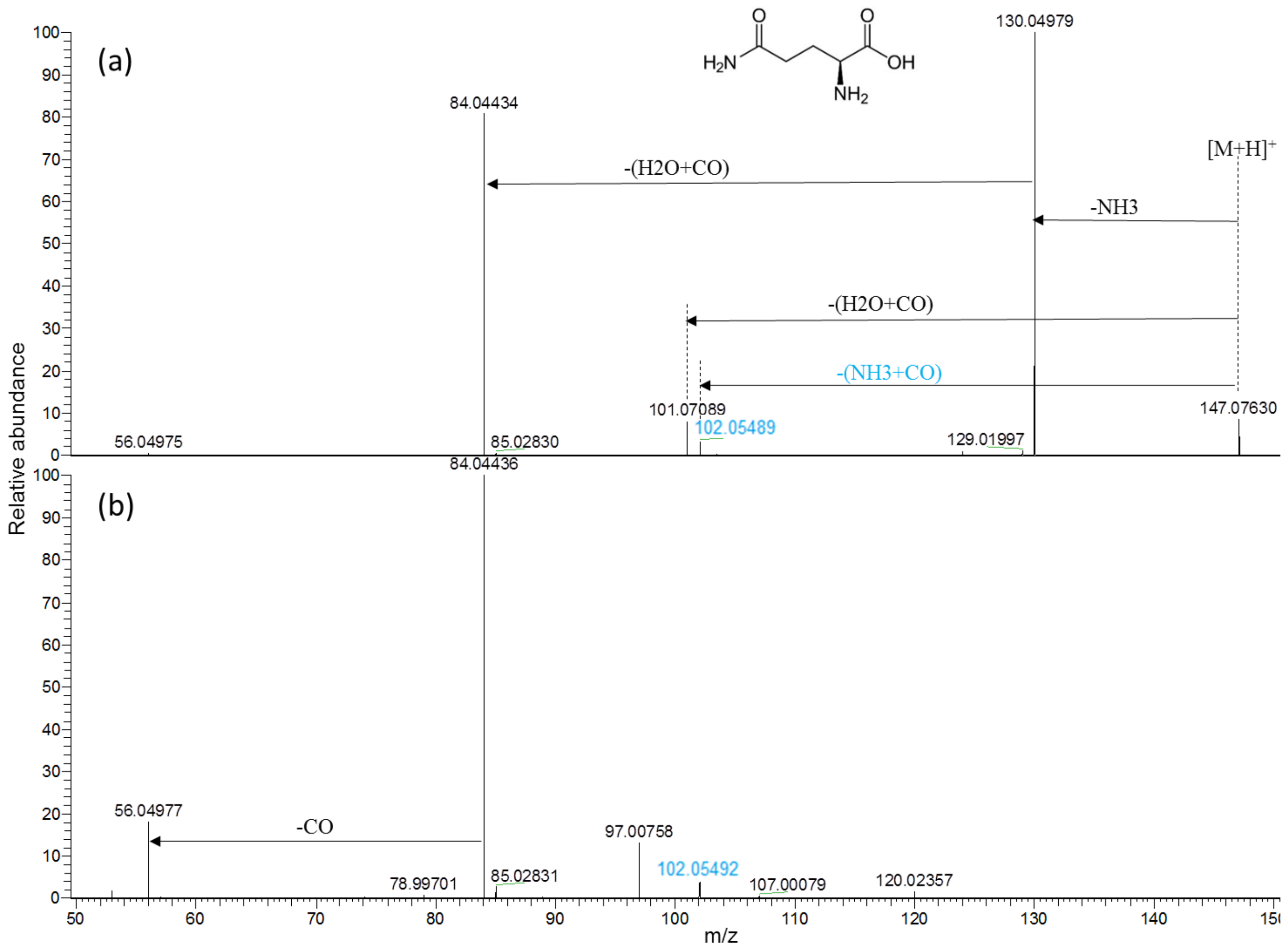

Supplementary Figure S27. Representative MS/MS spectra of protonated Gln acquired using collision energy NCE 30% (a) and 70% (b). The previously unreported fragment ion is shown in blue.

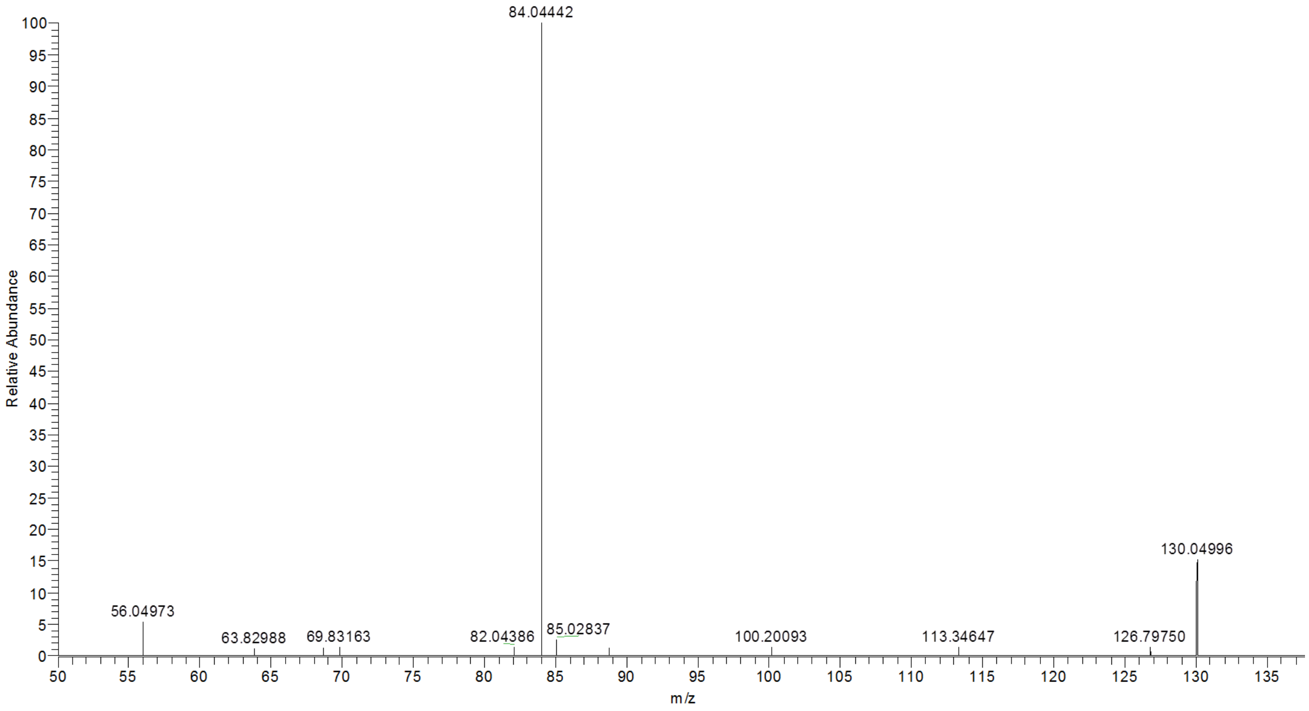

Supplementary Figure S28. Pseudo MS^3 spectrum of fragment ions at *m/z* 130.04968 from protonated Gln. There was no sign of elimination of CO from *m/z* 130.04968.

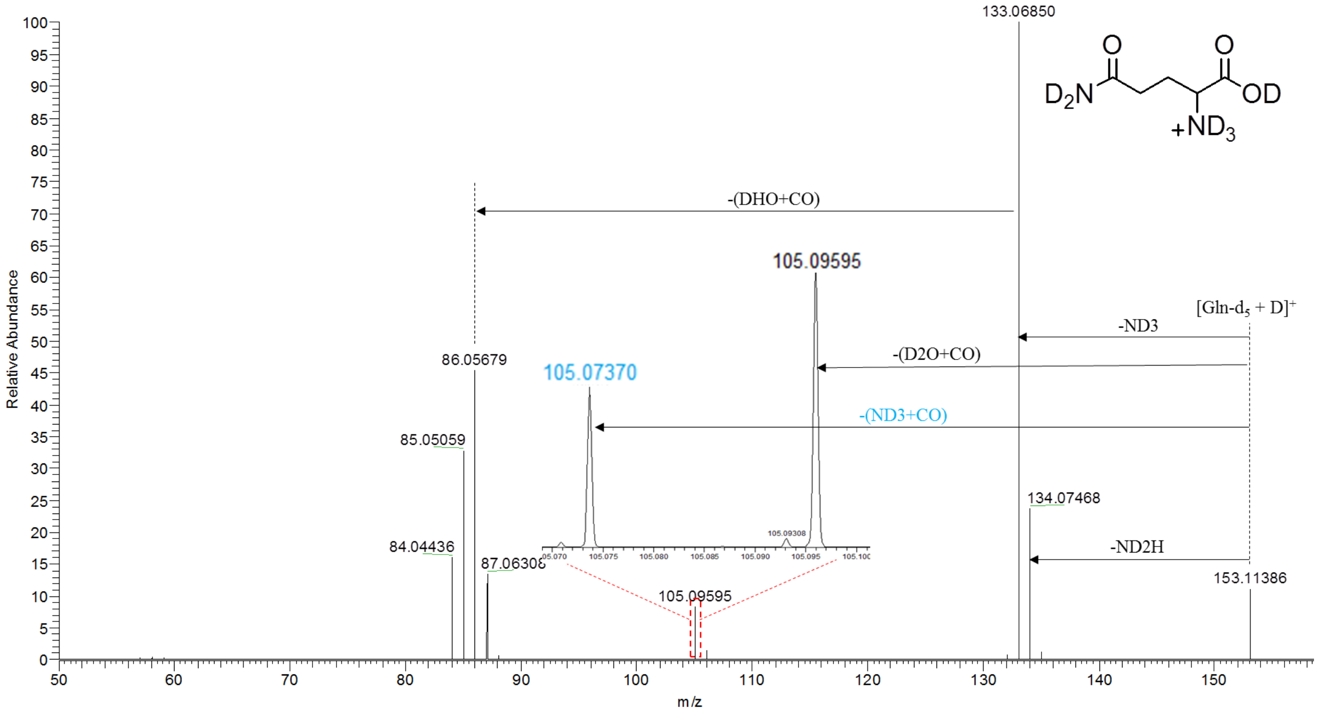

Supplementary Figure S29. Representative MS/MS spectra of $[Gln\text{-}d_5+D]^+$ under collision energy NCE 30%. The previously unreported fragment ion is shown in blue.

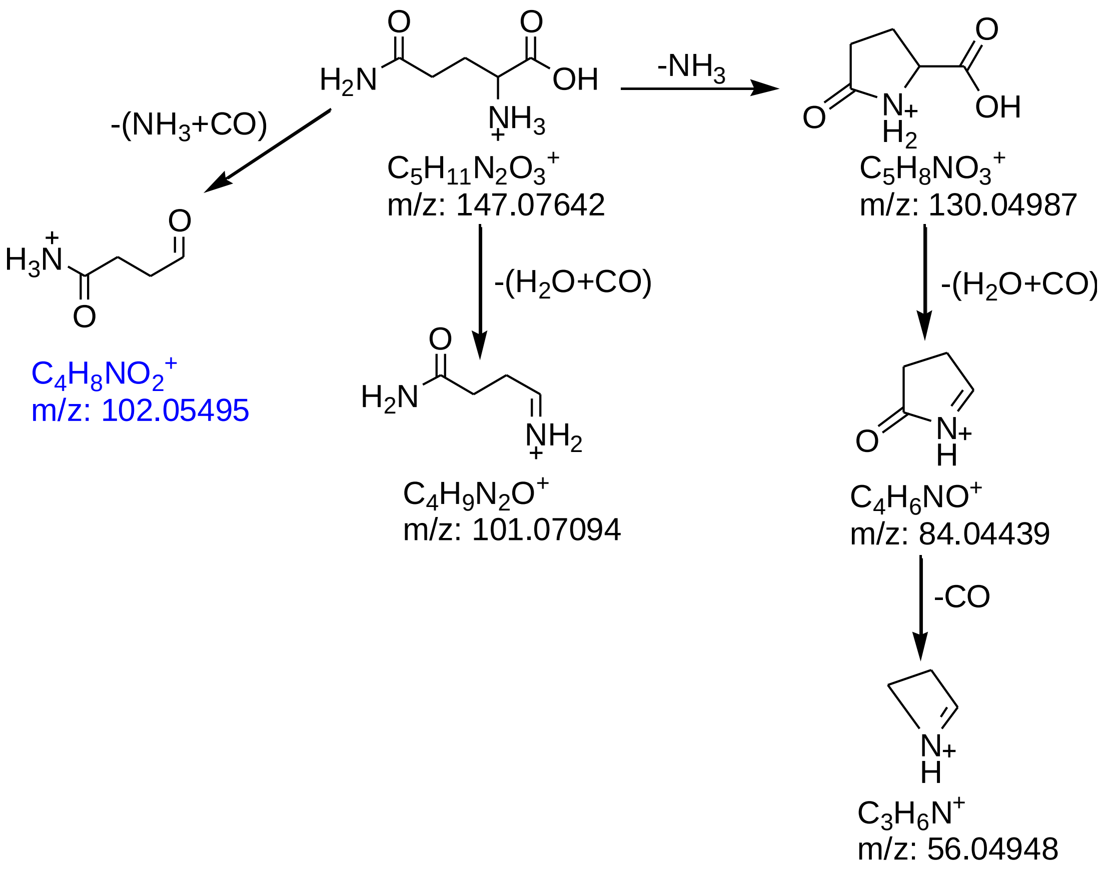

Supplementary Figure S30. Postulated fragmentation pathways of protonated Gln. The previously unreported fragment ion is shown in blue. The theoretical *m/z* value of each fragment ion is provided under the chemical formula.

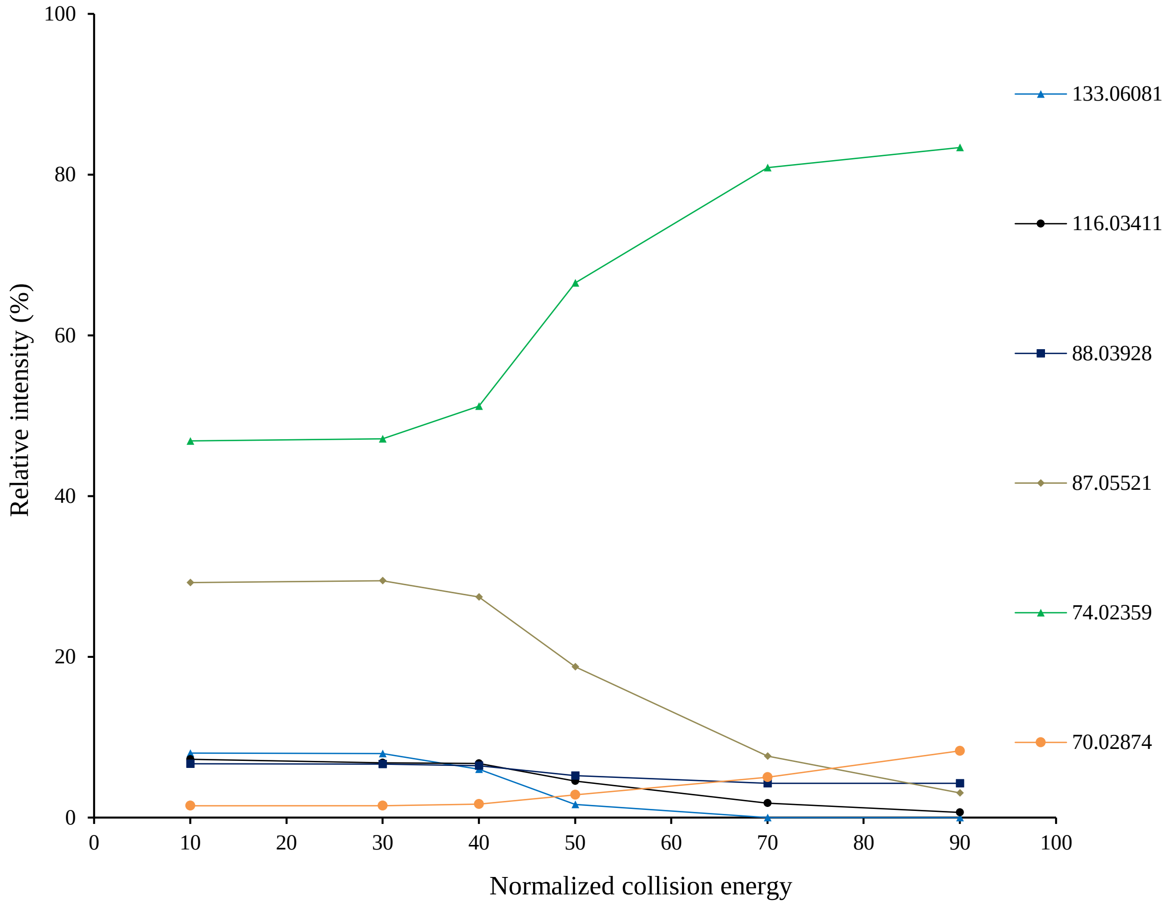

Supplementary Figure S31. Fragmentation graph of protonated Asn under different collision energies.

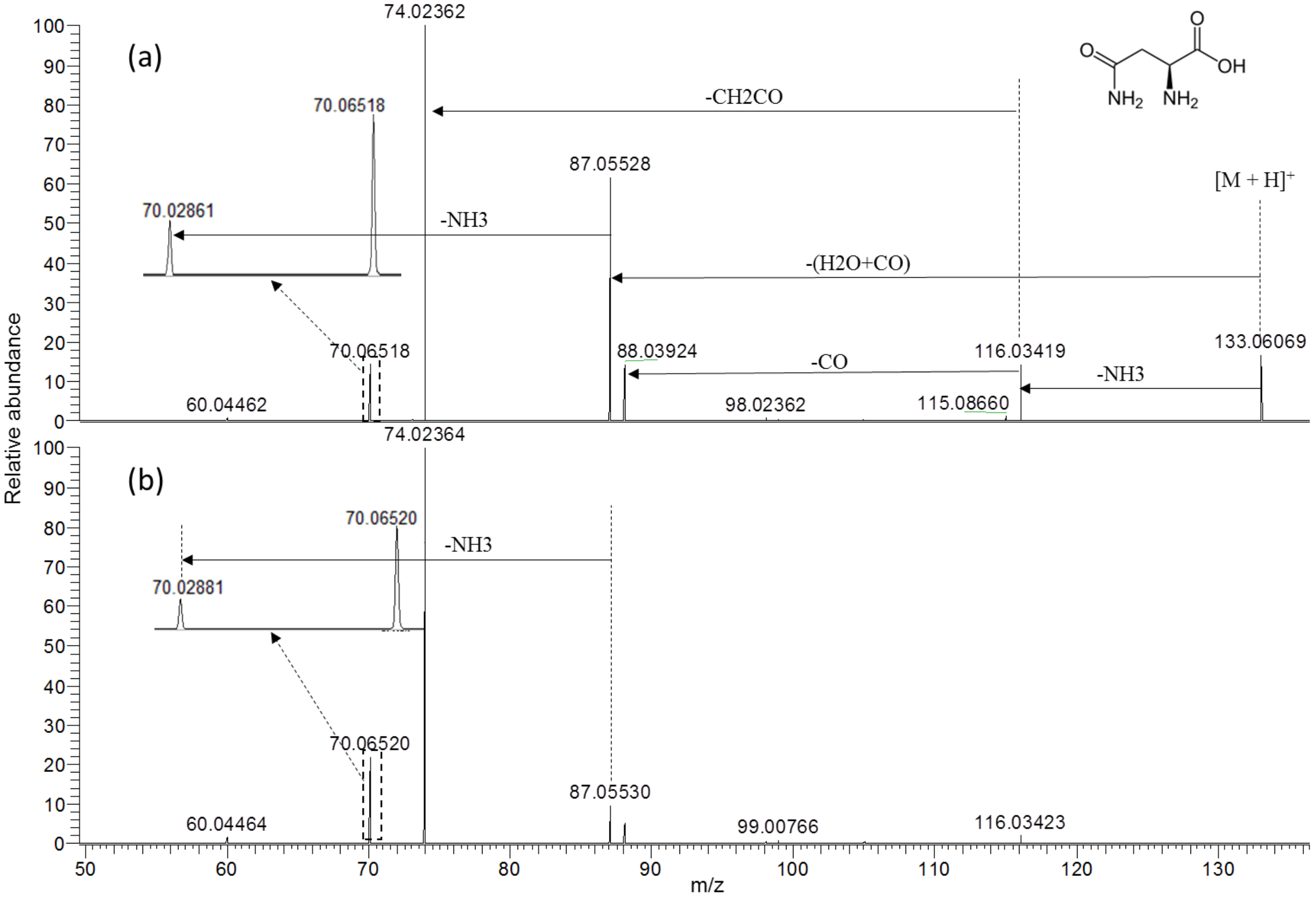

Supplementary Figure S32 Representative MS/MS spectra of protonated Asn acquired using collision energy NCE 30% (a) and 70% (b).

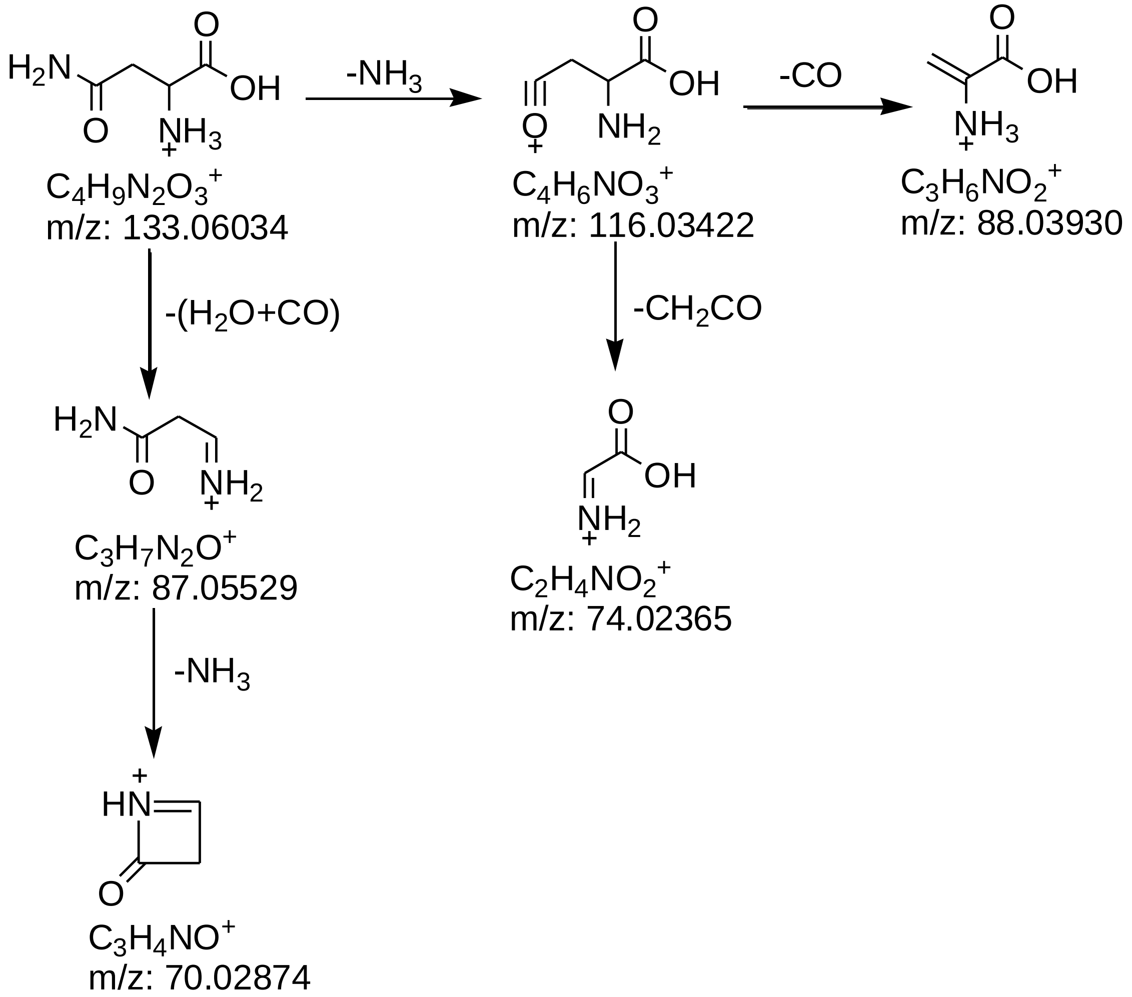

Supplementary Figure S33. Postulated fragmentation pathways of protonated Asn. The theoretical *m/z* value of each fragment ion is provided under the chemical formula. The theoretical *m/z* value of each fragment ion is provided under the chemical formula.

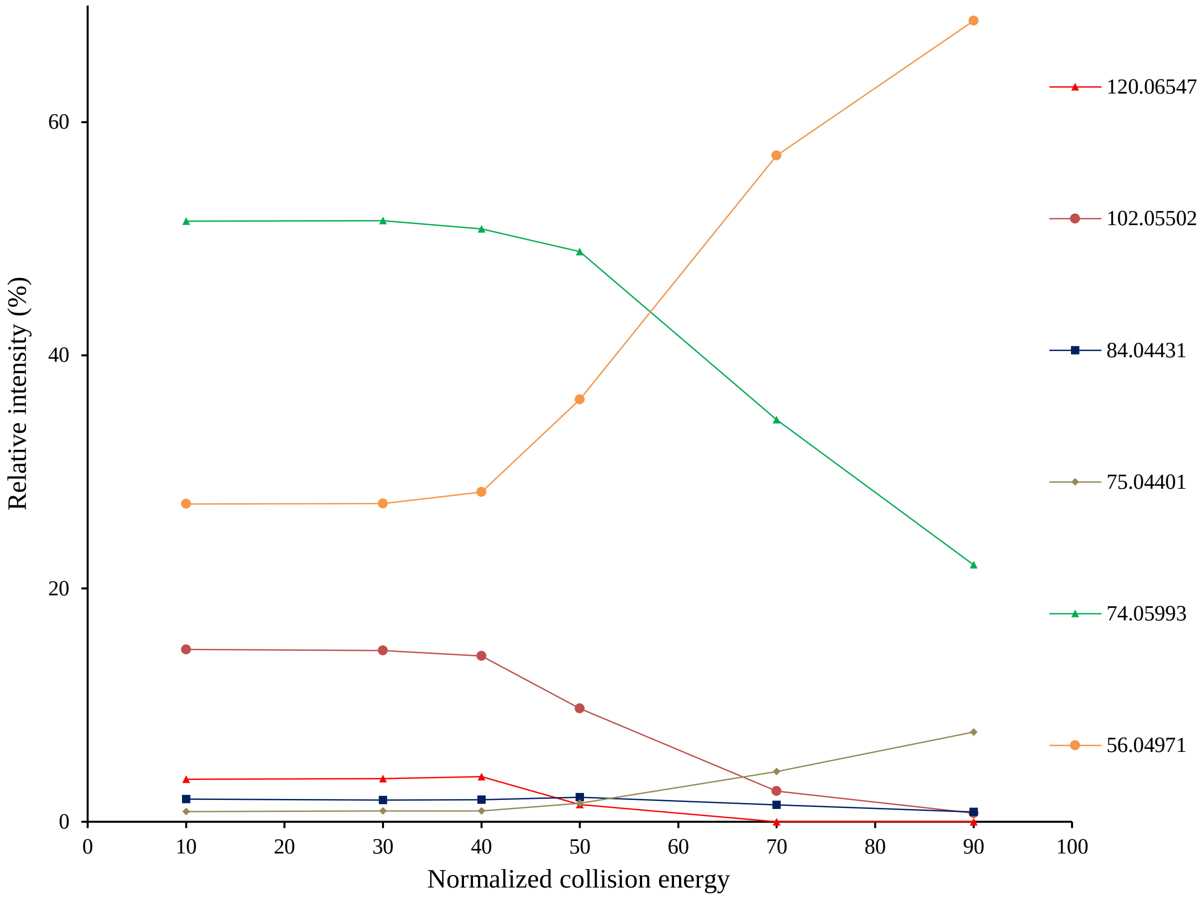

Supplementary Figure S34. Fragmentation graph of protonated Thr under different collision energies.

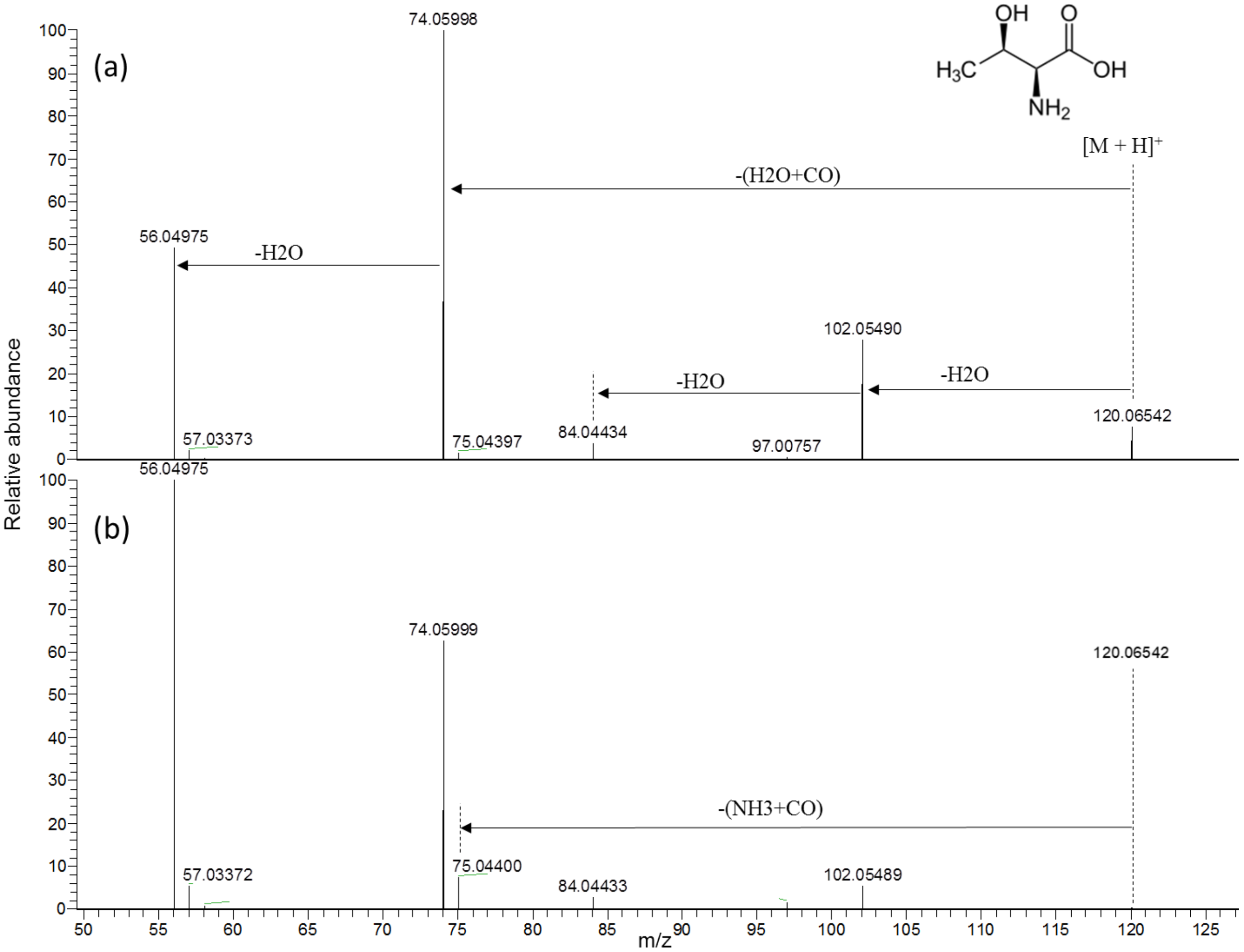

Supplementary Figure S35. Representative MS/MS spectra of protonated Thr acquired using collision energy NCE 30% (a) and 70% (b).

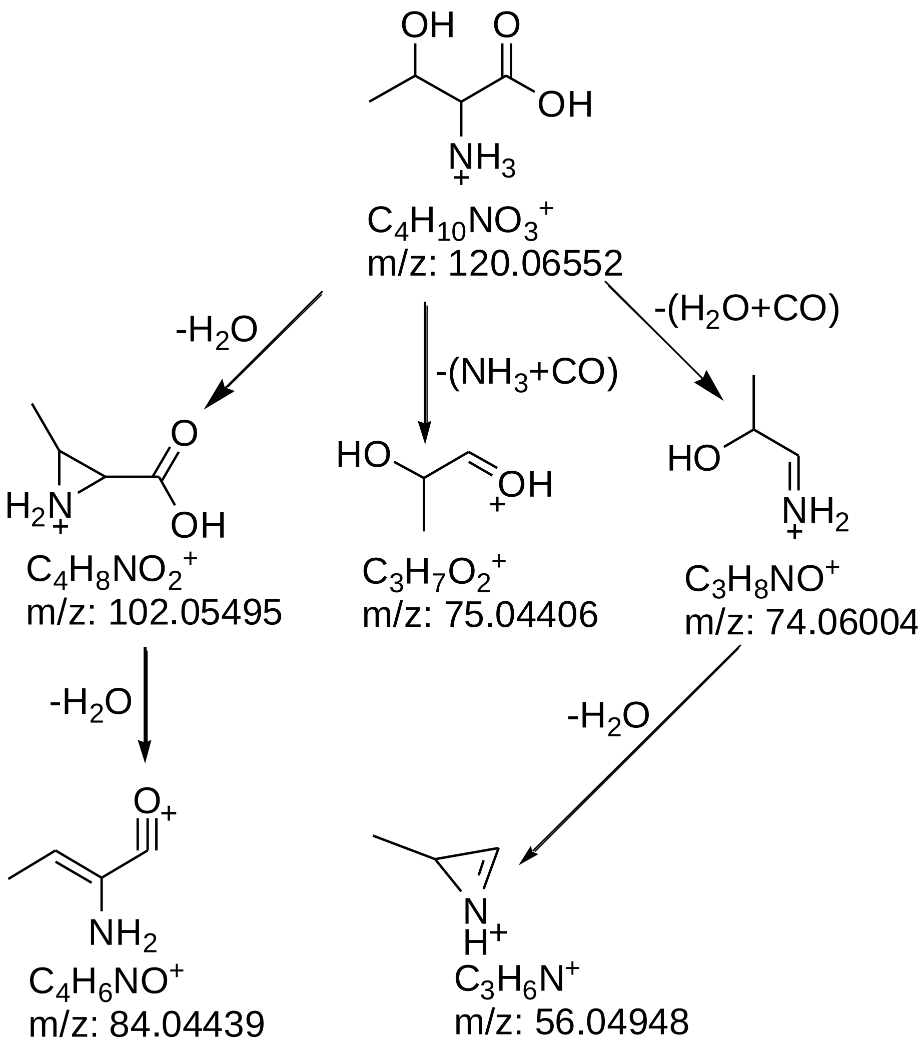

Supplementary Figure S36. Postulated fragmentation pathways of protonated Thr. The theoretical *m/z* value of each fragment ion is provided under the chemical formula.

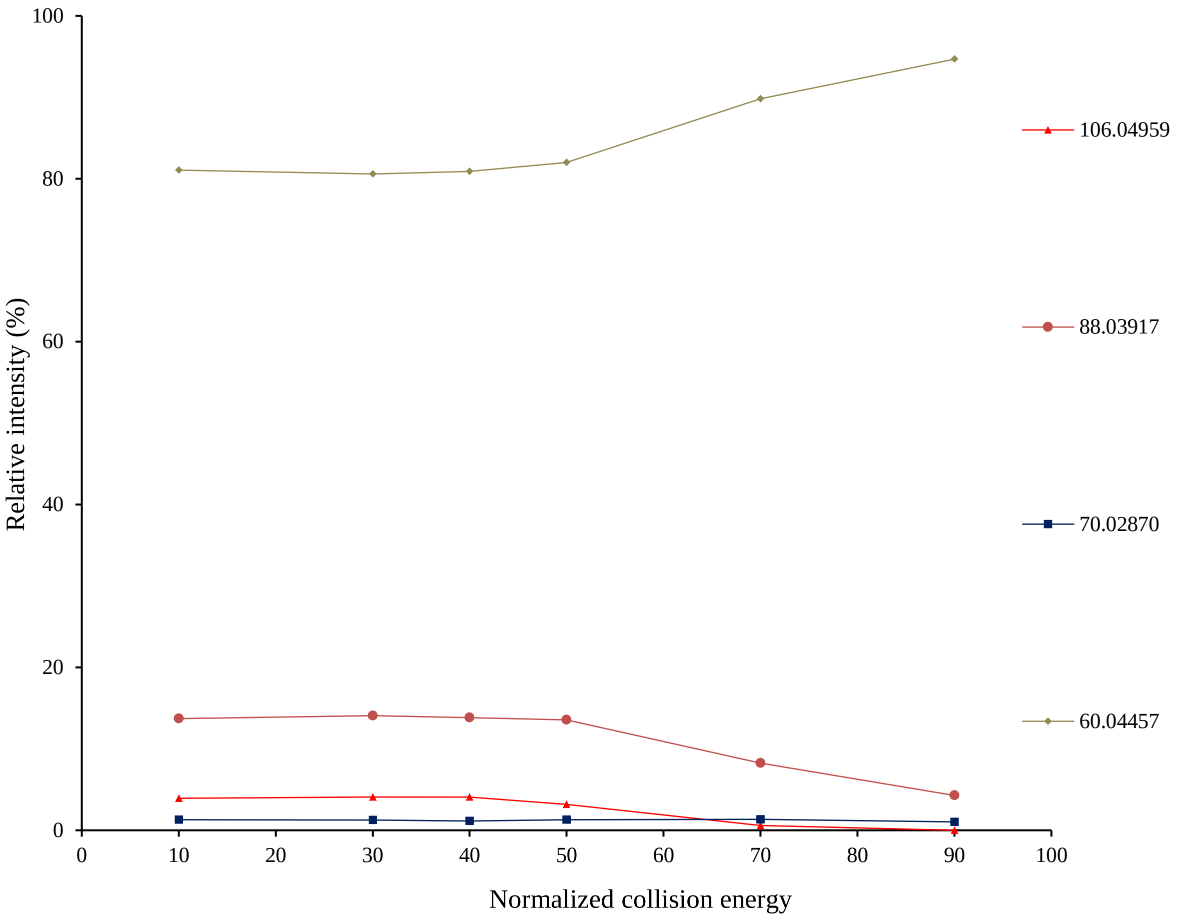

Supplementary Figure S37. Fragmentation graph of protonated Ser under different collision energies.

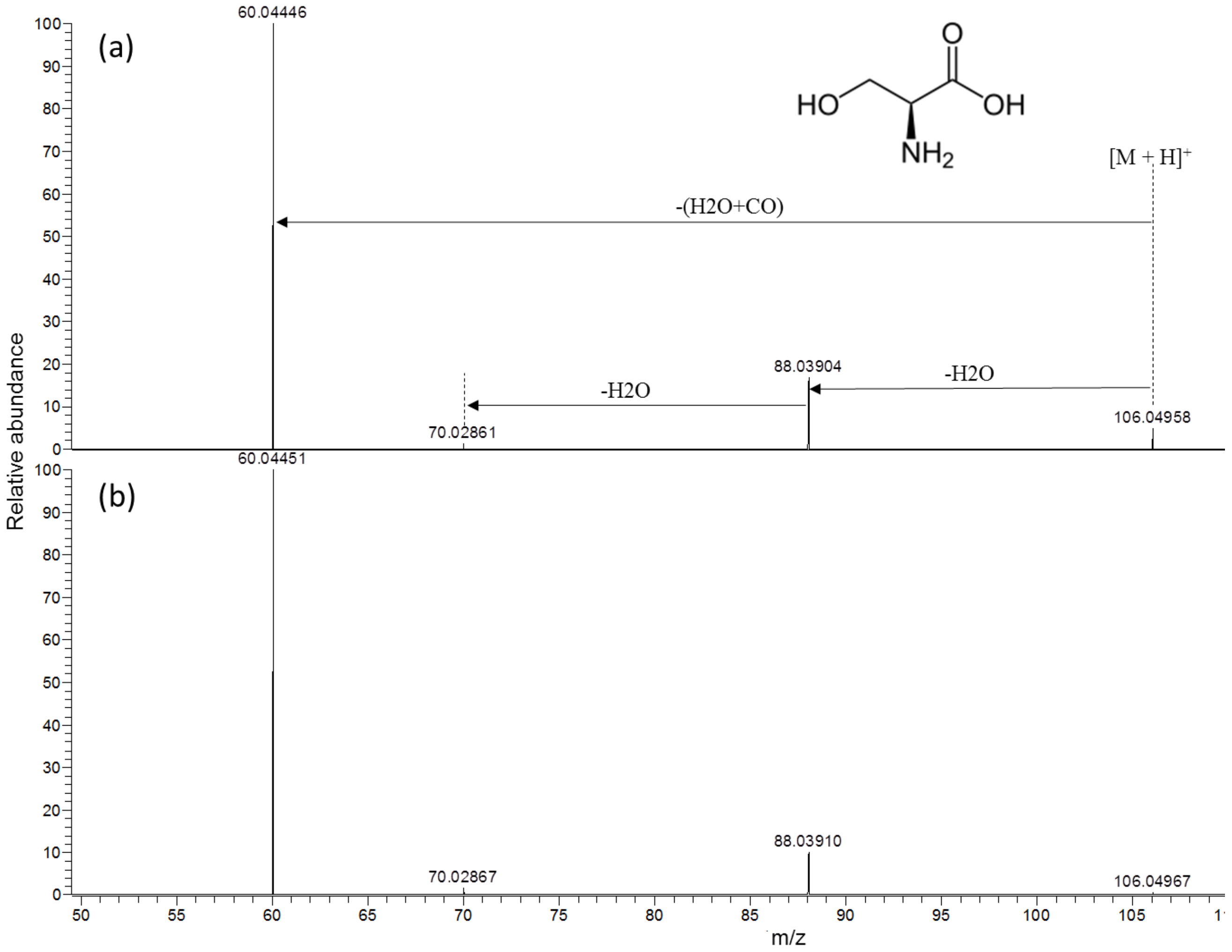

Supplementary Figure S38. Representative MS/MS spectra of protonated Ser acquired using collision energy NCE 30% (a) and 70% (b).

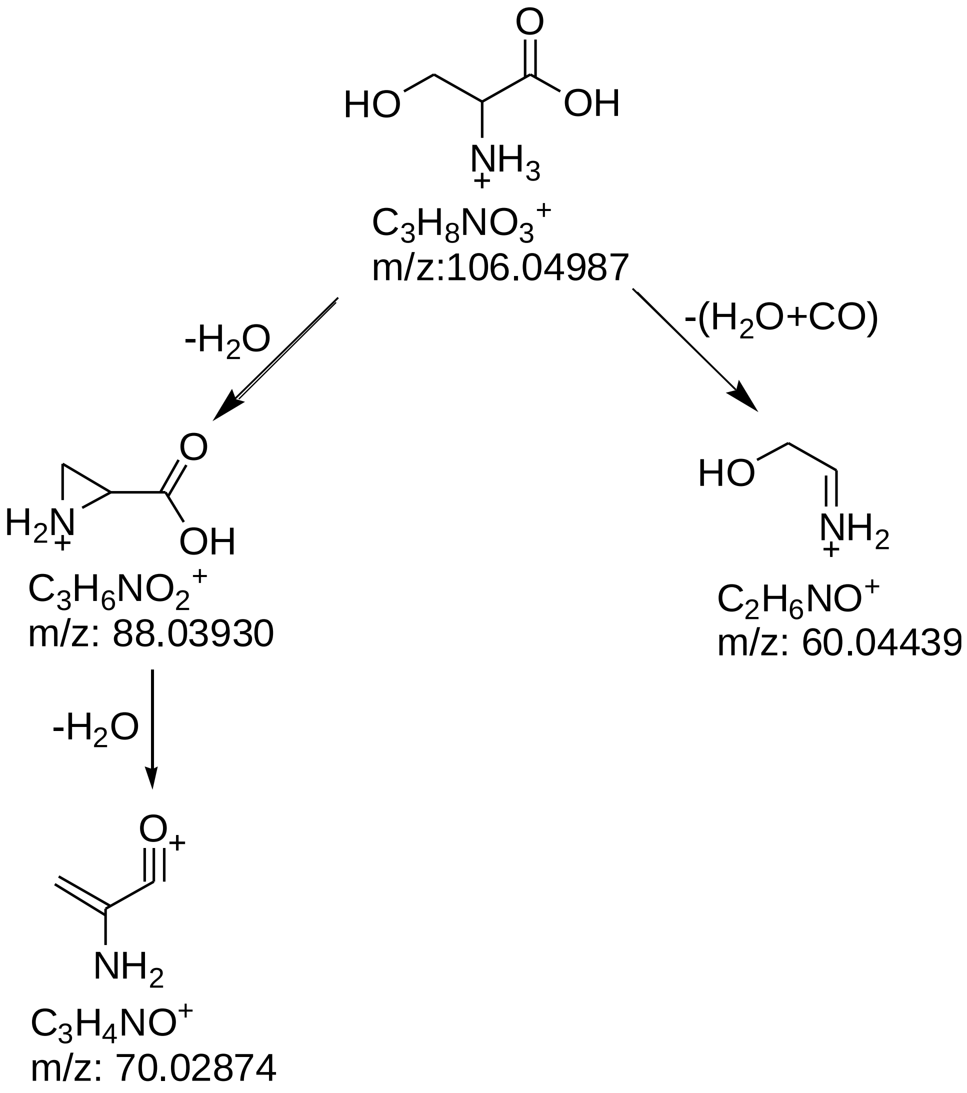

Supplementary Figure S39. Postulated fragmentation pathways of protonated Ser. The theoretical *m/z* value of each fragment ion is provided under the chemical formula.

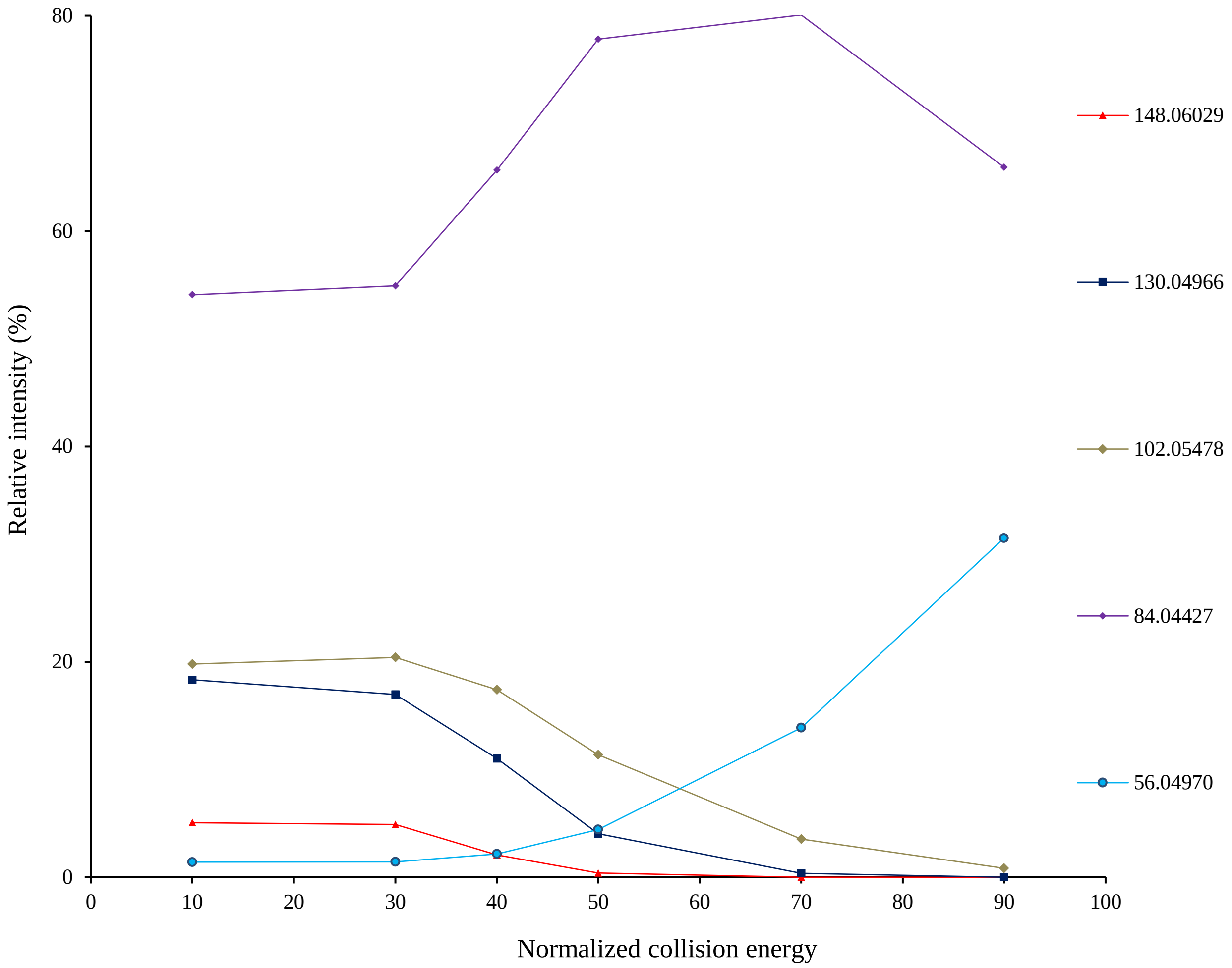

Supplementary Figure S40. Fragmentation graph of protonated Glu under different collision energies.

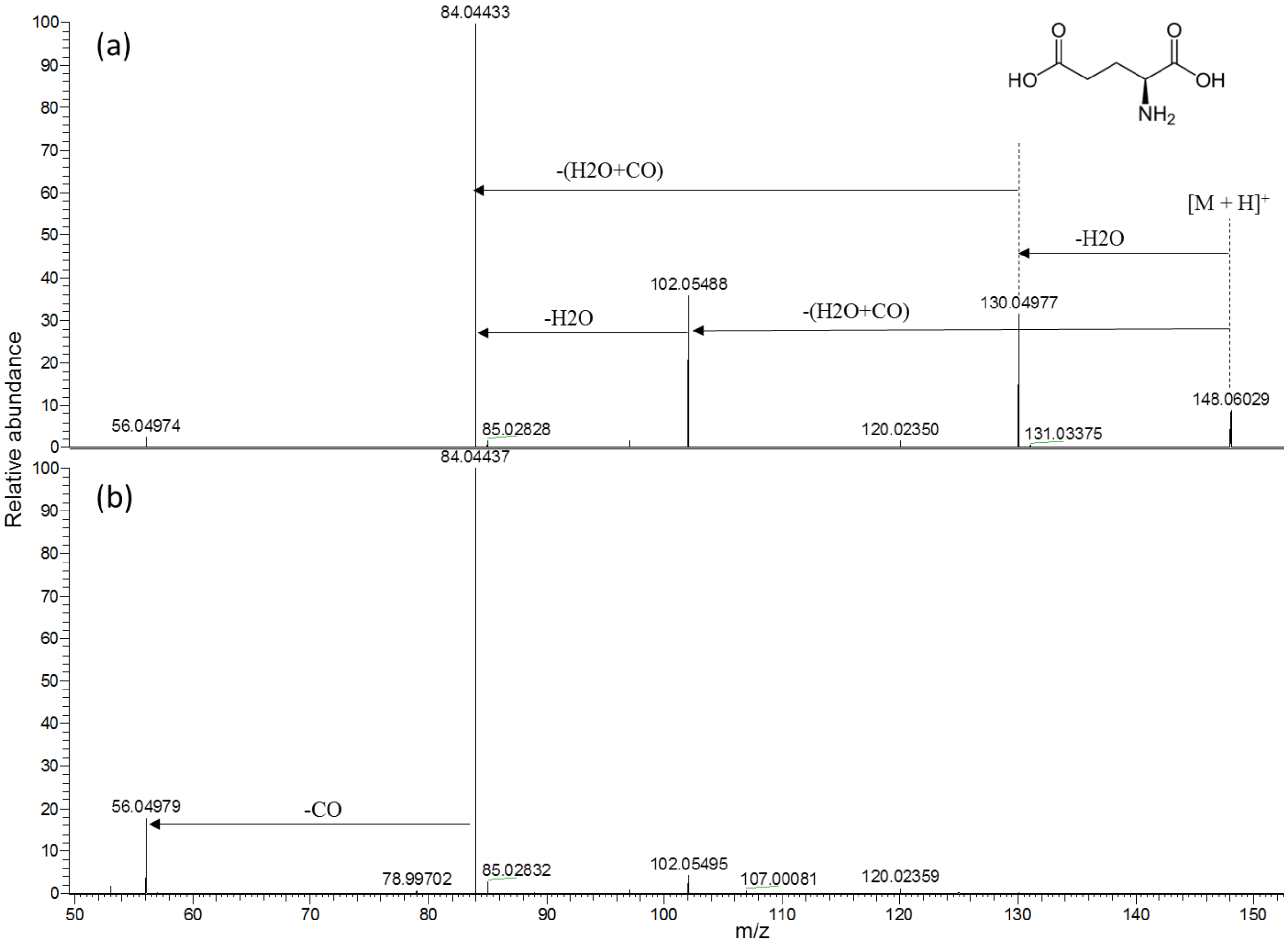

Supplementary Figure S41 Representative MS/MS spectra of protonated Glu acquired using collision energy NCE 30% (a) and 70% (b).

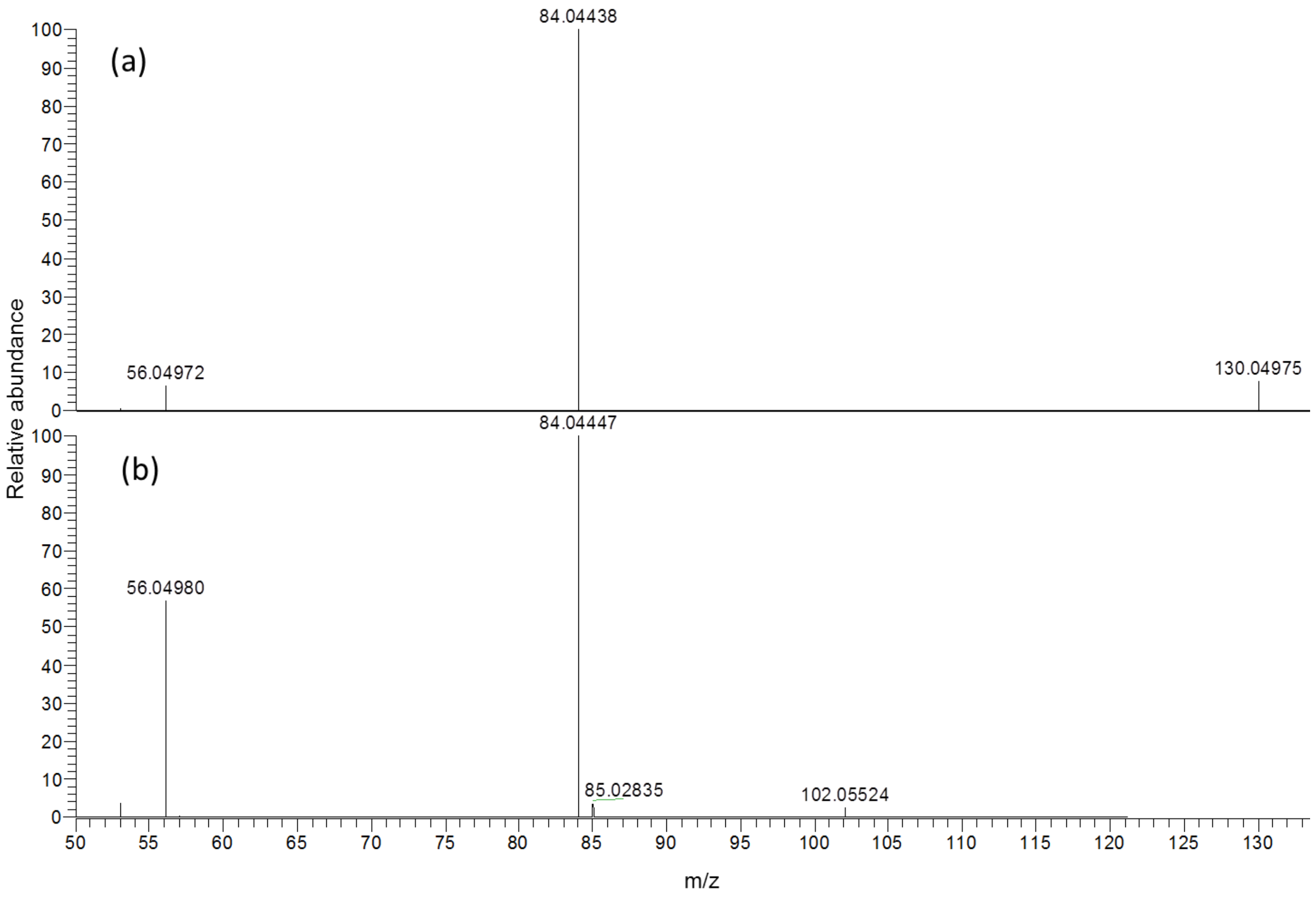

Supplementary Figure S42. Pseudo MS^3 spectra of fragment ions at *m/z* 130.04966 (a) and *m/z* 102.05478 (b) from protonated Glu.

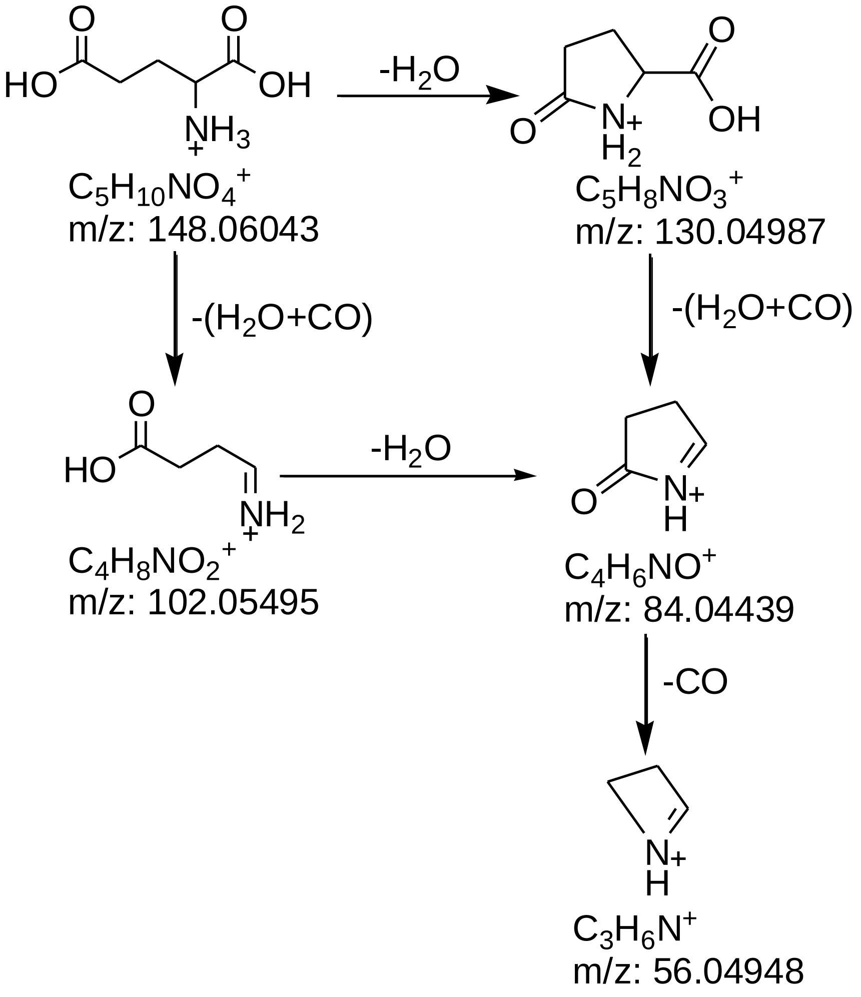

Supplementary Figure S43. Postulated fragmentation pathways of protonated Glu. The theoretical *m/z* value of each fragment ion is provided under the chemical formula.

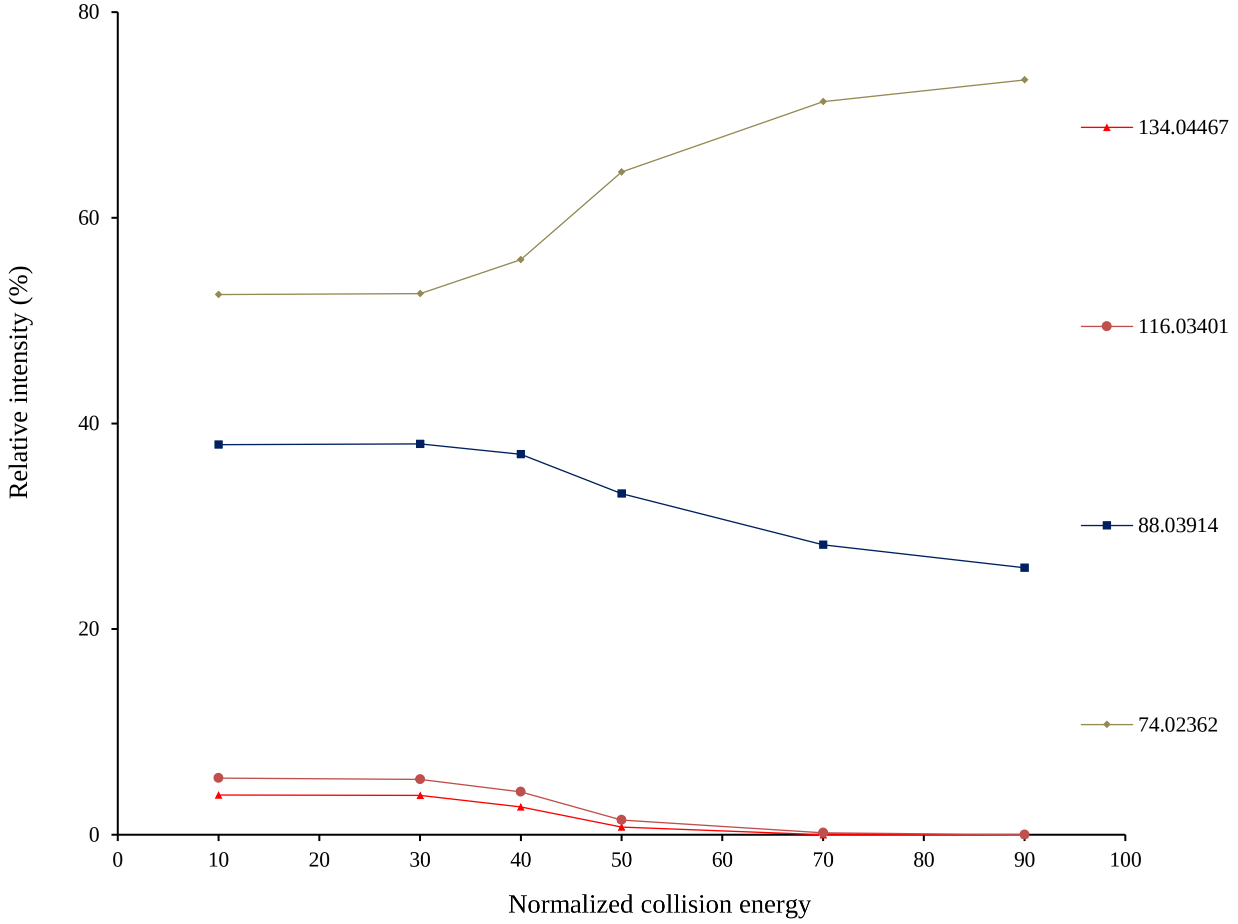

Supplementary Figure S44. Fragmentation graph of protonated Asp under different collision energies.

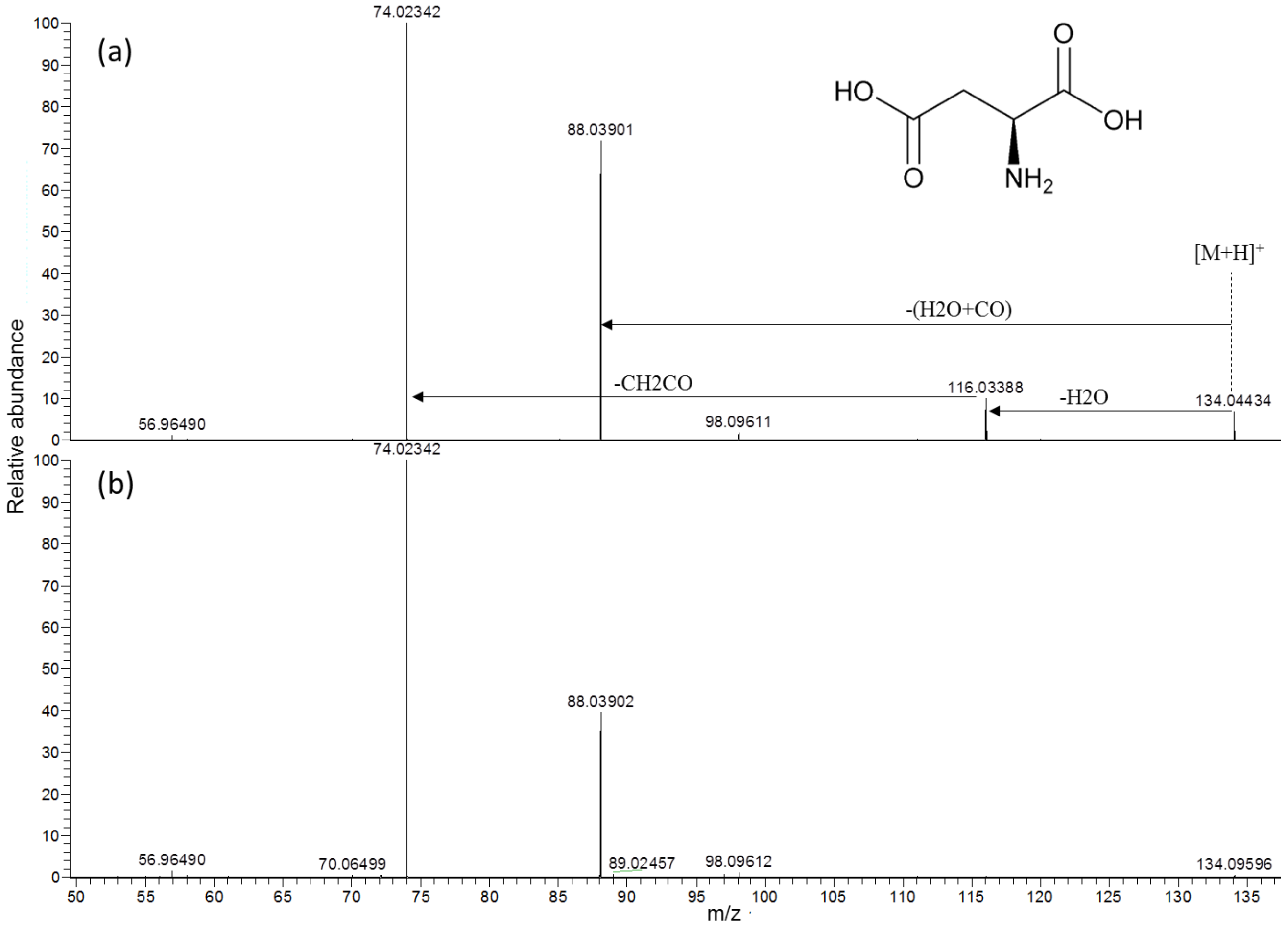

Supplementary Figure S45 Representative MS/MS spectra of protonated Asp acquired using collision energy NCE 30% (a) and 70% (b).

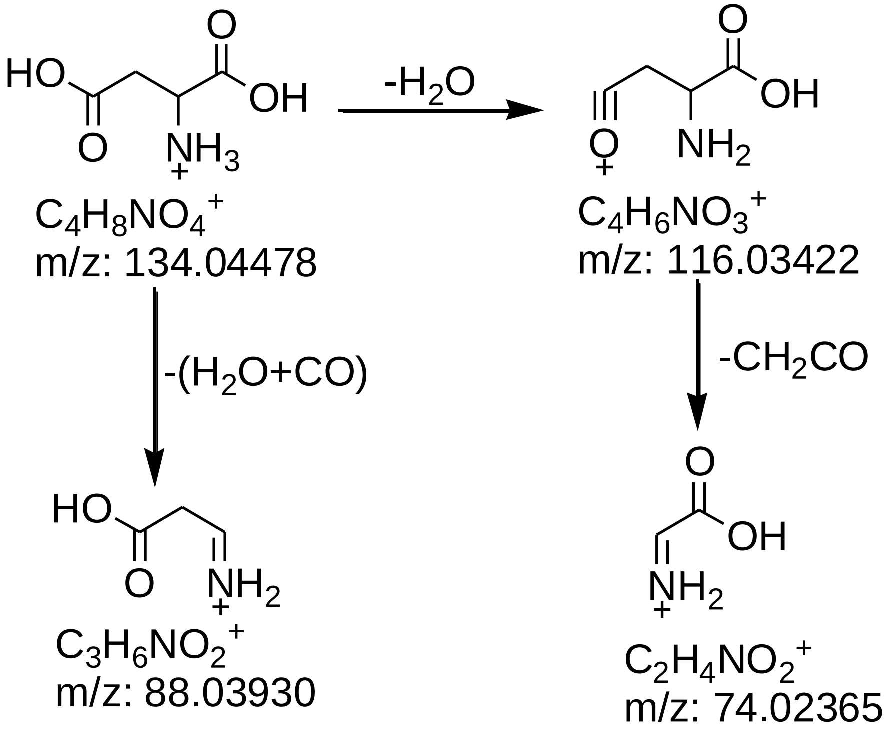

Supplementary Figure S46. Postulated fragmentation pathways of protonated Asp. The theoretical *m/z* value of each fragment ion is provided under the chemical formula.

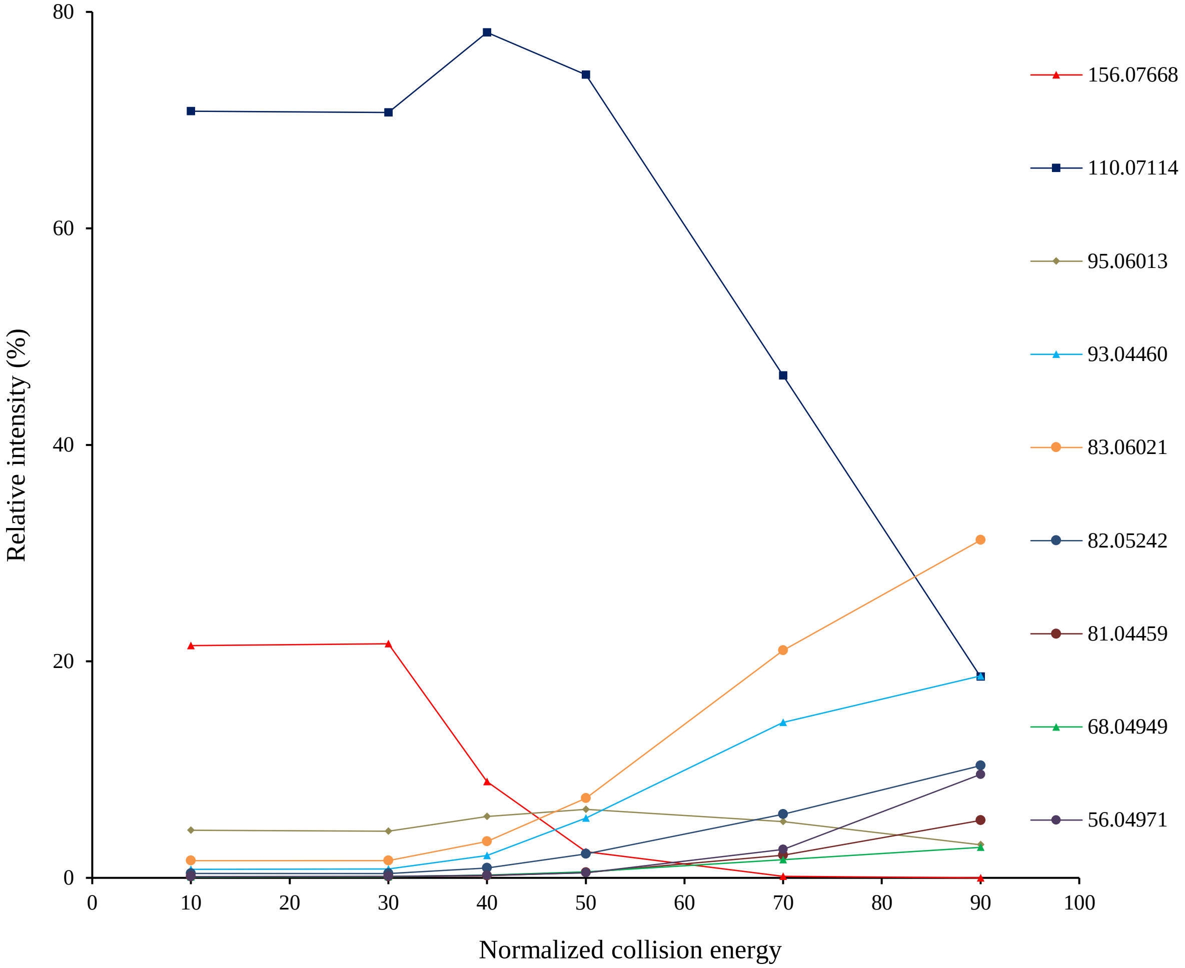

Supplementary Figure S47. Fragmentation graph of protonated His under different collision energies.

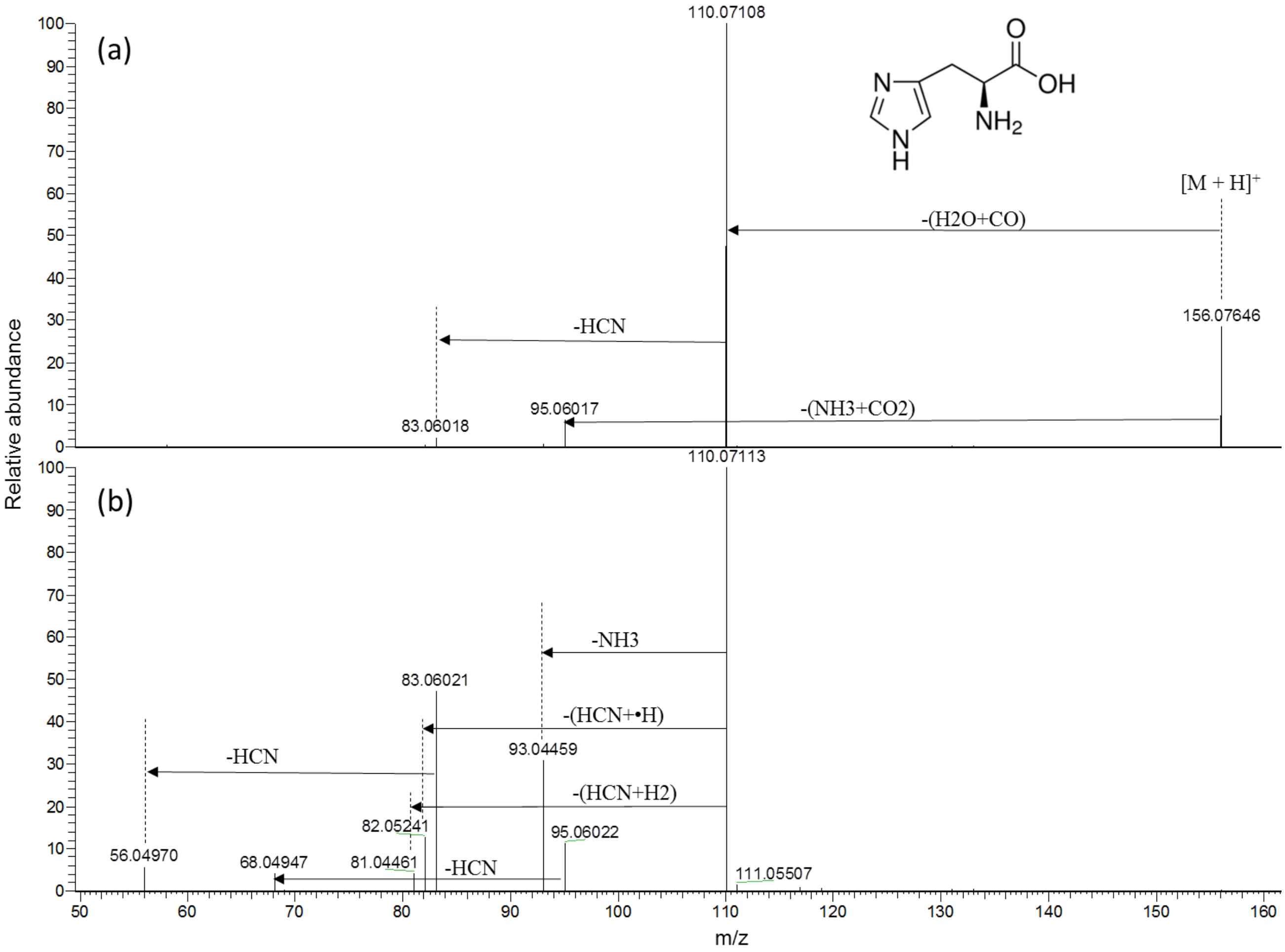

Supplementary Figure S48. Representative MS/MS spectra of protonated His acquired using collision energy NCE 30% (a) and 70% (b).

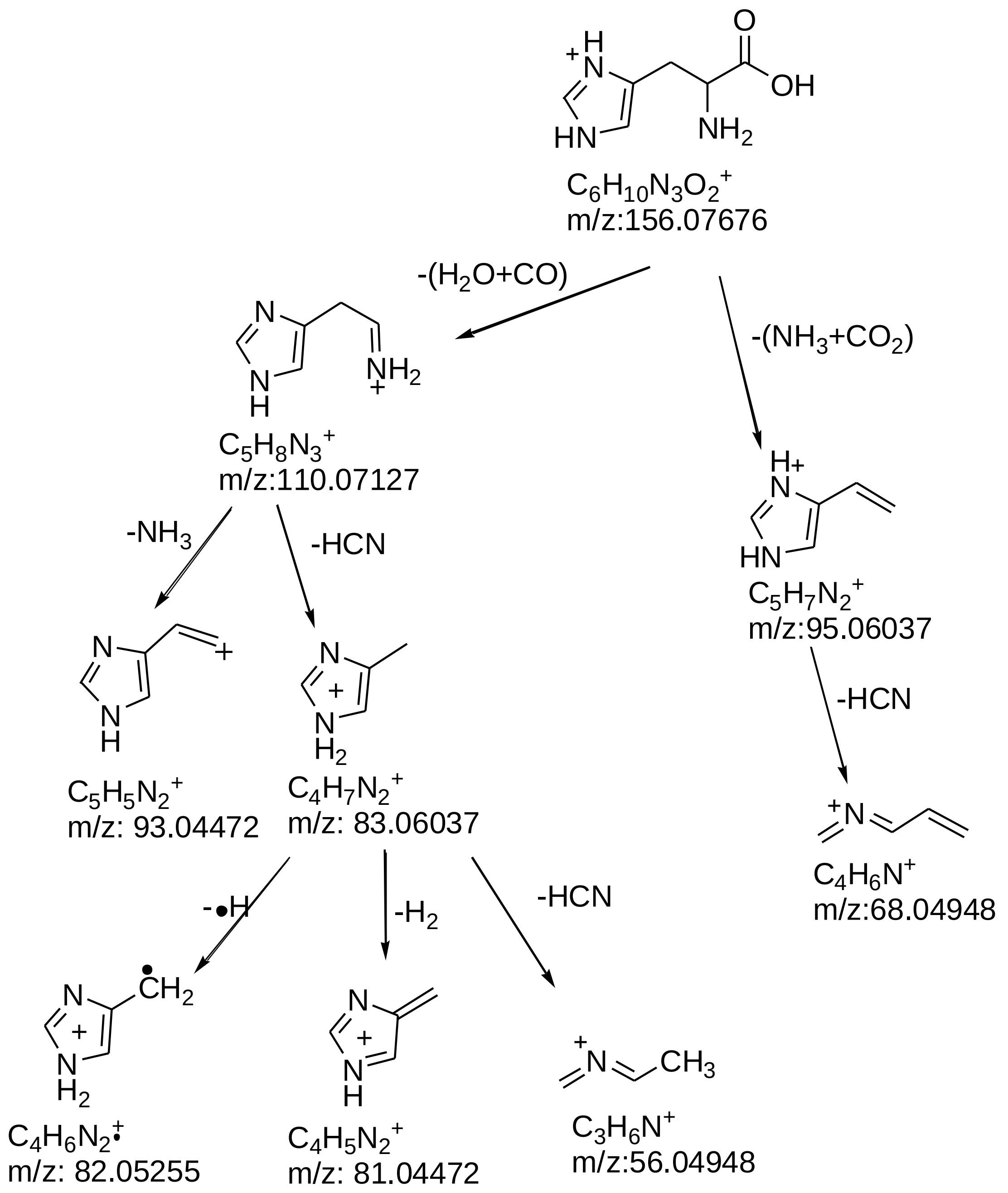

Supplementary Figure S49. Postulated fragmentation pathways of protonated His. The theoretical *m/z* value of each fragment ion is provided under the chemical formula.

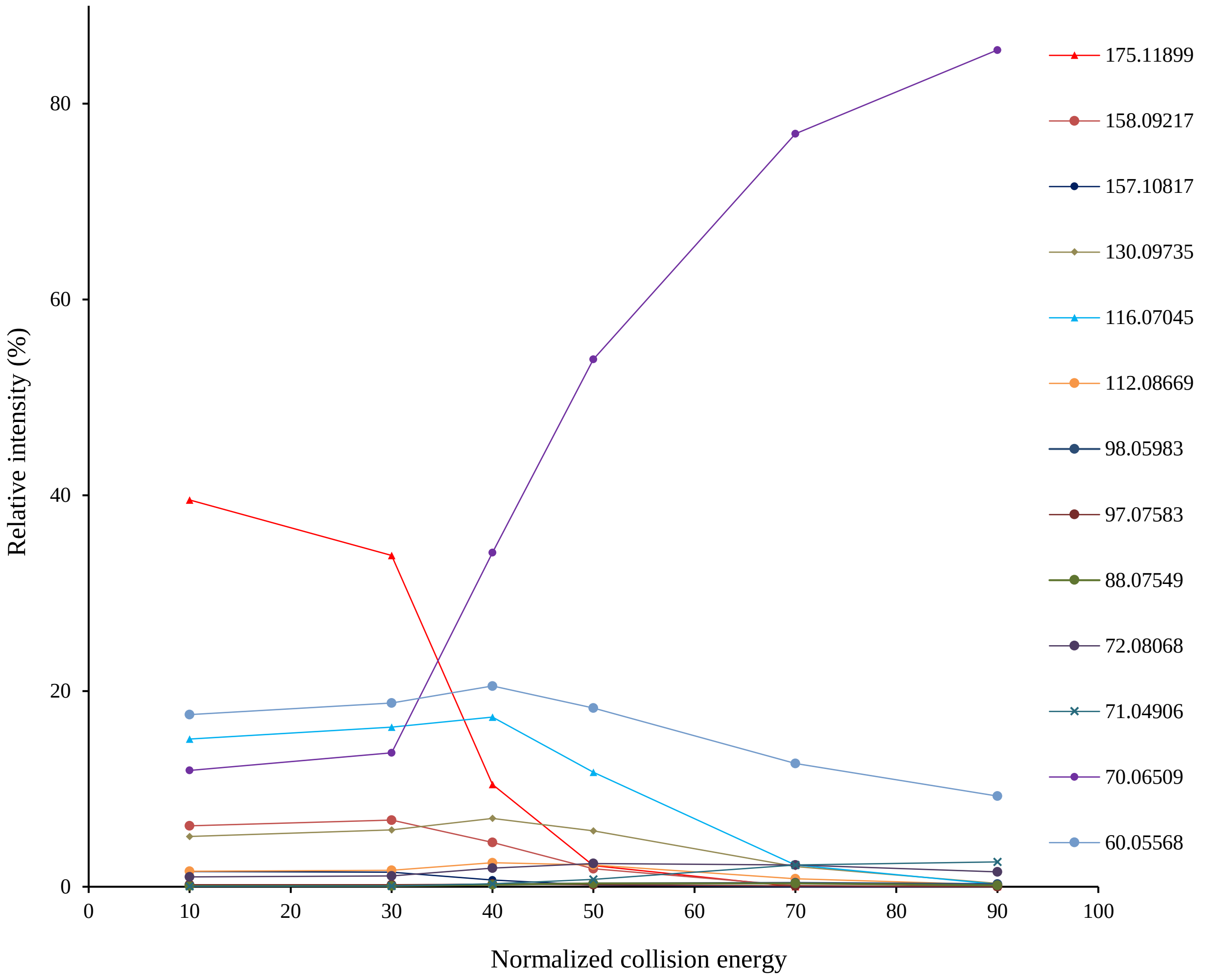

Supplementary Figure S50. Fragmentation graph of protonated Arg under different collision energies.

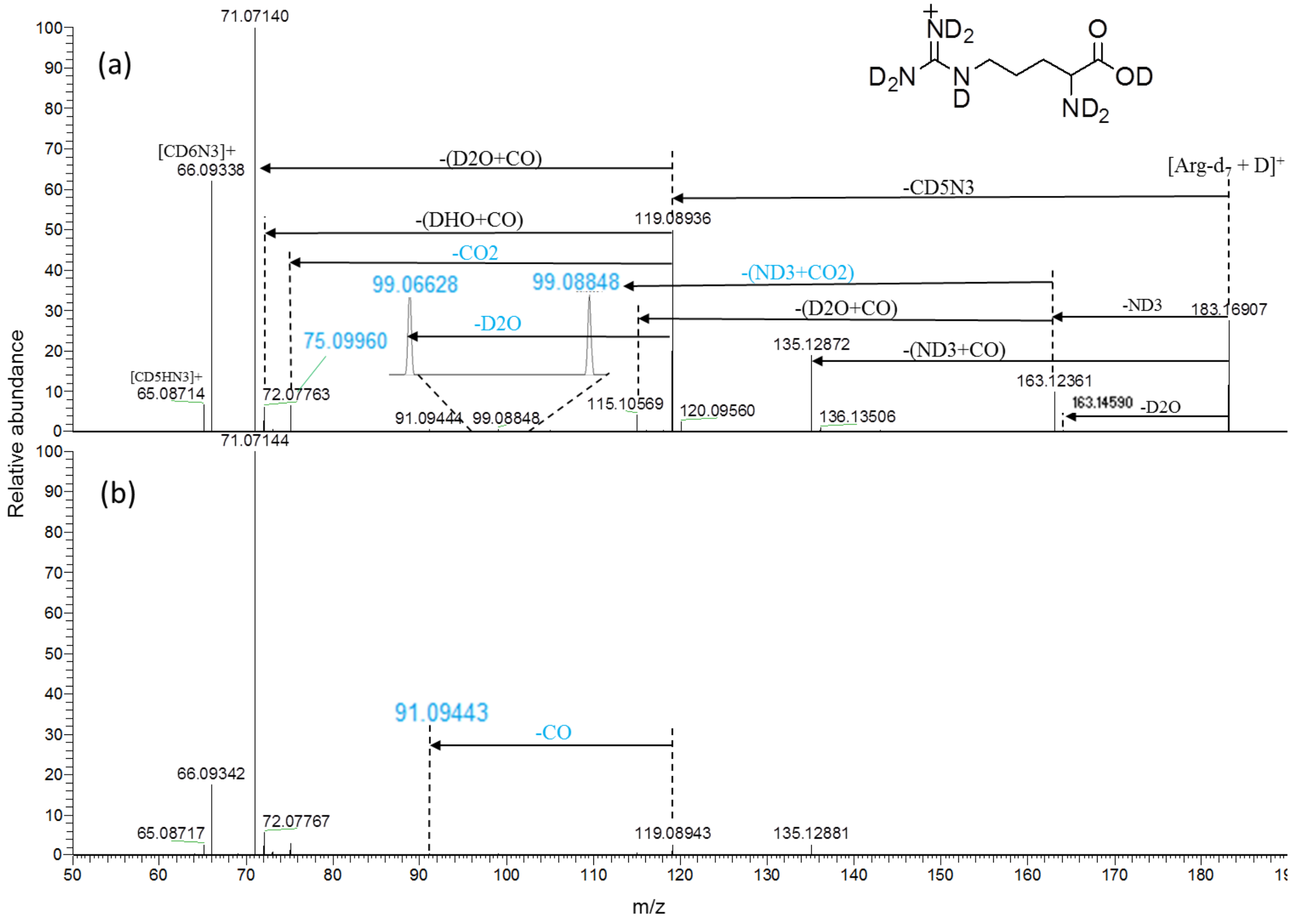

Supplementary Figure S51. Representative MS/MS spectra of $[Arg\text{-}d_7 + D]^+$ acquired using collision energy NCE 30% (a) and 70% (b). The four previously unreported fragments are shown in blue.

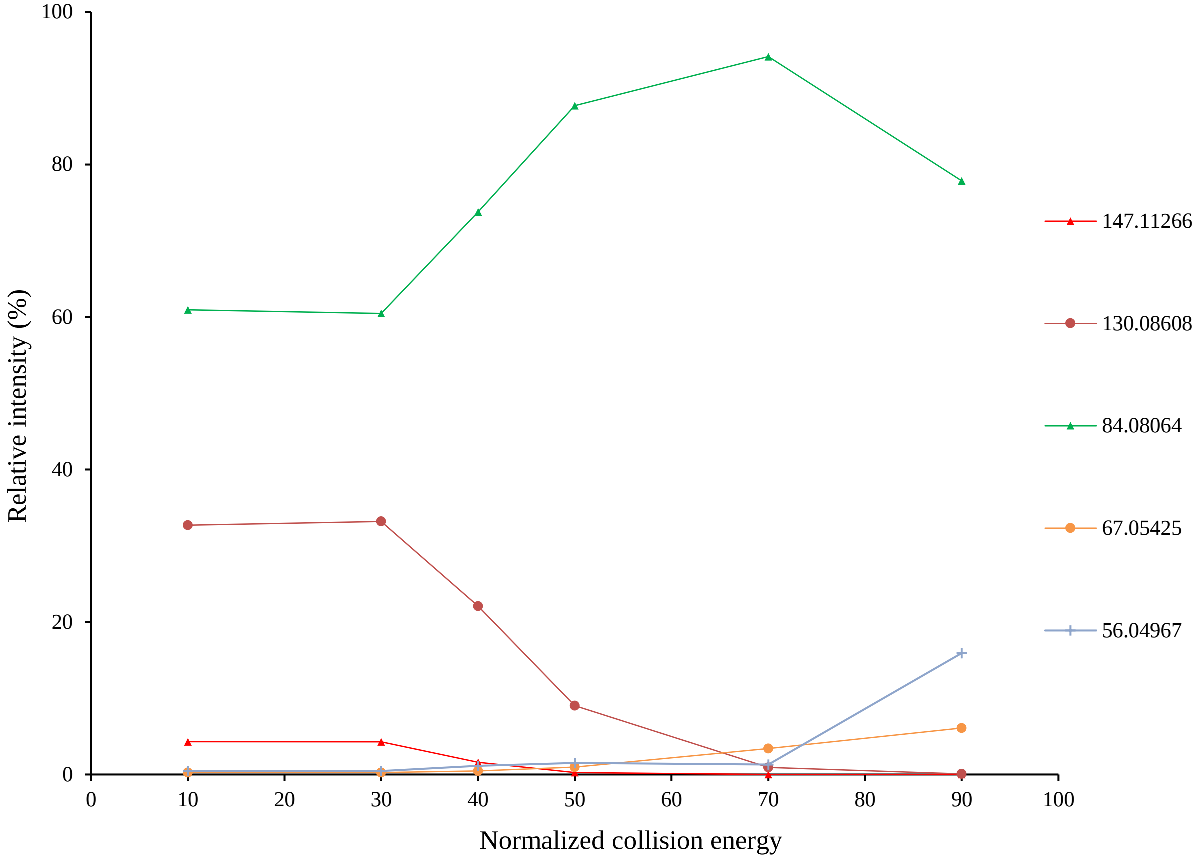

Supplementary Figure S52. Fragmentation graph of protonated Lys under different collision energies.

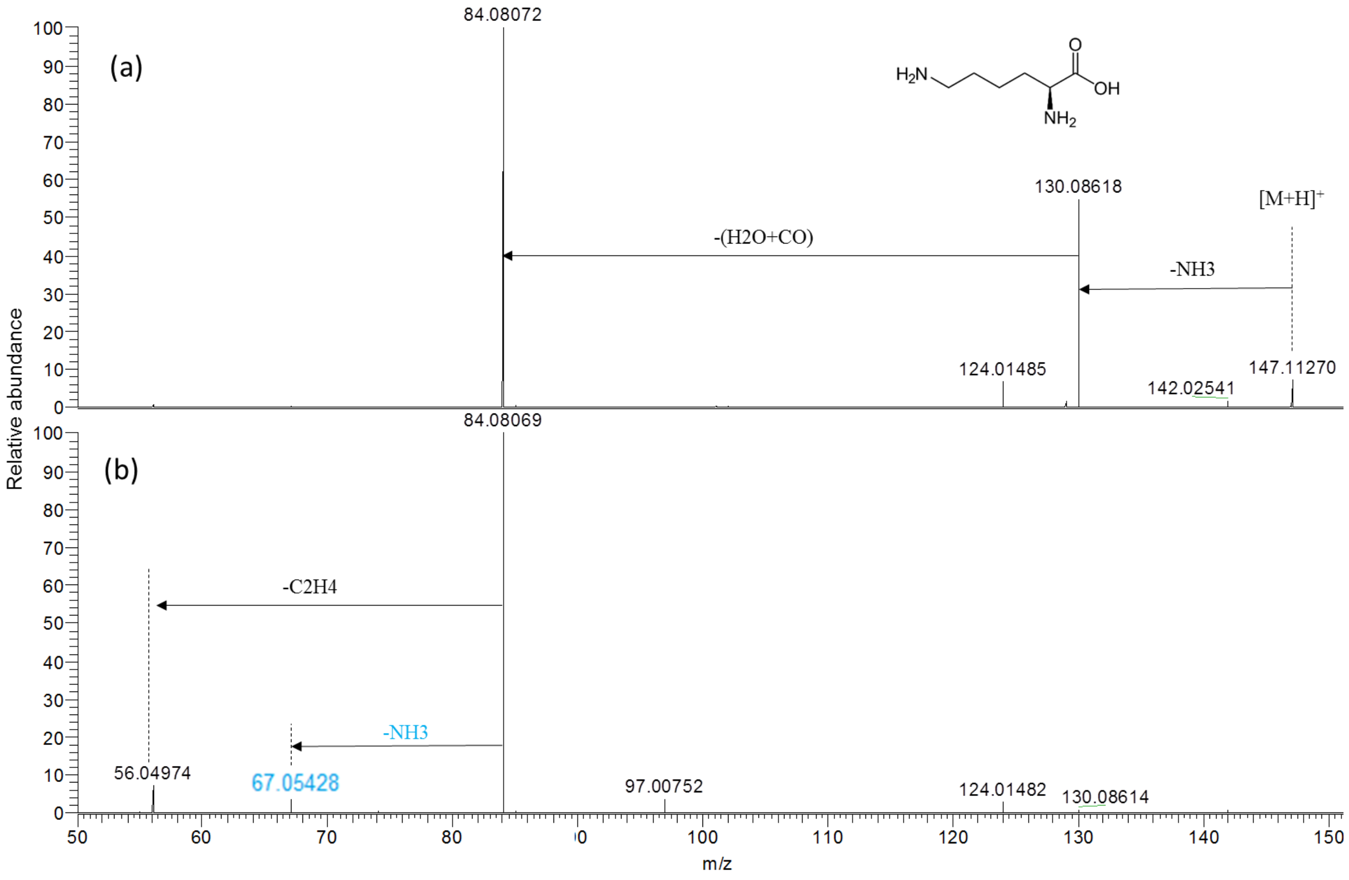

Supplementary Figure S53. Representative MS/MS spectra of protonated Lys acquired using collision energy NCE 30% (a) and 70% (b). A previously unreported fragment ion is shown in blue.

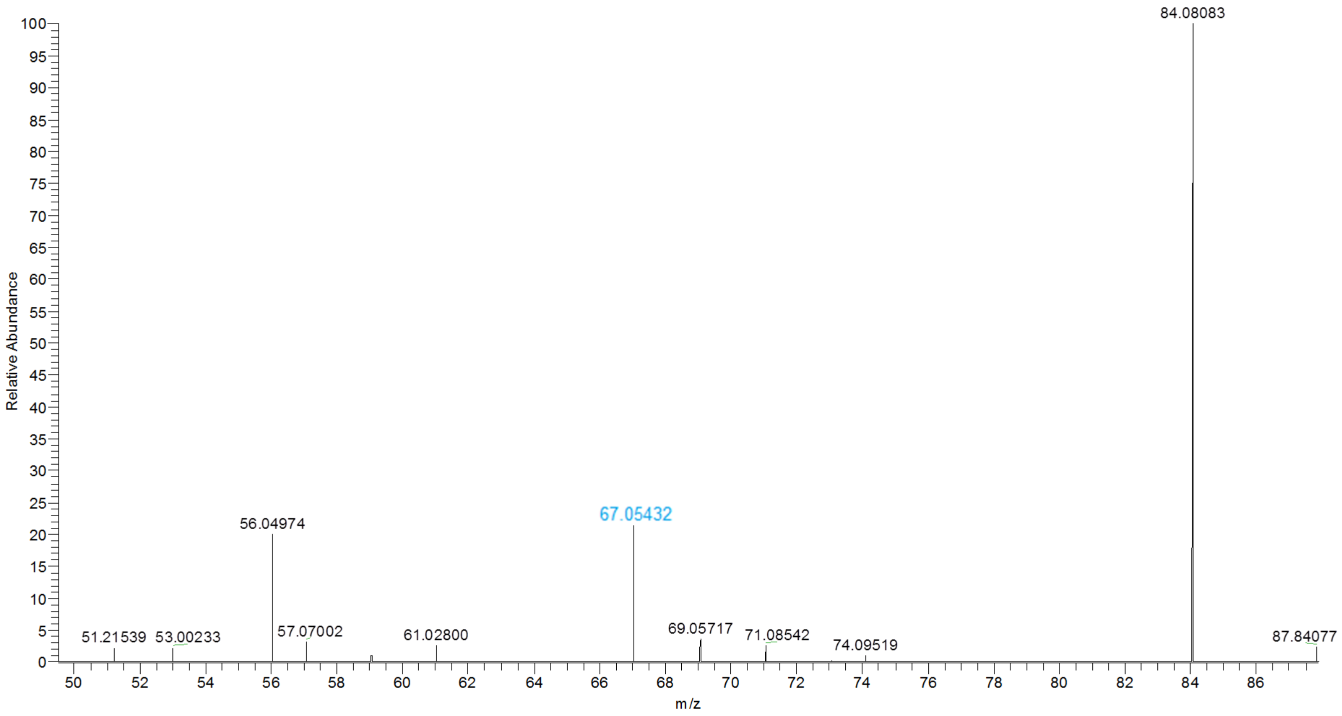

Supplementary Figure S54. Pseudo MS^3 of the fragment ion at *m/z* 84.08064 from protonated Lys. The fragment ion at *m/z* 56.04967 and the previously unreported fragment ion at *m/z* 67.05425 were observed.

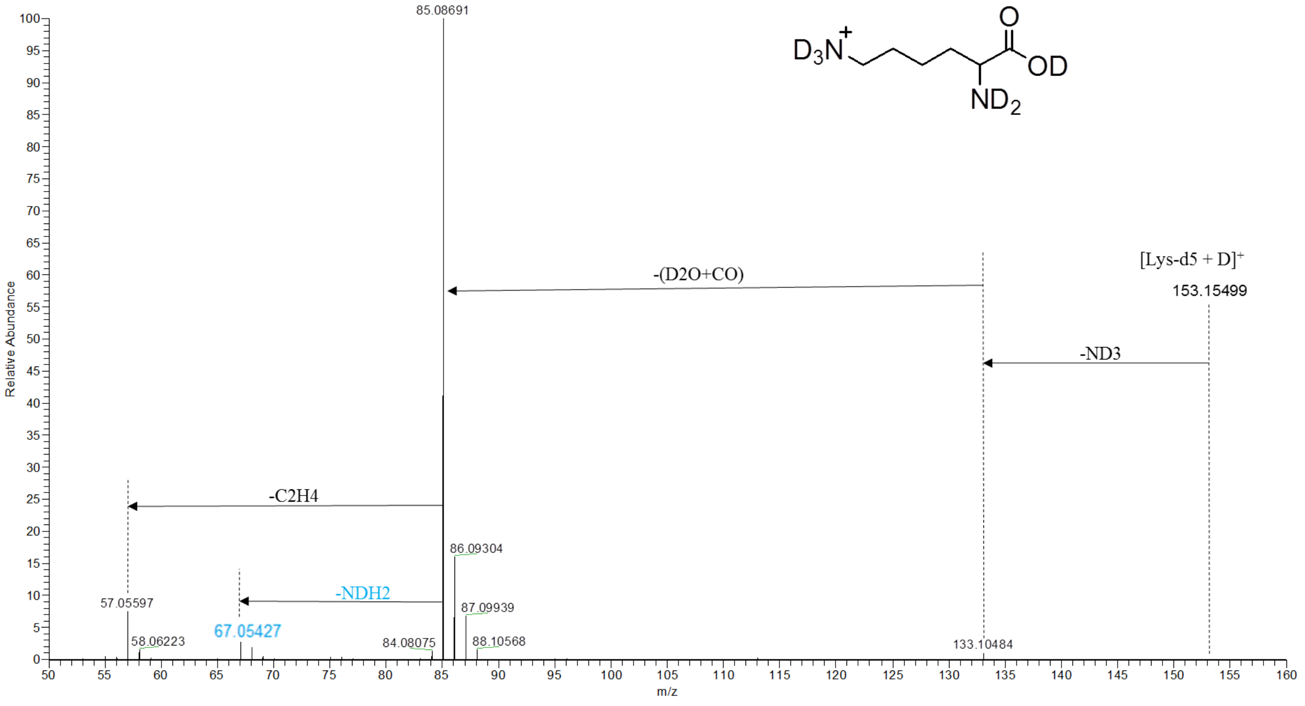

Supplementary Figure S55. Representative MS/MS spectra of $[Lys\text{-}d_5 + D]^+$ under collision energy NCE 70%. The previously unreported fragment ion is shown in blue.

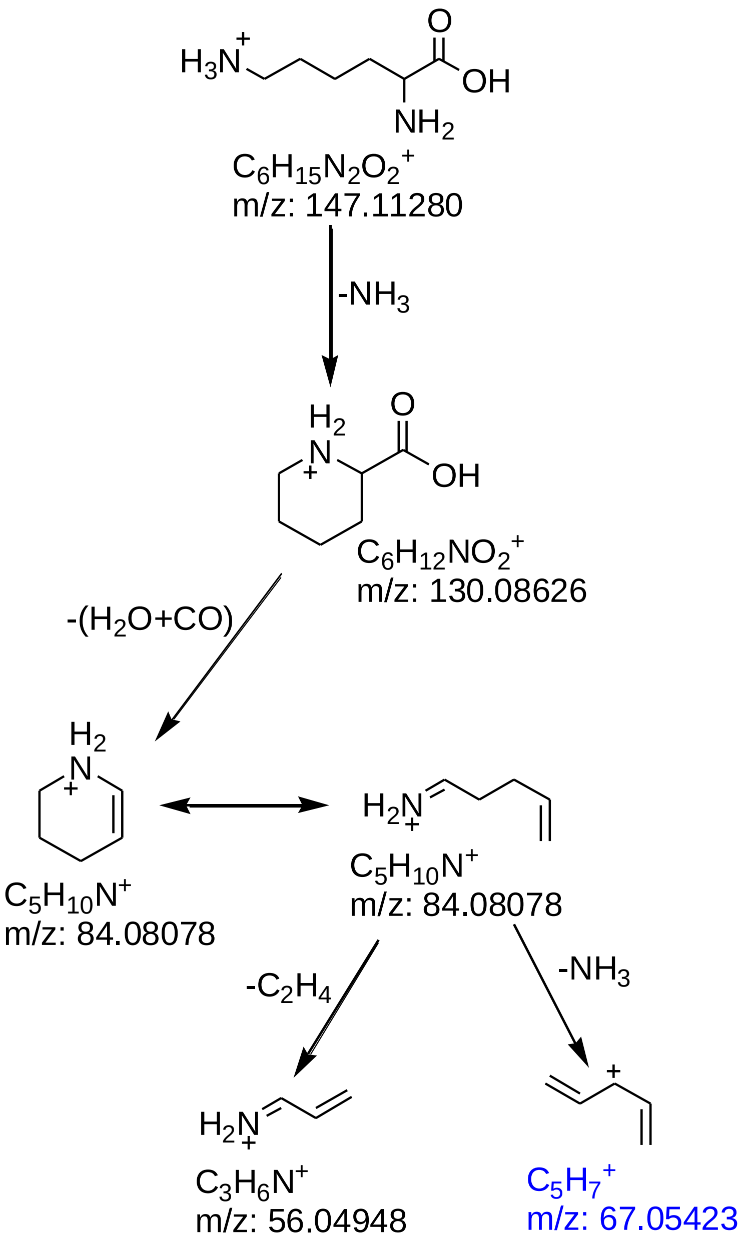

Supplementary Figure S56. Postulated fragmentation pathway of protonated Lys. The previously unreported fragment ion is shown in blue. The theoretical *m/z* value of each fragment ion is provided under the chemical formula.

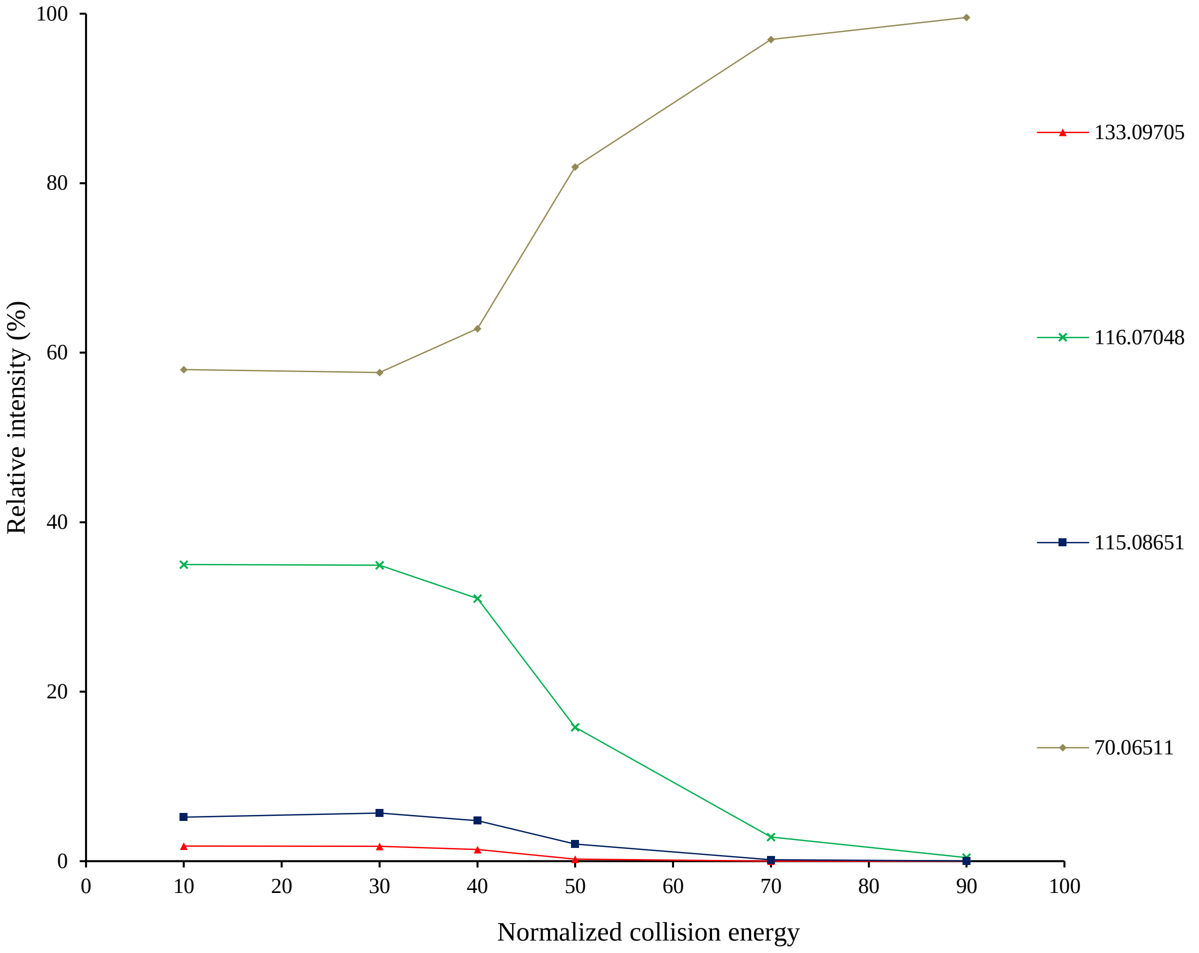

Supplementary Figure S57. Fragmentation graph of protonated ornithine under different collision energies.

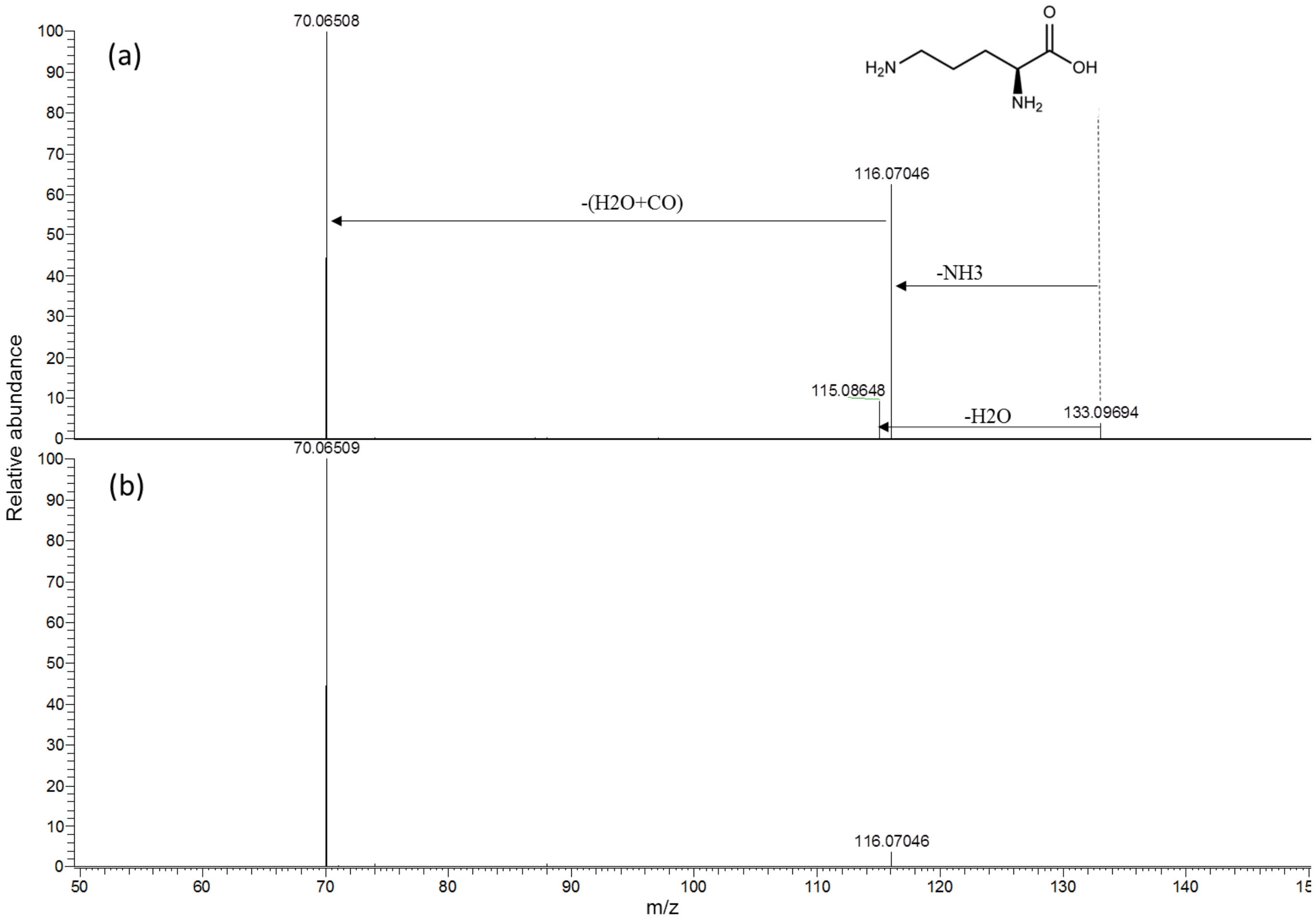

Supplementary Figure S58. Representative MS/MS spectra of protonated ornithine acquired using collision energy NCE 30% (a) and 70% (b).

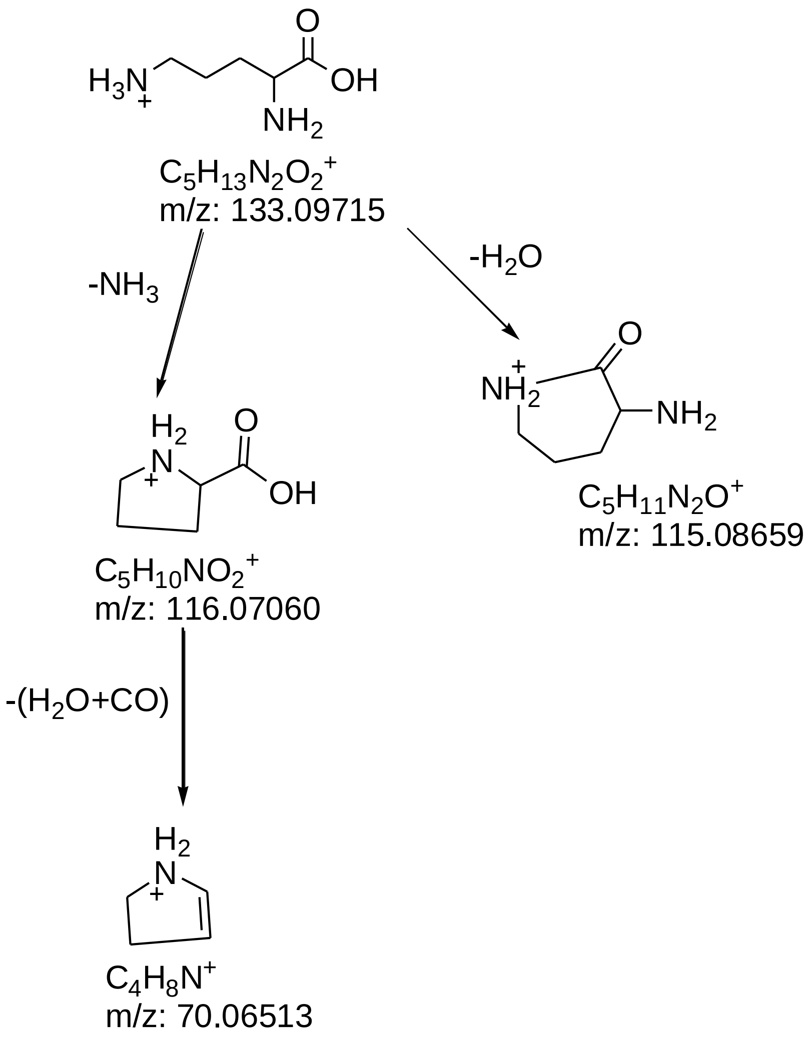

Supplementary Figure S59. Postulated fragmentation pathways of protonated ornithine. The theoretical *m/z* value of each fragment ion is provided under the chemical formula.

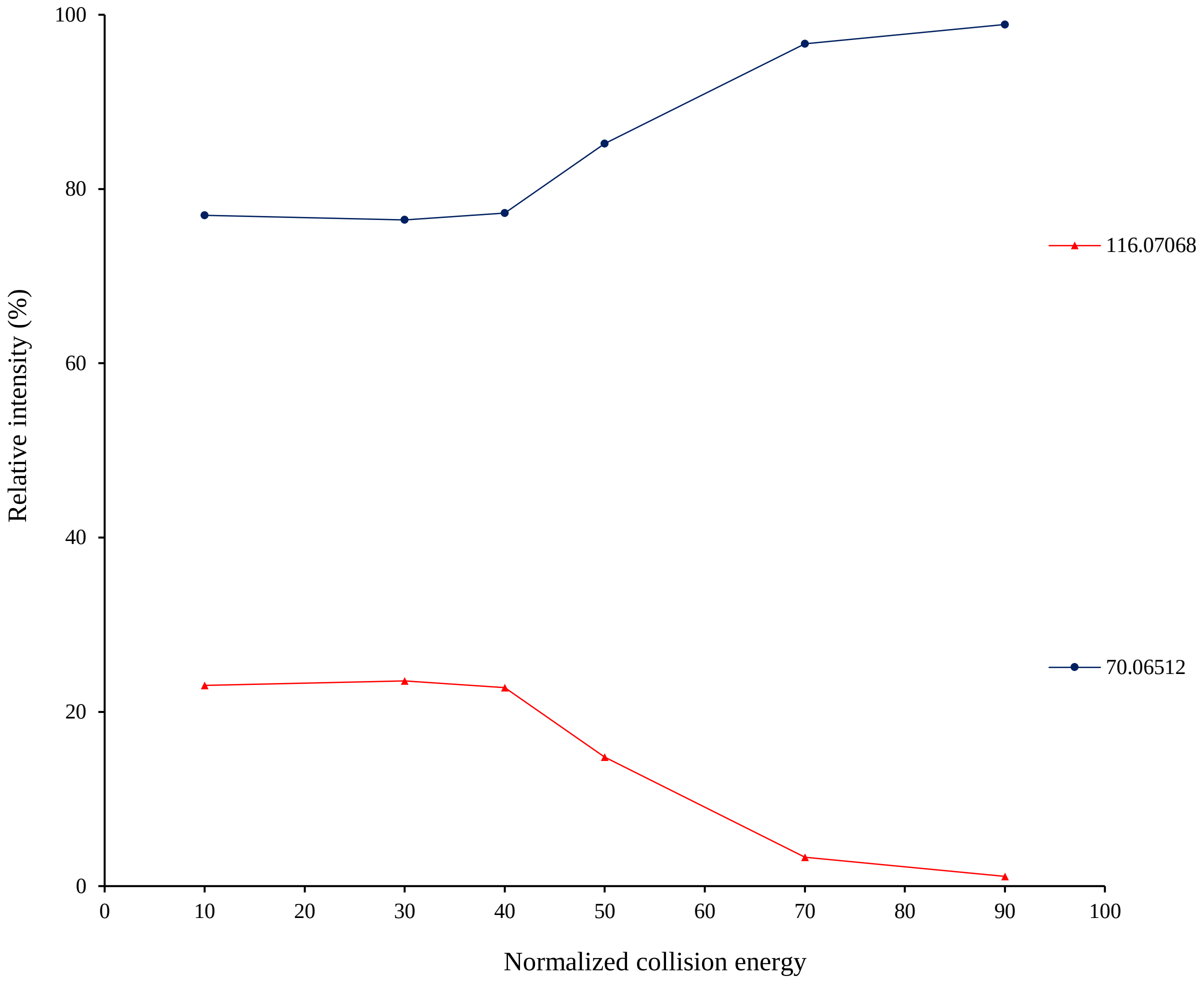

Supplementary Figure S60. Fragmentation graph of protonated Pro under different collision energies.

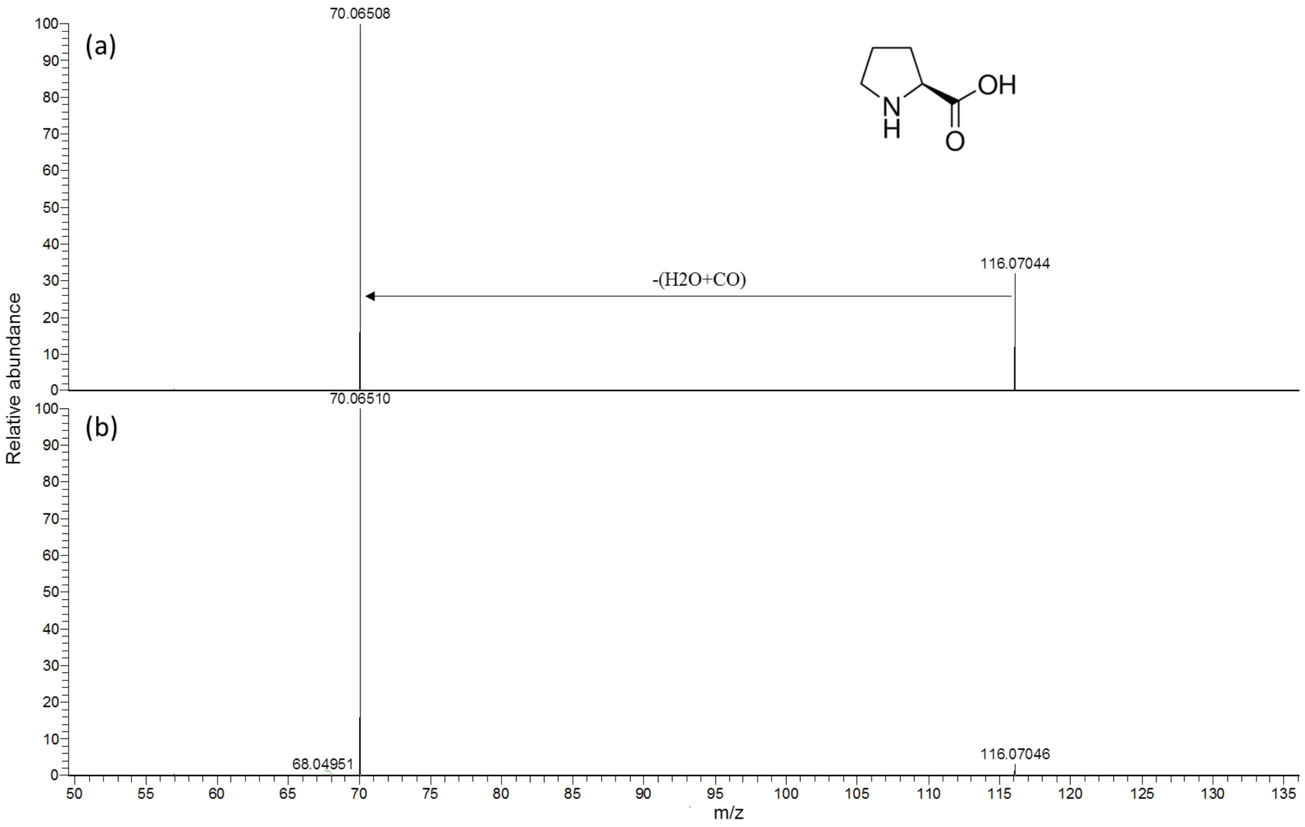

Supplementary Figure S61. Representative MS/MS spectra of protonated Pro acquired using collision energy NCE 30% (a) and 70% (b).

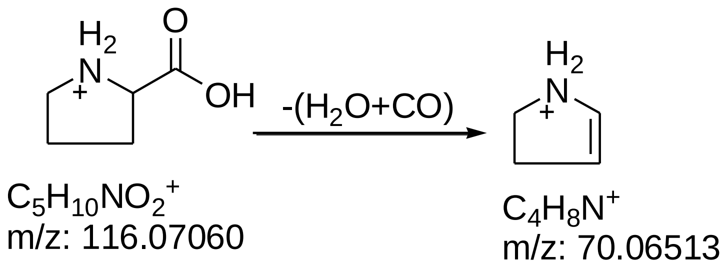

Supplementary Figure S62. Postulated fragmentation pathway of protonated Pro. The theoretical *m/z* value of each fragment ion is provided under the chemical formula.
